# Regioselective Cycloaddition of Nitrile Imines to 5-Methylidene-3-phenyl-hydantoin: Synthesis and DFT Calculations

**DOI:** 10.3390/ijms24021289

**Published:** 2023-01-09

**Authors:** Maria E. Filkina, Daria N. Baray, Elena K. Beloglazkina, Yuri K. Grishin, Vitaly A. Roznyatovsky, Maxim E. Kukushkin

**Affiliations:** Department of Chemistry, M.V. Lomonosov Moscow State University, Leninskie Gory, 1-3, 119991 Moscow, Russia

**Keywords:** imidazolones, thiohydantoins, spiro-compounds, 1,3-dipolar cycloaddition, nitrile imines

## Abstract

Nitrile imine cycloaddition to hydantoins containing an exocyclic C=C double bond has been previously described in a very limited number of examples. In this work, regioselective synthesis of spiro-pyrazoline-imidazolidine-2,4-diones based on a 1,3-dipolar cycloaddition reaction of nitrile imines to 5-methylidene-3-phenyl-hydantoin have been proposed. It was found that, regardless of the nature of the aryl substituents at the terminal C and N atoms of the C-N-N fragment of nitrile imine (electron donor or electron acceptor), cycloaddition to the 5-methylidenhydantoin exocyclic C=C bond proceeds regioselectively, and the terminal nitrogen atom of the nitrile imine connects to the more sterically hindered carbon atom of the double bond, which leads to the formation of a 5-disubstituted pyrazoline ring. The observed cycloaddition regioselectivity was rationalized using DFT calculations of frontier molecular orbital interactions, global CDFT reactivity indices, and minimum energy paths.

## 1. Introduction

Pyrazoles and pyrazolines are known to exhibit a wide range of biological activity and often occur in various types of compounds with antibacterial, antimicrobial [1], antidepressant, anti-inflammatory, antiviral [2], analgesic, immunosuppressive, and anticancer [3] properties. Pyrazoles and pyrazolines can be easily obtained via the 1,3-dipolar cycloaddition reaction of nitrile imines to corresponding dipolarophiles [4,5]. In pioneering work on this transformation, Huisgen suggested a convenient approach to pyrazoline ring construction based on the [3+2]-cycloaddition reaction of nitrile imines to alkenes [6]. Reactive 1,3-dipoles may be generated in situ by base-promoted dehydrohalogenation of hydrazonoyl chlorides [7] or by thermal or photoinduced decomposition of tetrazoles [8]. However, tetrazoles are hazardous compounds, and some difficulties in their synthesis make the last approach less suitable for practical applications. Hydrazonoyl halides turned out to be more convenient sources of 1,3-dipoles and are widely used in pyrazoline/pyrazole synthesis.

Nitrile imines vary greatly in reactivity towards the carbon-carbon double bonds, which can be influenced by both electronic and steric effects. Increased steric bulk in dipolarophile has shown a detrimental effect on reaction rate. Monosubstituted alkenes usually react easily with nitrile imines [7]. Disubstituted alkenes are typically competent substrates, with trisubstituted olefins reacting more sluggishly [9]. Nitrile imines are also effectively applied in the cycloaddition reaction with exocyclic alkenes [10] to form spirocyclic products containing 4-substituted [11] or 5-substituted pyrazole/pyrazoline cycles [12]. However, it has been shown that generally intermolecular nitrile imine cycloadditions favors the formation of a regioisomer with the most sterically encumbered substituent of the dipolarophile in the 5-position of the resulting heterocycle [13,14].

[3+2]-Cycloaddition processes are typical pericyclic reactions; they are generally characterized by a large negative entropy of activation, which indicates the presence of a highly organized transition state during the reaction and leads to high regioselectivity [15]. Resulting regioselectivity is considered using frontier molecular orbital theory (FMO) through calculations of the relative electron density of the corresponding HOMO and LUMO orbitals at each of the reaction centers of nitrile imines and dipolarophiles. This showed that 5-substituted heterocycles were favored in the vast majority of cases since the orbital coefficient on the carbon atom on the LUMO of nitrile imine is much higher than that on nitrogen. As a result, a more efficient overlap is realized between the orbitals of the carbon atom of the C-N-N fragment of the 1,3-dipole and the carbon atom of the double bond, which does not contain substituents [13].

**Scheme 1:**
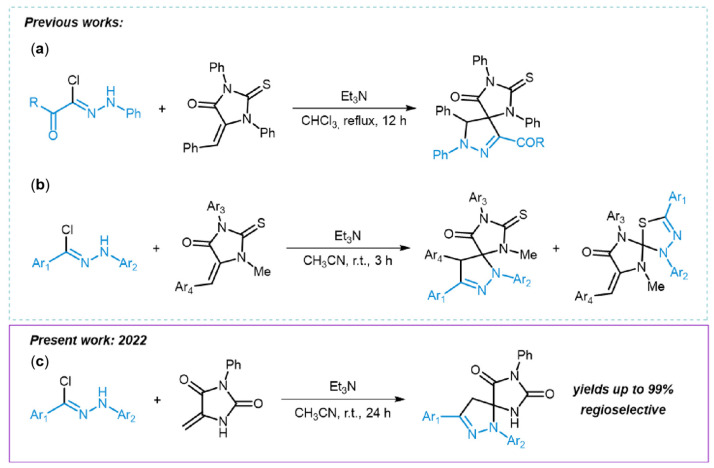
Synthetic approaches to the regioselective 1,3-dipolar cycloaddition reaction of nitrile imines to thiohydantoins and hydantoin derivatives. (**a**,**b**) previous works [16,17]; (**c**) present work.

There are in the literature a few examples of the addition of nitrile imines to exocyclic double bonds of derivatives of 5-methylidene-2-chalcogen-imidazolones (Scheme 1). Hassaneen in 1995 [16], demonstrated the possibility of regioselective nitrile imine addition to trisubstituted double C=C bonds of 1,3-diphenyl-5-phenylmethylene-2-thiono-4-imidazolidinones (arylidenethiohydantoins). These reactions lead to the formation of the spiro-compound, which contains a 4-substituted pyrazoline ring, which is unusual. In recent work of Yavari [17], the cycloaddition of nitrile imines to 5-arylmethylene-2-thiohydantoins, leading to the formation of a mixture of two products containing a 5-substituted pyrazoline fragment and a 1,2,4-thiazoline fragment, respectively, was shown. The cycloaddition of nitrile imines to hydantoins containing a 1,1-disubstituted exocyclic carbon-carbon double bond was first described in previous work of our scientific group [18]. In the literature there are also a few examples of the addition of nitrile imines to exocyclic double C=C bonds of other types [19,20,21]. In the present article, the [3+2]-cycloaddition of nitrile imines to 5-methylidene-2-phenylhydantoin and a study of its regioselectivity are described.

Note that compounds containing spiro-linked heterocyclic fragments are of interest from the point of view of studying their biological activity. It was shown that the presence of spiro-jointed fragments leads to an increase in the conformational rigidity of molecules, resulting in an increase in their affinity for target proteins [22,23]. The development of synthetic approaches to such structures is an actual task of organic and medicinal chemistry.

## 2. Results and Discussion

### 2.1. Synthesis

**Scheme 2:**
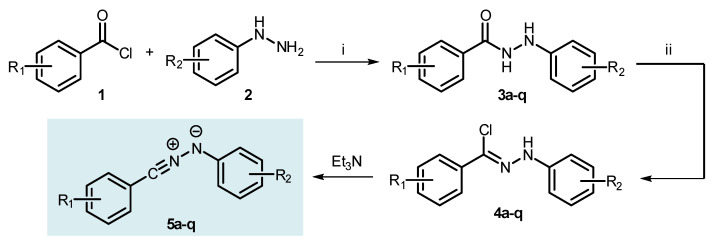
Synthesis of hydrazonoyl chlorides **4** and base-induced generation of nitrile imines **5**. Reaction conditions: (i) Benzoyl chloride **1** (1 equiv), arylhydrazine **2** (1.1 equiv), pyridine (2,2 equiv), THF, 0–5 °C, 2 h; (ii) acylhydrazine **3** (1 equiv), triphenylphosphine (1.25 equiv), carbon tetrachloride (1.25 equiv), CH_3_CN in an inert atmosphere at room temperature overnight.

We started our study with the 1,3-dipolar cycloaddition of nitrile imines **5** to the exocyclic carbon-carbon 1,1-disubstituted double bond of 5-methylidene-3-phenylhydantoin **6**, prepared according to a previously proposed method [24]. Nitrile imines **5** can be generated in situ by base-induced dehydrohalogenation of hydrazonoyl chlorides **4** in the presence of Et_3_N (Scheme 2). 

A series of the initial hydrazonoyl chlorides **4** was prepared using commercially available benzoic acids and phenylhydrazines **2** (Scheme 2). Following the general protocol [25], phenylhydrazine derivatives **2** were acylated with benzoyl chlorides **1** to form acylhydrazines **3**. Subsequent chlorination of **3** with triphenylphosphine and carbon tetrachloride in anhydrous acetonitrile gave the desired nitrile imine precursors **4a-q** in a reasonable to high overall yield (see Supplementary Information pp. 2–12).

For the optimization of spiro-cyclization conditions, hydrazonoyl chloride **4a** was selected as a model substrate, and its cycloaddition reaction with dipolarophile **6** was performed using different solvents (methylene chloride, chloroform, acetonitrile, ethyl acetate, benzene, and toluene) and reagent ratios at room temperature and under reflux (Table 1). In a test experiment, we conducted the reaction using a slight excess of nitrile imine **5a** (1.1 equiv.), which was generated in the presence of dipolarophile **6** (1.0 equiv.) by dropwise addition of Et_3_N (2.2 equiv.) in dry methylene chloride at room temperature. In these conditions the spiro-pyrazoline **7a** was isolated with an 88% yield (Table 1, entry 2) by column chromatography on silica gel. The presence of an excess of nitrile imine **5a** (2.0 equiv.) does not improve the yield of cycloaddition product but rather reduces it to 58% (Table 1, entry 3), which may be caused by the irreversible dimerization of nitrile imine with its significant excess in the reaction mixture [26]. The reaction under refluxing benzene provided the same product (**7a)** in a shorter time (12 h) and with a good yield (82%). However, with strong heating of the system, the rate of dimerization of 1,3-dipole increases, and therefore the yield of the product **7a** decreases (44%), when the reaction is carried out in toluene (Table 1, entries 5,6). The optimal conditions, in terms of yield (99%) and purity of the final product **7a**, were achieved by carrying out the [3+2]-cycloaddition in an anhydrous acetonitrile at room temperature in the presence of a slight excess (1.1 equiv.) of 1,3-dipole for 48 h (Table 1, entry 4).

**Scheme 3:**
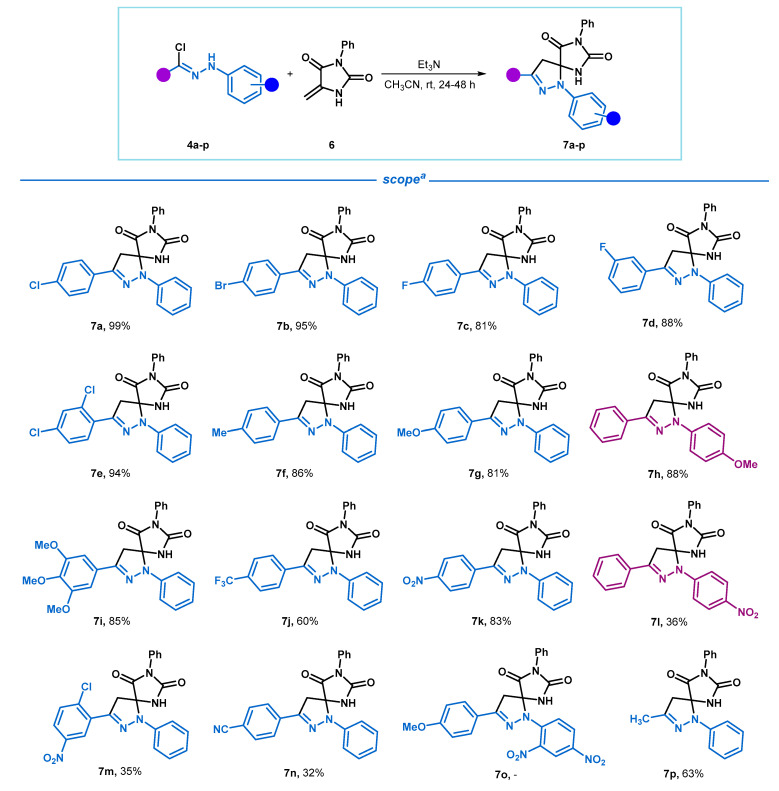
Scope of the reaction. ^a^ Isolated yields.

Employing the optimal conditions, hydrazonoyl chlorides **4a-p** containing various substituents at both the carbon atom and terminal nitrogen atom of the C-N-N fragment were introduced into the reaction with 5-methylidene-3-phenylhydantoin **6** (Scheme 3). As a result, the spiro-pyrazoline-imidazolidine-2,4-diones **7a-k**, and **7p** were obtained in good yields. The presence of electron-donating substituents in both aromatic rings at the carbon atom and terminal nitrogen atom of the C-N-N fragment of nitrile imine leads, in most cases, to an increase in the yield of the cycloaddition product. At the same time, the introduction of electron-withdrawing substituents into the carbon connected ring lowered the yields of cycloaddition. The low yields of products **7m** and **7n** are presumably associated with a decrease in the rate of the cycloaddition process, since in this case, even with an increase in the reaction time from 24 h to 5 days, unreacted starting reagents were found in the reaction mixture. This is consistent with the data obtained in the Wang work [27], according to which the introduction of electron-withdrawing substituents reduces the rate of the cycloaddition reaction. 

**Scheme 4:**
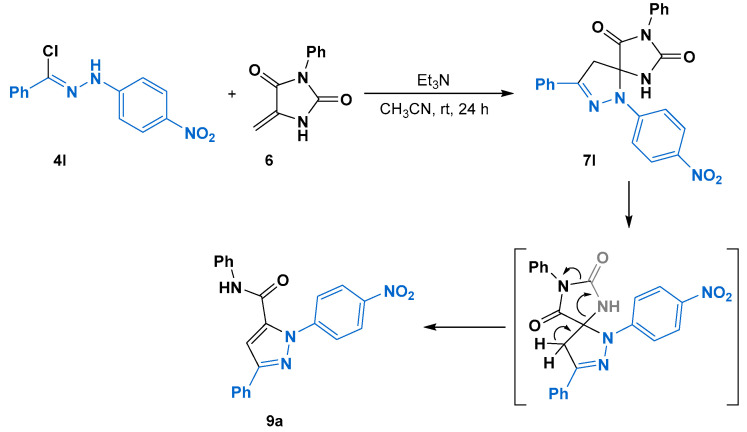
The proposed mechanism for the formation of the by-product **9a**.

At the same time, in the case of compounds **7k** and **7l**, which contain an electron-withdrawing nitro-group in the *para*-position of the aromatic ring at the carbon atom (compound **7k**) or in the ring at the terminal nitrogen atom of the C-N-N fragment (compound **7l**), there is a significant difference in the cycloaddition product yields (83% and 36%, respectively). The decrease in the yield of the compound **7l** is presumably due to the side reaction of pyrazole **9a** formation (Scheme 4). Apparently, the pyrazole derivative **9a** is formed as a result of cleavage of the imidazolone fragment of the spiro compound **7l**, accompanied by aromatization of the pyrazole fragment, similarly to that described in [28,29]. However, it should be noted that an increase in the reaction time does not lead to a significant increase in the yield of the side product **9a**. The structure of compound **9a** was confirmed by 2D NMR spectroscopy and HRMS (see Supplementary Information pp. 38–40).

In the case of nitrile imine **4o**, which simultaneously contains an electron-withdrawing substituent at the nitrogen atom and an electron-donating substituent at the carbon atom of the C-N-N fragment of nitrile imine, the reaction with dipolarophile **6** resulted in the formation of a complex mixture of different products, among which the target compound **7o** was not found. Presumably, the presence in two benzene rings of a dipole formed from hydrazonyl chloride **4o**, functional groups with opposite electronic effects (mehomeric donor OCH_3_ and mehomeric acceptors NO_2_) leads to its significant polarization and an increase in reactivity, which explains the formation of a complex mixture of the products.

The structures of the resulting spiro derivatives **7j-l** were established using 2D NMR spectroscopy with HSQC, HMBC, and NOESY (see Supplementary Information pp. 20–43). Based on the 2D NMR data, the spatial proximity of the CH_2_ group of the pyrazoline ring and the aromatic cycle containing the CF_3_ group in the case of compound **7j** and the NO_2_ group in the case of compound **7k** was established. In contrast, for compound **7l**, the unsubstituted aromatic ring at the carbon atom of C-N-N fragment is neighboring with the CH_2_ group of the pyrazoline ring. In the ^1^H NMR spectra of the reaction mixtures for all compounds, a single set of signals was observed corresponding to the products, as shown in Scheme 3. As a result, it was shown that, regardless of the nature of the substituents at the carbon atom or the terminal nitrogen atom of the C-N-N fragment of nitrile imine, cycloaddition to the exocyclic double bond of 5-methylidenhydantoin **6** proceeds regioselectively. In all cases, the terminal nitrogen atom of the C-N-N fragment of nitrile imine connects to the more sterically hindered quaternary carbon atom of the double C=C bond, which leads to the formation of a 5-disubstituted pyrazoline ring. 

Thus, the [3+2] cycloaddition of the in situ formed hydrazonoyl chloride-derived nitrile imines with 5-methilidene-3-phenylhydantoin, containing 1,1-disubstituted exocyclic carbon-carbon double bond, proceeded easily and furnished structurally novel spiro-pyrazoline-imidazolidine-2,4-diones **7a-p** in reasonable chemical yields. Furthermore, it was shown that cycloaddition proceeds regioselectively with the formation of a single regioisomer.

To further investigate the observed regioselectivity of [3+2]-cycloaddition of nitrile imines to dipolarophile **6** we decided to proceed with quantum chemistry calculations. The reaction of substituted nitrile imines **5a, j-l** with 5-methilidene-3-phenylhydantoin **6** yielding spiro-pyrazoline-imidazolidine-2,4-diones **7a-k** has been studied within density functional theory (DFT) at the PBE0/def2-SVPD computational level.

### 2.2. DFT Calculations

In terms of theoretical aspects concerning regio- and stereoselectivity, the 1,3-dipolar cycloaddition reaction of nitrile imines has always attracted the attention of researchers. In several works, the regioselectivity of the [3+2]-cycloaddition reaction of nitrile imines was calculated using DFT methods [30], but, in most cases, the calculations were carried out only for mono- or disubstituted alkenes [31]. Quantum chemical calculations using the B3LYP/6-311+G(d+p) method have demonstrated that the regioselectivity of the process corresponds to the overlap of orbitals at the reaction centers with the highest orbital coefficients [32]. In the present work, we calculated the cycloaddition of nitrile imines to the 1,1-disubstituted C=C double bond of hydantoin **6** using classical approaches to determining the regioselectivity of the process by calculation methods.

We carried out all DFT-calculations with the ORCA program package [33]. The geometries of all reactants and transition state structures were optimized by using DFT with the PBE0 hybrid exchange correlation functional and def2-svpd basis set [34,35]. HOMO and LUMO energies and corresponding global reactivity indices were achieved by single-point calculations on the PBE0/def2-svpd optimized geometries. To make calculations more realistic, solvation effects (using dichloromethane as solvent) have been taken into account using the conductor-like polarizable continuum model (CPCM) [36,37]. Dichloromethane was chosen as a model solvent for a correct comparison with the available literature data, since most of the literature data for DFT modeling of reactions involving nitrile imines are given for dichloromethane [38,39]. In the calculations of the condensed Fukui functions [40], the cationic and anionic systems were kept at the optimized geometries of the corresponding neutral systems. Electronic populations were analyzed by the Hirshfeld [41] charges. To localize the transition state (TS), we applied the nudged elastic band method (NEB) [42]. 

Our investigation consisted of four steps:

1. Comparison of the energies of the frontier orbitals of the reagents to determine the nature of the interaction between the 1,3-dipole and the dipolarophile.
2. Analysis of the global and local reactivity indices of the ground states is necessary to better understand preferable regioselectivity.
3. Determination of Fukui functions to predict the most favorable regioisomeric reactive channel.
4. Calculation of the minimum energy path (MEP), localization of the transitional states, and determination of the corresponding geometries.

#### 2.2.1. Frontier Molecular Orbital Interaction

Concerted 1,3-DC reactions can be conveniently interpreted using the FMO method [15]. On the basis of FMO theory, the cycloaddition processes are categorized into three different types [43,44]. Type I reactions are controlled by the interaction between the highest occupied molecular orbital of the dipole (HOMO_dipole_) and the lowest unoccupied molecular orbital of the dipolarophile (LUMO_dipolarophile_). These are also known as normal-electron demand (NED) 1,3-DC reactions. Type II reactions are controlled by the interaction between the LUMO of the dipole and the HOMO of the dipolarophile. They are also named as inverse-electron demand (IED) 1,3-DC reactions. Finally, type III reactions may be characterized by the similarity of the HOMO and LUMO energies of the dipole/dipolarophile pair. In this case, both HOMO_dipole_–LUMO_dipolarophile_ and HOMO_dipolarophile_–LUMO_dipole_ interactions may be important in determining the reactivity and regioselectivity of the process, and both NED and IED can take place.

**Figure 1:**
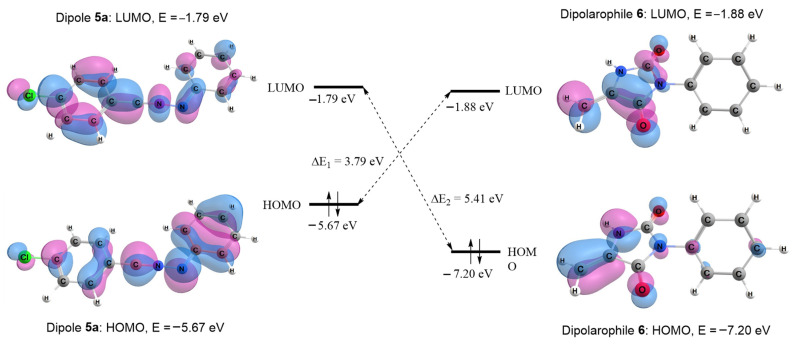
Optimized geometries, visualized FMOs, and graphical illustration of the HOMO-LUMO interaction of nitrile imine **5a** and 5-methylidene-3-phenylhydantoin **6**.

The energy differences between the frontier molecular orbitals of nitrile imines **5a, j-l,** and dipolarophile **6** were calculated. The results presented in Figure 1 and Table 2 show that the energy gap between the HOMO of dipolarophile **6** and the LUMO of 1,3-dipole **5a** is 5.41 eV, which is significantly larger than the interaction between the HOMO of 1,3-dipole **5a** and the LUMO of dipolarophile **6** (3.79 eV). Due to the smaller difference in the HOMO energies of nitrile imine **5a** and LUMO of dipolarophile **6**, the reaction will proceed as a normal-electron demand (NED) cycloaddition (HOMO-controlled) reaction where **5a** reacts as a nucleophile whereas **6** reacts as an electrophile. The influence of substituent nature on the energies of HOMO and LUMO of the dipole and dipolarophile is illustrated in Table 2, which shows that electron-withdrawing substituents significantly lower the energy of frontier orbitals. However, despite the decrease in the HOMO energy of nitrile imines, the interaction of HOMO **5** and LUMO **6** remains energetically more favorable for all the presented 1,3-dipoles. It means that for all cases presented in the table, a normal-electron demand cycloaddition reaction should be realized.

#### 2.2.2. Analysis of the Conceptual DFT Reactivity Indices of the Reagents

The global CDFT reactivity indices, namely the electronic chemical potential μ, the chemical hardness η, the electrophilicity ω, and the nucleophilicity *N* [45], are one of the useful tools for investigating the reactivity of the reactants as well as regioselectivity in the cycloaddition reactions [31,46,47]. The calculation of CDFT reactivity indices is the easiest way to classify the reacting molecules as electron-donating or electron-accepting, since we are interested in the direction of electron transfer at the very beginning of the 1,3-dipolar cycloaddition to understand which reaction route will be prevalent for each pair of reactants. Thus, CDFT reactivity indices at the ground state of the reagents were calculated in both gas and solution phases, which are shown in Table 3. The electronic chemical potentials (μ) of **5a** (−3.77 eV, −3.73 eV), **5j** (−4.01 eV, −3.91 eV) in both phases, and **5l** (−4,37 eV) in the solution are higher than that of **6** (−4.33 eV, −4,54 eV), indicating that in a polar 32CA reaction, the overall charge will flow from **5a, j, l**, thus acting as nucleophiles, towards **6**, acting as the electrophile. On the other hand, **5k** has an electronic chemical potential index μ of −4.58 eV in the gas phase, which is less than that of **6**, and close values of μ in the solution state −4.51 eV and −4.54 eV, respectively, which makes it difficult to define the direction of electron density flux. The results obtained for molecules in a solvent are more consistent and allow us to more confidently attribute the presented compounds to nucleophiles or electrophiles, so our conclusions are based on the values of the reactivity indices obtained taking the solvent into account.

The electrophilicity ω and nucleophilicity *N* indices for dipolarophile **6** are 1.94 (1.80) eV and 2.52 (2.77) eV, respectively, being classified as a strong electrophile and moderate nucleophile within the electrophilicity [48] and nucleophilicity scales [49]. All of the NIs have nucleophilicity indices greater than 3.00 eV and are classified as strong nucleophiles and electrophilicity indices greater than 1.5 eV as strong electrophiles, which is in agreement with the fact that NIs are Type II dipoles and can react both as electrophiles and nucleophiles [30].

Consequently, all of the NIs **could** most likely act as nucleophiles, using their HOMOs to interact with dipolarophile LUMOs, acting as electrophiles, and the process would have NED character. Although, Table 3 shows that the chemical potentials μ of dipolarophile **6** and dipole **5k** are very close to each other, in this case, FMO theory is not able to provide a reliable prediction since the two HOMO–LUMO energy gaps are comparable in size.

#### 2.2.3. Regioselectivity of Nitrile Imines Cycloaddition to Methylydene Hydantoin **6**

**Scheme 5:**
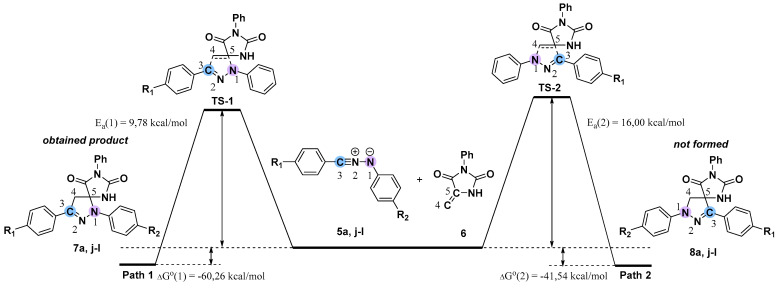
Schematic representation of minimum energy paths (MEP) corresponding to the studied 1,3-dipolar cycloaddition reaction in two pathways.

To study the regioselectivity of the 1,3-dipolar cycloaddition reaction of nitrile imines **5a, j, k,** and **l** with the exocyclic double C=C bond in the 5-th position of the hydantoin ring, two possible reaction routes were considered, in which the reactants are added together to afford two regioisomeric adducts **7** and **8** (Scheme 5). Path 1 leads to the formation of a pyrazoline adduct containing the most sterically encumbered substituent of the dipolarophile in the 5-position (C_5_) of the resulting heterocycle, which is more favorable, according to the literature [32,50], than the 4-substituted adduct, which is formed in path 2. As mentioned in the synthetic study section, the regioselective formation of the **7** adduct was observed in the 1,3-dipolar cycloaddition reaction of nitrile imines with 5-methylidenehydantoin.

In a polar reaction, when a non-symmetric nucleophile/electrophile pair react together, the most probable event is that the most nucleophilic center of the nucleophile interacts with the most electrophilic center of the electrophile. Determination of local indices requires the calculation of Fukui functions [51,52,53] in the reaction sites of reagents. In Table 4, electrophilic ($f_{k}^{-}$) and nucleophilic ($f_{k}^{+}$) Fukui functions of dipolarophiles and four nitrile imine derivatives are given. It is known that Fukui function values obtained from various population schemes may provide negative values, whereas Hirshfeld’s population scheme guarantees positive Fukui function values [53,54]. 

Analysis of the electrophilic $f_{k}^{+}$ Fukui functions at the reactive site of 5-methylidene-3-phenylhydantoin **6**, which was earlier determined to react as an electrophile, indicates that the unsubstituted C4 carbon of the double bond is the most electrophilic center of this molecule $f_{k}^{+}$(C4) = 0.179. It should be noted that C4 carbon is twice as electrophilically activated as C5 carbon $f_{k}^{+}$(C5) = 0.083.

The nucleophilic Fukui functions for each of the nitrile imines **5a, j-l** indicates that the nucleophilicity is mainly gathered at the C3 carbon and terminal N1 nitrogen. The N1 nitrogen atom is the most nucleophilic center in all the dipoles, no matter which substituent the NI **5** contains. For the compound **5k**, the Fukui nucleophilic functions for the nitrogen atom ($f_{k}^{-}$(N1) = 0.138) and carbon atom ($(f_{k}^{-}$(C3) = 0.114) of the C-N-N fragment of nitrile imine have similar values, which indicates that the considered atoms are nucleophilically activated almost equally. For the dipoles **5j** and **5l**, the difference in the activation of the N1 and C3 nucleophilic centers turns out to be insignificant, and both of them can act as nucleophiles with equal probability.

It is known that the most favorable regioisomeric reactive channel is that involving the most favorable local electrophilic and nucleophilic interactions [55]. According to the obtained results, in cycloaddition of compound **6** and dipoles **5a**, **j-l** the most favored interactions should be considered between C4 of 5-methylidene-3-phenylhydantoin (possessing the highest value of $f_{k}^{+}$) and N1 of **5a**, **j-l** (possessing the highest value of $f_{k}^{-}$), which leads to the formation of four substituted pyrazolines **8a**, **j-l**. However, in the present work, it was experimentally shown that the cycloaddition of 1,3-dipoles to methylidene hydantoin **6** always proceeded regioselectively with the formation of a 5-substituted pyrazoline rin, and that regioisomers **8a**, **j-l** were not detected in the reaction mixture. The reactions’ regioselectivity, observed in practice, may be presumably due to the fact that steric effects overcome electronic effects. Thus, it was shown that the C3 carbon atom of nitrile imines is more sensitive than N1 nitrogen to the steric requirements of the dipolarophile [31,56]. In addition, one recent study showed that Fukui functions fail in cases of molecules with concurrent electrophilic and nucleophilic activation [55]. Thus, taking into account the closeness of the values of the Fukui nucleophilic functions obtained for the C3 and N1 atoms of the C-N-N fragment of nitrile imines **5a**, **j-l**, both of which can act as nucleophiles, and also the steric hindrances of the carbon atom C5 in the exocyclic C=C bond of hydantoin **6**, compared with unsubstituted C4 carbon, we may conclude that the observed regioselectivity should be considered not only from the point of view of the electronic structure of the reagents, but also taking into account the steric factors for the reaction centers.

#### 2.2.4. Finding Transition States Using NEB-TS

In order to explain the regioselectivity of the [3+2]-cycloaddition reaction of nitrile imines **5a**, **j-l** to 5-methilidene-3-phenylhydantoin **6** we performed calculations of the minimum energy path (See Supplementary Information pp. 43–56). To find and localize the transition state, we applied the nudged elastic band method (NEB) [42]. For this purpose, the reactive channels presented in Scheme 5 were simulated, and their corresponding transition states were detected using PBE0/def2-svpd methods. Accordingly, the thermodynamic and kinetic parameters were calculated from optimized geometries of the reactants, transition states, and products. The minimum energy paths (MEP) for two possible routes of the reaction are illustrated in Scheme 5. The activation energies (E_a_(1) = 9.78 kcal/mol, E_a_(2) = 16.00 kcal/mol) corresponding to the two pathways show that path 1 is favored kinetically for the 1,3-DC reaction between nitrile imine **5a** and compound **6**. The MEPs’ analysis also indicates that the kinetic and thermodynamic product is same in studied reaction. 

**Figure 2:**
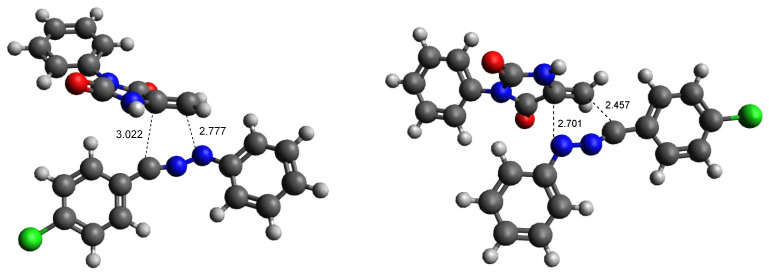
TS-I and TS-II calculated geometries.

Interestingly, the formation of product **7a** can be considered an asynchronous reaction, as the distance between atoms C3 and C4 is shorter (2.457 A) in the transition state than that for atoms N1 and C5 (2.701 A), which means that the TS could be polar (Figure 2). 

In conclusion, according to the activation energy values, the cycloaddition reaction of nitrile imine **5a** with the model dipolarophile **6** should lead to the formation of product **7a**.

Thus, the analysis of the regioselectivity of the 1,3-dipolar cycloaddition reaction of the nitrile imine **5a** to 5-methilidene-3-phenylhydantoin **6** based on calculated DFT minimum energy paths indicated the preferred regioisomer **7a** in agreement with experimental findings in the considered cases. However, the reactivity index analysis could not be used to predict the formation of regioisomer **7a,** probably because it does not take into account steric factors in the analyzed reactions. 

## 3. Materials and Methods

### 3.1. General

All solvents used were purified and dehydrated using the methods described in [57]. All starting reagents were purchased from commercial sources (Sigma-Aldrich, ABCR, AKSci, Burlington, VT, USA). Reactions were checked by TLC analysis using silica plates with a fluorescent indicator (254 nm) and visualized with a UV lamp. ^1^H and ^13^C NMR spectra were recorded on a BrukerAvance (Bruker Optik GmbH, Ettlingen, Germany) and Agilent 400-MR (Agilent Technologies, Santa Clara, CA, US) spectrometers (400 MHz for ^1^H, 100 MHz for ^13^C). Chemical shifts are reported in parts per million relative to TMS. 

Electrospray ionization high-resolution mass spectra were recorded in positive ion mode on a TripleTOF 5600+ quadrupole time-of-flight mass spectrometer (ABSciex, Concord, Vaughan, ON, Canada) equipped with a DuoSpray ion source. The following MS parameters were applied: capillary voltage 5.5 kV; nebulizing and curtain gas pressures—15 and 25 psi, respectively; ion source temperature—ambient; declustering potential 20 V; m/z range 100–1200. Elemental compositions of the detected ions were determined based on accurate masses and isotopic distributions using Formula Finder software (ABSciex, Concord, ON, Canada). The maximum allowed deviation of the experimental molecular mass from the calculated one was 5 ppm.

Experimental procedures and characteristic data for all synthesized compounds are given in the Supplementary Information [58,59,60,61,62,63,64,65,66,67,68,69,70,71].

### 3.2. Synthesis

#### 1,3-Diporal Cycloaddition of Nitrile Imines 5 with 5-Methylene-3-Phenylhydantoin **6**

General procedure: Hydrazonoyl chloride (1.1 equiv.) and 5-methylene-3-phenylhydantoin (1 equiv.) are solubilized in acetonitrile (2 mL) under an inert atmosphere, and a solution of TEA (2.2 equiv.) in acetonitrile (2 mL) is added dropwise and stirred for 30 min. After the addition, the reaction mixture is stirred for 24–48 h, when the solvent is removed in vacuo and the residue is purified by column chromatography on silica gel with EtOAc/petroleum (EA/PE) ether or MeOH/CHCl_3_ as eluent.

3-(4-Chlorophenyl)-1,8-diphenyl-1,2,6,8-tetraazaspiro [4.4]non-2-ene-7,9-dione (**7a**)

Compound **7a** was prepared from hydrazonoyl chloride **4a** (62 mg, 0.23 mmol), 5-methylene-3-phenylhydantoin (40 mg, 0.21 mmol), and TEA (0.065 mL, 0.47 mmol). Yield of 88 mg (99%). White solid. Mp of 195–196 °C. Chromatography: EA/PE, 1:10–1:6. R_f_ = 0.32 (EA/PE, 1:4).

^1^H NMR (400 Hz, DMSO-*d*_6_): δ 9.59 (s, 1H, NH), 7.79 (d, J = 8.6 Hz, 2H, Ar), 7.60–7.51 (m, 4H, Ar), 7.49–7.43 (m, 1H, Ar), 7.41–7.33 (m, 4H, Ar), 7.13 (d, J = 7.2 Hz, 2H, Ar), 7.03 (t, J = 7.3, 1.2 Hz, 1H, Ar), 3.95 (d, J = 18.3 Hz, 1H, CH_2_), 3.77 (d, J = 18.3 Hz, 1H, CH_2_). ^13^C NMR (100 MHz, DMSO-*d*_6_): δ 171.2, 153.4, 146.8, 142.4, 133.9, 131.4, 130.2, 129.4, 129.1, 129.0, 128.4, 127.6, 126.5, 122.5, 116.4, 81.5, 44.6. HRMS (ESI): calcd forC_23_H_17_ClN_4_O_2_ (M+H)^+^ 417.1113, found 417.1109.

3-(4-Bromophenyl)-1,8-diphenyl-1,2,6,8-tetraazaspiro[4.4]non-2-ene-7,9-dione (**7b**)

Compound **7b** was prepared from hydrazonoyl chloride **4b** (105 mg, 0.35 mmol), 5-methylene-3-phenylhydantoin (60 mg, 0.32 mmol), and TEA (0.097 mL, 0.70 mmol). Yield 137 mg (95%). White solid. Mp of 205–206 °C. Chromatography: EA/PE, 1:8–1:4. R_f_ = 0.28 (EA/PE, 1:4).

^1^H NMR (400 Hz, DMSO-*d*_6_): δ 9.57 (s, 1H, NH), 7.74–7.66 (m, 4H, Ar), 7.57–7.51 (m, 2H, Ar), 7.49–7.43 (m, 1H, Ar), 7.41–7.33 (m, 4H, Ar), 7.13 (d, J = 7.7 Hz, 2H, Ar), 7.03 (t, J = 7.4 Hz, 1H, Ar), 3.95 (d, J = 18.3 Hz, 1H, CH_2_), 3.77 (d, J = 18.3 Hz, 1H, CH_2_). ^13^C NMR (100 MHz, DMSO-*d*_6_): δ 171.2, 153.4, 146.8, 142.4, 131.9, 131.4, 130.6, 129.4, 129.2, 128.5, 127.9, 126.5, 122.7, 122.5, 116.5, 81.5, 44.6. HRMS (ESI): calcd for C_23_H_17_BrN_4_O_2_ (M+H)^+^ 461.0608, found 461.0601.

3-(4-Fluorophenyl)-1,8-diphenyl-1,2,6,8-tetraazaspiro[4.4]non-2-ene-7,9-dione (**7c**)

Compound **7c** was prepared from hydrazonoyl chloride **4c** (58 mg, 0.23 mmol), 5-methylene-3-phenylhydantoin (40 mg, 0.21 mmol), and TEA (0.065 mL, 0.47 mmol). Yield of 68 mg (81%). White solid. Mp of 192–194 °C. Chromatography: EA/PE, 1:8–1:4. R_f_ = 0.25 (EA/PE, 1:4).

^1^H NMR (400 Hz, DMSO-*d*_6_): δ 9.58 (s, 1H, NH), 7.86–7.79 (m, 2H, Ar), 7.58–7.50 (m, 2H, Ar), 7.49–7.43 (m, 1H, Ar), 7.40–7.30 (m, 6H, Ar), 7.13 (d, J = 7.8 Hz, 2H, Ar), 7.03 (t, J = 7.3 Hz, 1H, Ar), 3.96 (d, J = 18.2 Hz, 1H, CH_2_), 3.78 (d, J = 18.3 Hz, 1H, CH_2_). ^13^C NMR (100 MHz, DMSO-*d*_6_): δ 171.2, 164.0, 161.5, 153.4, 146.9, 142.5, 131.4, 129.3, 128.7 (d, J = 70.0 Hz), 128.1 (d, J = 8.5 Hz), 127.9 (d, J = 3.1 Hz), 126.5, 122.3, 116.4, 115.9 (d, J = 21.9 Hz), 81.5, 44.8. HRMS (ESI): calcd for C_23_H_17_FN_4_O_2_ (M+H)^+^ 401.1408, found 401.1406.

3-(3-Fluorophenyl)-1,8-diphenyl-1,2,6,8-tetraazaspiro[4.4]non-2-ene-7,9-dione (**7d**)

Compound **7d** was prepared from hydrazonoyl chloride **4d** (58 mg, 0.23 mmol), 5-methylene-3-phenylhydantoin (40 mg, 0.21 mmol), and TEA (0.065 mL, 0.47 mmol). Yield of 74 mg (88%). White solid. Mp of 198–199 °C. Recrystallized from DCM.

^1^H NMR (400 Hz, DMSO-*d*_6_): δ 9.60 (s, 1H, NH), 8.32 (s, 1H, Ar), 7.64–7.49 (m, 4H, Ar), 7.49–7.42 (m, 1H, Ar), 7.41–7.33 (m, 4H, Ar), 7.33–7.25 (m, 1H, Ar), 7.18–7.11 (m, 2H, Ar), 7.04 (t, J = 7.4 Hz, 1H, Ar), 3.96 (d, J = 18.3 Hz, 1H, CH_2_), 3.79 (d, J = 18.3 Hz, 1H, CH_2_). ^13^C NMR (100 MHz, DMSO-*d*_6_): δ 171.1, 162.3 (d, Jcf = 243.8 Hz), 153.4, 146.7, 142.3, 133.7 (d, J = 8.1 Hz), 131.4, 130.9 (d, J = 8.3 Hz), 129.3, 129.2 (d, J = 24.9 Hz), 126.5, 122.5, 122.1, 116.5, 116.2 (d, J = 21.3 Hz), 112.3 (d, J = 23.0 Hz), 81.5, 79.2, 44.6. HRMS (ESI): calcd for C_23_H_17_FN_4_O_2_ (M+H)^+^ 401.1408, found 401.1406.

3-(2,4-Dichlorophenyl)-1,8-diphenyl-1,2,6,8-tetraazaspiro[4.4]non-2-ene-7,9-dione (**7e**)

Compound **7e** was prepared from hydrazonoyl chloride **4e** (105 mg, 0.35 mmol), 5-methylene-3-phenylhydantoin (60 mg, 0.32 mmol), and TEA (0.97 mL, 0.070 mmol). Yield of 135 mg (94%). White solid. Mp of 173–175 °C. Chromatography: EA/PE, 1:10–1:6. R_f_ = 0.13 (EA/PE, 1:8).

^1^H NMR (400 Hz, DMSO-*d*_6_): δ 9.61 (s, 1H, NH), 7.87 (d, J = 8.5 Hz, 1H, Ar), 7.77 (d, J = 2.2 Hz, 1H, Ar), 7.60–7.50 (m, 3H, Ar), 7.49–7.43 (m, 1H, Ar), 7.42–7.35 (m, 4H, Ar), 7.14 (d, J = 7.7 Hz, 2H, Ar), 7.06 (t, J = 7.3, 1.1 Hz, 1H, Ar), 4.09 (d, J = 18.3 Hz, 1H, CH_2_), 3.88 (d, J = 18.3 Hz, 1H, CH_2_). ^13^C NMR (100 MHz, DMSO-*d*_6_): δ 171.56, 153.86, 145.61, 142.52, 134.64, 132.43, 131.93, 131.83, 130.84, 129.85, 129.54, 129.32, 128.89, 128.18, 127.02, 123.25, 117.13, 81.99, 47.29. HRMS (ESI): calcd for C_23_H_16_Cl_2_N_4_O_2_ (M+H)^+^ 451.0723, found 451.0721.

1,8-Diphenyl-3-(p-tolyl)-1,2,6,8-tetraazaspiro[4.4]non-2-ene-7,9-dione (**7f**)

Compound **7f** was prepared from hydrazonoyl chloride **4f** (74 mg, 0.29 mmol), 5-methylene-3-phenylhydantoin (50 mg, 0.26 mmol), and TEA (0.080 mL, 0.58 mmol). Yield of 90 mg (86%). White solid. Mp of 186–187 °C. Chromatography: EA/PE, 1:8–1:1. R_f_ = 0.30 (EA/PE, 1:4).

^1^H NMR (400 Hz, DMSO-*d*_6_): δ 9.57 (s, 1H, NH), 7.67 (d, J = 8.0 Hz, 2H, Ar), 7.54 (t, J = 7.7 Hz, 2H, Ar), 7.49–7.42 (m, 1H, Ar), 7.40–7.27 (m, 6H, Ar), 7.12 (d, J = 8.0 Hz, 2H, Ar), 7.01 (t, J = 7.3 Hz, 1H, Ar), 3.92 (d, J = 18.2 Hz, 1H, CH_2_), 3.75 (d, J = 18.2 Hz, 1H, CH_2_), 2.37 (s, 3H, CH_3_). ^13^C NMR (100 MHz, DMSO-*d*_6_): δ 171.4, 153.5, 147.8, 142.7, 139.2, 131.4, 129.5, 129.3, 129.1, 128.6, 128.4, 126.5, 125.9, 122.1, 116.2, 81.2, 45.0, 21.1. HRMS (ESI): calcd for C_24_H_20_N_4_O_2_ (M+H)^+^ 397.1659, found 397.1658.

3-(4-Methoxyphenyl)-1,8-diphenyl-1,2,6,8-tetraazaspiro[4.4]non-2-ene-7,9-dione (**7g**)

Compound **7g** was prepared from hydrazonoyl chloride **4g** (61 mg, 0.23 mmol), 5-methylene-3-phenylhydantoin (40 mg, 0.21 mmol), and TEA (0.065 mL, 0.47 mmol). Yield of 70 mg (81%). White solid. Mp of 184–186 °C. Chromatography: CHCl_3_–MeOH/CHCl_3_, 1:100. R_f_ = 0.20 (CHCl_3_).

^1^H NMR (400 Hz, DMSO-*d*_6_): δ 9.57 (s, 1H, NH), 7.73 (d, J = 8.7 Hz, 2H, Ar), 7.58–7.51 (m, 2H, Ar), 7.49–7.43 (m, 1H, Ar), 7.42–7.31 (m, 4H, Ar), 7.13 (d, J = 7.6 Hz, 2H, Ar), 7.06 (d, J = 8.8 Hz, 2H, Ar), 7.01 (t, J = 7.4 Hz, 1H, Ar), 3.93 (d, J = 18.1 Hz, 1H, CH_2_), 3.83 (s, 3H, CH_3_), 3.76 (d, J = 18.1 Hz, 1H, CH_2_). ^13^C NMR (100 MHz, DMSO-*d*_6_): δ 171.9, 160.8, 153.9, 148.1, 143.3, 131.9, 129.7, 129.5, 128.8, 128.0, 127.0, 124.3, 122.4, 116.6, 114.8, 81.6, 55.8, 45.5. HRMS (ESI): calcd for C_24_H_20_N_4_O_3_ (M+H)^+^ 413.1608, found 413.1613.

1-(4-Methoxyphenyl)-3,8-diphenyl-1,2,6,8-tetraazaspiro[4.4]non-2-ene-7,9-dione (**7h**)

Compound **7h** was prepared from hydrazonoyl chloride **4h** (84 mg, 0.32 mmol), 5-methylene-3-phenylhydantoin (55 mg, 0.29 mmol), and TEA (0.089 mL, 0.64 mmol). Yield of 88 mg (73%). White solid. Mp of 191–193 °C. Chromatography: CHCl_3_– MeOH/CHCl_3_, 1:100. R_f_ = 0.23 (CHCl_3_).

^1^H NMR (400 Hz, DMSO-*d*_6_): δ 9.52 (s, 1H, NH), 7.81–7.69 (m, 2H, Ar), 7.57–7.39 (m, 6H, Ar), 7.35–7.25 (m, 2H, Ar), 7.09 (d, J = 9.0 Hz, 2H, Ar), 6.97 (d, J = 9.1 Hz, 2H, Ar), 3.87 (d, J = 17.9 Hz, 1H, CH_2_), 3.75 (s, 3H, CH_3_), 3.72(d, J = 17.9 Hz, 2H, CH_2_). ^13^C NMR (100 MHz, DMSO-*d*_6_): δ 171.6, 156.0, 153.7, 147.8, 136.2, 131.8, 131.7, 129.5, 129.3, 129.1, 128.6, 126.8, 126.0, 120.5, 114.7, 82.8, 55.5, 44.1. HRMS (ESI): calcd for C_24_H_20_N_4_O_3_ (M+H)^+^ 413.1608, found 413.1611.

1,8-Diphenyl-3-(3,4,5-trimethoxyphenyl)-1,2,6,8-tetraazaspiro[4.4]non-2-ene-7,9-dione (**7i**)

Compound **7i** was prepared from hydrazonoyl chloride **4i** (56 mg, 0.18 mmol), 5-methylene-3-phenylhydantoin (30 mg, 0.16 mmol), and TEA (0.049 mL, 0.35 mmol). Yield of 64 mg (85%). White solid. Mp of 206–207 °C. Chromatography: MeOH/CHCl_3_, 1:100–1:50. R_f_ = 0.10 (EA/PE, 1:4).

^1^H NMR (400 Hz, DMSO-*d*_6_): δ 9.62 (s, 1H, NH), 7.55 (t, J = 7.6 Hz, 2H, Ar), 7.50–7.42 (m, 1H, Ar), 7.41–7.33 (m, 4H, Ar), 7.14 (d, J = 8.0 Hz, 2H, Ar), 7.08–6.97 (m, 3H, Ar), 3.99 (d, J = 18.3 Hz, 1H, CH_2_), 3.86 (s, 6H, m-OCH_3_), 3.82 (d, J = 18.7 Hz, 1H, CH_2_), 3.71 (s, 3H, p-OCH_3_). ^13^C NMR (100 MHz, DMSO-*d*_6_): δ 171.3, 153.4, 153.1, 147.8, 142.6, 138.8, 131.4, 129.4, 129.2, 128.5, 126.8, 126.6, 122.2, 116.2, 103.4, 81.3, 60.2, 56.0, 45.2. HRMS (ESI): calcd for C_26_H_24_N_4_O_5_ (M+H)^+^ 473.1820, found 473.1812.

1,8-Diphenyl-3-(4-(trifluoromethyl)phenyl)-1,2,6,8-tetraazaspiro[4.4]non-2-ene-7,9-dione (**7j**)

Compound **7j** was prepared from hydrazonoyl chloride **4j** (222 mg, 0.74 mmol), 5-methylene-3-phenylhydantoin (70 mg, 0.37 mmol), and TEA (0.207 mL, 1.49 mmol). Yield of 100 mg (60%). White solid. Mp of 189–191 °C. Chromatography: EA/PE, 1:12–1:6. R_f_ = 0.35 (EA/PE, 1:4).

^1^H NMR (400 Hz, DMSO-*d*_6_): δ 9.64 (s, 1H, NH), 7.97 (d, J = 8.1 Hz, 2H, Ar), 7.85 (d, J = 8.2 Hz, 2H, Ar), 7.59–7.51 (m, 2H, Ar), 7.49–7.43 (m, 1H, Ar), 7.43–7.35 (m, 4H, Ar), 7.17 (d, J = 7.9 Hz, 2H, Ar), 7.06 (t, J = 7.3 Hz, 1H, Ar), 4.02 (d, J = 18.3 Hz, 1H, CH_2_), 3.83 (d, J = 18.3 Hz, 1H, CH_2_). ^13^C NMR (100 MHz, DMSO-*d*_6_): δ 171.1, 153.4, 146.4, 142.1, 135.2, 131.4, 129.4, 129.1, 129.2 (q, J = 31 Hz), 128.4, 128.2, 126.5, 126.5, 125.8 (q, J = 3.8 Hz), 124.6 (q, Jcf = 272 Hz), 122.8, 116.6, 81.6, 44.4. HRMS (ESI): calcd for C_24_H_17_F_3_N_4_O_2_ (M+H)^+^ 451.1376, found 451.1368.

3-(4-Nitrophenyl)-1,8-diphenyl-1,2,6,8-tetraazaspiro[4.4]non-2-ene-7,9-dione (**7k**)

Compound **7k** was prepared from hydrazonoyl chloride **4k** (79 mg, 0.26 mmol), 5-methylene-3-phenylhydantoin (45 mg, 0.24 mmol), and TEA (0.073 mL, 0.53 mmol). Yield of 90 mg (83%). Yellow solid. Mp of 231–233 °C. Chromatography: EA/PE, 1:10–1:4. R_f_ = 0.15 (EA/PE, 1:4).

^1^H NMR (400 Hz, DMSO-*d*_6_): δ 9.64 (s, 1H, NH), 8.33 (d, J = 8.8 Hz, 2H, Ar), 8.00 (d, J = 8.6 Hz, 2H, Ar), 7.58–7.51 (m, 2H, Ar), 7.50–7.37 (m, 5H, Ar), 7.18 (d, J = 8.0 Hz, 2H, Ar), 7.08 (t, J = 7.3 Hz, 1H, Ar), 4.03 (d, J = 18.3 Hz, 1H, CH_2_), 3.85 (d, J = 18.4 Hz, 1H, CH_2_).^13^C NMR (100 MHz, DMSO-*d*_6_): δ 170.9, 153.4, 147.3, 145.9, 141.8, 137.5, 131.4, 129.5, 129.1, 128.5, 126.8, 126.5, 124.2, 123.1, 116.8, 81.8, 44.2. HRMS (ESI): calcd for C_23_H_17_N_5_O_4_ (M+H)_+_ 428.1353, found 428.1350.

1-(4-Nitrophenyl)-3,8-diphenyl-1,2,6,8-tetraazaspiro[4.4]non-2-ene-7,9-dione (**7l**) and 1-(4-Nitrophenyl)-N,3-diphenyl-1H-pyrazole-5-carboxamide (**9a**)

Compounds **7l** and **9a** were prepared from hydrazonoyl chloride **4l** (129 mg, 0.47 mmol), 5-methylene-3-phenylhydantoin (80 mg, 0.43 mmol), and TEA (0.130 mL, 0.94 mmol) and separated by a column chromatography on silica gel using EtOAc/petroleum (1:10–1:4).

The yield of compound **7l** was 66 mg (36%). Yellow solid. Mp of 198–200 °C. R_f_ = 0.35 (EA/PE, 1:4).

^1^H NMR (400 Hz, DMSO-*d*_6_): δ 9.69 (s, 1H, NH), 8.30 (d, J = 9.4 Hz, 2H, Ar), 7.88–7.80 (m, 2H, Ar), 7.61–7.45 (m, 8H, Ar), 7.24 (d, J = 9.4 Hz, 2H, Ar), 4.15 (d, J = 18.8 Hz, 1H, CH_2_), 3.93 (d, J = 18.8 Hz, 1H, CH_2_). ^13^C NMR (100 MHz, DMSO-*d*_6_): δ 170.4, 153.3, 150.9, 147.1, 140.2, 131.2, 130.5, 130.3, 129.1, 129.0, 128.7, 126.9, 126.4, 126.1, 113.3, 79.9, 46.0. HRMS (ESI): calcd for C_23_H_17_N_5_O_4_ (M+H)^+^ 428.1353, found 428.1352.

The yield of compound **9a** was 28 mg (23%). Red solid. Mp 2of 50–251 °C. Rf = 0.6 (EA/PE, 1:4).

^1^H NMR (400 Hz, DMSO-*d*_6_): δ 10.82 (s, 1H, NH), 8.36 (dt, *J* = 9.2, 2.9 Hz, 2H, Ar), 7.99–7.92 (m, 2H, Ar), 7.85 (dt, *J* = 9.0, 2.2 Hz, 2H, Ar), 7.72–7.67 (m, 2H, Ar), 7.67 (s, 1H, CH),7.54–7.46 (m, 2H, Ar), 7.43 (tt, *J* = 7.5, 1.4 Hz, 1H, Ar), 7.39–7.31 (m, 2H, Ar), 7.14 (tt, *J* = 7.7, 1.2 Hz, 1H, Ar). ^13^C NMR (100 MHz, DMSO-*d*_6_): δ 158.1, 152.1, 146.6, 144.9, 139.6, 138.7, 131.9, 129.5, 129.3, 129.3, 126.0, 125.2, 125.0, 124.8, 120.6, 108.8. HRMS (ESI): calcd for C_22_H_16_N_4_O_3_ (M+H)^+^ 385.1295, found 385.1299.

3-(2-Chloro-5-nitrophenyl)-1,8-diphenyl-1,2,6,8-tetraazaspiro[4.4]non-2-ene-7,9-dione (**7m**)

Compound **7m** was prepared from hydrazonoyl chloride **4m** (85 mg, 0.27 mmol), 5-methylene-3-phenylhydantoin (47 mg, 0.25 mmol), and TEA (0.076 mL, 0.55 mmol). Yield of 40 mg (35%). Yellow solid. Mp of 196–198 °C. Chromatography: EA/PE, 1:8–1:2. R_f_ = 0.31 (EA/PE, 1:4)

^1^H NMR (400 Hz, DMSO-*d*_6_): δ 9.63 (s, 1H, NH), 8.57 (d, J = 2.7 Hz, 1H, Ar), 8.26 (dd, J = 8.8, 2.8 Hz, 1H, Ar), 7.90 (d, J = 8.9 Hz, 1H, Ar), 7.55 (t, J = 7.7 Hz, 2H, Ar), 7.49–7.36 (m, 5H, Ar), 7.16 (d, J = 7.9 Hz, 2H, Ar), 7.09 (t, J = 7.4 Hz, 1H, Ar), 4.18 (d, J = 18.3 Hz, 1H, CH_2_), 3.96 (d, J = 18.4 Hz, 1H, CH_2_). ^13^C NMR (100 MHz, DMSO-*d*_6_): δ 171.3, 153.8, 146.9, 145.1, 142.2, 138.0, 133.0, 131.8, 131.7, 129.9, 129.5, 128.9, 127.0, 125.0, 123.6, 117.4, 82.3, 47.0. HRMS (ESI): calcd for C_23_H_16_ClN_5_O_4_ (M+H)^+^ 462.0964, found 462.0966.

4-(7,9-Dioxo-1,8-diphenyl-1,2,6,8-tetraazaspiro[4.4]non-2-en-3-yl)benzonitrile (**7n**)

Compound **7n** was prepared from hydrazonoyl chloride **4n** (90 mg, 0.35 mmol), 5-methylene-3-phenylhydantoin (60 mg, 0.32 mmol), and TEA (0.097 mL, 0.70 mmol). Yield of 42 mg (32%). White solid. Mp of 243–244 °C. Chromatography: EA/PE, 1:10–1:4. R_f_ = 0.3 (EA/PE, 1:4).

^1^H NMR (400 Hz, DMSO-*d*_6_): δ 9.63 (s, 1H, NH), 7.98–7.90 (m, 4H, Ar), 7.58–7.52 (m, 2H, Ar), 7.49–7.43 (m, 1H, Ar), 7.43–7.35 (m, 4H, Ar), 7.17 (d, J = 7.6 Hz, 2H, Ar), 7.07 (t, J = 7.3 Hz, 1H, Ar), 4.00 (d, J = 18.1 Hz, 1H, CH_2_), 3.82 (d, J = 18.4 Hz, 1H, CH_2_).^13^C NMR (100 MHz, DMSO-*d*_6_): δ 171.0, 153.4, 146.2, 142.0, 135.6, 132.8, 131.4, 129.5, 129.2, 128.5, 126.5, 126.5, 122.9, 118.8, 116.7, 111.2, 81.7, 44.2. HRMS (ESI): calcd for C_24_H_17_N_5_O_2_ (M+H)^+^ 408.1455, found 408.1459.

1-(2,4-Dinitrophenyl)-3-(4-methoxyphenyl)-8-phenyl-1,2,6,8-tetraazaspiro[4.4]non-2-ene-7,9-dione (**7o**)

Compound **7o** was not obtained in the reactions of hydrazonoyl chloride **4o** (144 mg, 0.41 mmol), 5-methylene-3-phenylhydantoin (70 mg, 0.37 mmol), and TEA (0.114 mL, 0.82 mmol). As a result of the reaction, a complex mixture of different products was obtained.

3-Methyl-1,8-diphenyl-1,2,6,8-tetraazaspiro[4.4]non-2-ene-7,9-dione (**7p**)

Compound **7p** was prepared from hydrazonoyl chloride **4p** (19 mg, 0.11 mmol), 5-methylene-3-phenylhydantoin (19 mg, 0.10 mmol), and TEA (0.031 mL, 0.22 mmol). Yield of 20 mg (63%). Orange solid. Mp of 85–87 °C. Chromatography: MeOH/CHCl_3_, 1:100–1:50. R_f_ = 0.37 (MeOH/CHCl_3_, 1:4).

^1^H NMR (400 Hz, CDCl_3_): δ 7.48–7.41 (m, 2H, Ar), 7.40–7.32 (m, 1H, Ar), 7.30–7.21 (m, 4H, Ar), 7.12–7.06 (m, 2H, Ar), 7.06–7.030(m, 1H, Ar), 6.83 (bs, 1H), 3.54 (dd, J–17.9, 1.4 Hz, 1H, CH_2_), 3.01 (d, J–17.9 Hz, 1H, CH_2_), 2.02 (s, 3H, CH_3_). ^13^C NMR (100 MHz, CDCl_3_): δ 171.1, 154.2, 148.9, 142.9, 130.9, 129.3, 129.3, 128.6, 125.9, 123.6, 118.1, 81.9, 48.6, 15.5. HRMS (ESI): calcd for C_18_H_16_N_4_O_2_ (M+H)^+^ 321.1346, found 321.1355.

## 4. Conclusions

A series of new spiro derivatives containing pyrazoline and hydantoin fragments was synthesized by 1,3-dipolar cycloaddition of nitrile imines, which were generated in situ from hydrazonyl chlorides, to the 3-phenyl-5-methylidenehydantoin. It was shown that the cycloaddition reactions proceed regioselectively in all cases, regardless of the presence of electron-donating or electron-withdrawing substituents in the 1,3-dipole molecules. The introduction of electron-withdrawing substituents into the aromatic ring both at the C3 carbon atom and at the N1 nitrogen atom of the C-N-N fragment of nitrile imine leads, in most cases, to a decrease in the yields of the reaction product due to a decrease in the HOMO energy of the 1,3-dipole.

The study of the 1,3-dipolar cycloaddition reaction of nitrile imines by DFT calculation methods showed that 1,3-dipoles act as nucleophiles in the reaction with 3-phenyl-5-methylidenehydantoin, and the 1,3-dipolar cycloaddition reaction with normal electronic demands (NED) should have been realized. In this case, taking into account the values of the Fukui functions obtained for the reaction centers of the dipole and dipolarophile, the interaction of the HOMO of nitrile imine with the LUMO of the dipolarophile should be accompanied by the formation of a 4-substituted pyrazoline ring. However, when calculating Fukui’s nucleophilicity and electrophilicity functions, the steric factor is not taken into account, which, apparently, has a decisive influence on the result of cycloaddition. The values of CDFT reactivity indices and FMO interaction energy gaps also show that the considered processes have NED characteristics. In contrast, the results obtained in the calculation of transition states and the reaction path of the 1,3-dipolar cycloaddition of nitrile imines to 5-methylidene-3-phenylhydantoin are consistent with experimental data and predict the formation of a thermodynamically and kinetically favorable product, 1,2,6,8-tetraazaspiro [4.4]non-2-ene-7,9-dione (1,5-disubstituted pyrazoline).

## Figures and Tables

**Table 1:** Optimization of reaction conditions (for the hydrazonoyl chloride **4a** interaction with 5-methylene-3-phenylhydantoin **6**).

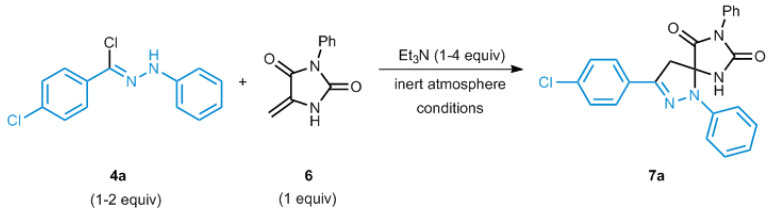						
Entry	4a, Equiv.	Et_3_N, Equiv.	Solvent	T, °C	Time, h	Yield ^a^ (%)
1	1	1	CH_2_Cl_2_	22	24	81
2	1,1	2,2	CH_2_Cl_2_	22	24	88
3	2	4	CH_2_Cl_2_	22	48	58
4	1,1	2,2	CH_3_CN	22	48	99
5	1,1	2,2	PhH	60	12	82
6	1,1	2,2	PhMe	110	12	44
7	1,1	2,2	EtOAc	22	48	74
8	1,1	2,2	CHCl_3_	22	24	74

^a^ Isolated yield.

**Table 2:** Calculated HOMO–LUMO gaps for nitrile imines **5a, j-l**, and dipolarophile **6**.

	LUMO, eV	HOMO, eV	LUMO_6_–HOMO_5_, eV	LUMO_5_–HOMO_6_, eV
**6**	−1.88	−7.20	-	-
**5a** (R_1_ = Cl)	−1.79	−5.67	3.79	5.41
**5j** (R_1_ = CF_3_)	−2.03	−5.79	3.90	5.17
**5k** (R_1_ = NO_2_)	−3.09	−5.93	4.05	4.11
**5l** (R_2_ = NO_2_)	−2.64	−6.11	4.22	4.56

**Table 3:** The PBE0/def2-svpd calculated FMO energies: electronic chemical potential (μ), chemical hardness (η), electrophilicity (ω), and nucleophilicity (N), in eV, for nitrile imines **5a, j-l**, and 5-methilidene-3-phenylhydantoin 6.

Compound	R_1_	R_2_	HOMO	LUMO	μ	η	ω	*N*
**6** (gas)	-	-	−6.94	−1.72	−4.33	5.21	1.80	2.77
**5a** (gas)	Cl	H	−5.63	−1.90	−3.77	3.74	1.90	4.08
**5j** (gas)	CF_3_	H	−5.83	−2.20	−4.01	3.63	2.22	3.98
**5k** (gas)	NO_2_	H	−6.07	−3.09	−4.58	2.98	3.52	3.64
**5l** (gas)	H	NO_2_	−6.19	−2.51	−4.35	3.68	2.57	3.52
**6** (CH_2_Cl_2_)	-	-	−7.20	−1.88	−4.54	5.32	1.94	2.52
**5a** (CH_2_Cl_2_)	Cl	H	−5.67	−1.79	−3.73	3.88	1.80	3.69
**5j** (CH_2_Cl_2_)	CF_3_	H	−5.79	−2.03	−3.91	3.75	2.04	3.93
**5k** (CH_2_Cl_2_)	NO_2_	H	−5.93	−3.09	−4.51	2.85	3.57	3.79
**5l** (CH_2_Cl_2_)	H	NO_2_	−6.11	−2.64	−4.37	3.47	2.76	3.61

**Table 4:** Calculated Fukui functions and local electrophilicity (ω_k_) and nucleophilicity (N_k_) at the reactive sites of nitrile imines **5a**, **j-l**, and 5-methylidenehydantoin **6**.

		Gas		CH_2_Cl_2_			
Entry		$f_{k}^{+}$	$f_{k}^{-}$	$f_{k}^{+}$	$f_{k}^{-}$	ω_k_	N_k_
**6**	C5	0.074	0.024	0.083	0.095	0.16	0.24
	C4	0.157	0.084	0.179	0.178	0.35	0.45
**5a**	N1	0.065	0.123	0.064	0.155	0.11	0.63
	N2	0.059	0.028	0.066	0.044	0.12	0.18
	C3	0.037	0.105	0.060	0.111	0.11	0.45
**5j**	N1	0.068	0.120	0.067	0.151	0.14	0.59
	N2	0.056	0.033	0.064	0.045	0.13	0.18
	C3	0.039	0.111	0.054	0.116	0.11	0.46
**5k**	N1	0.067	0.112	0.053	0.138	0.19	0.52
	N2	0.044	0.029	0.035	0.043	0.12	0.16
	C3	0.014	0.107	0.021	0.114	0.07	0.43
**5l**	N1	0.031	0.130	0.023	0.155	0.06	0.56
	N2	0.044	0.028	0.027	0.041	0.07	0.15
	C3	0.051	0.100	0.052	0.098	0.14	0.35

## Data Availability

The data are available from the authors upon request.

## Supplementary Materials

The following supporting information can be downloaded at: https://www.mdpi.com/article/10.3390/ijms24021289/s1, experimental data.
