# Supplementary material for: Regioselective Cycloaddition of Nitrile Imines to 5-Methylidene-3-phenyl-hydantoin: Synthesis and DFT Calculations
Source: Int J Mol Sci. 2023 Jan 9;24(2):1289. doi: 10.3390/ijms24021289 (PMC9864863; doi:10.3390/ijms24021289)
Supplement: Supplementary file 1 [file ijms-24-01289-s001.zip › ijms-2134060-supplementary.pdf]

Regioselective cycloaddition of nitrile imines to 5-methylidene-3-phenyl-hydantoin: synthesis and DFT calculations

Maria E. Filkina [1], Daria N. Baray [1], Elena K. Beloglazkina [1,*], Yuri K. Grishin [1], Vitaly A. Roznyatovsky [1] and Maxim E. Kukushkin [1,*]

[1] Department of Chemistry, M.V. Lomonosov Moscow State University Leninskie Gory, 1-3, 119991 Moscow, Russian Federation

Supplementary Information

Table of contents

Experimental section 2
General remarks 2
Synthesis of benzohydrazides 3 2
Synthesis of hydrazonoyl chlorides 4 7
1,3-Diporal cycloaddition of nitrile imines with 5-methylene-3-phenylhydantoin 6 13
Characterization data of obtained spiro products 21
DFT calculations 45
References 58

Experimental section

General remarks

All solvents used were purified and dehydrated using the methods described in [1]. All starting reagents were purchased from commercial sources (Sigma-Aldrich, ABCR, AKSci, Burlington, VT, USA). Reactions were checked by TLC analysis using silica plates with a fluorescent indicator (254 nm) and visualized with a UV lamp. ^{1}H and ^{13}C NMR spectra were recorded on a BrukerAvance and Agilent 400-MR spectrometers (400 MHz for ^{1}H, 100 MHz for ^{13}C). Chemical shifts are reported in parts per million relative to TMS.

Electrospray ionization high-resolution mass spectra were recorded in positive ion mode on a TripleTOF 5600+ quadrupole time-of-flight mass spectrometer (ABSciex, Concord, Vaughan, ON, Canada) equipped with DuoSpray ion source. The following MS parameters were applied: capillary voltage 5.5 kV; nebulizing and curtain gas pressure—15 and 25 psi, respectively; ion source temperature—ambient; declustering potential 20 V; *m/z* range 100–1200. Elemental compositions of the detected ions were determined based on accurate masses and isotopic distributions using Formula Finder software (ABSciex, Concord, ON, Canada). The maximum allowed deviation of the experimental molecular mass from the calculated one was 5 ppm.

Synthesis of benzohydrazides 3 [2]

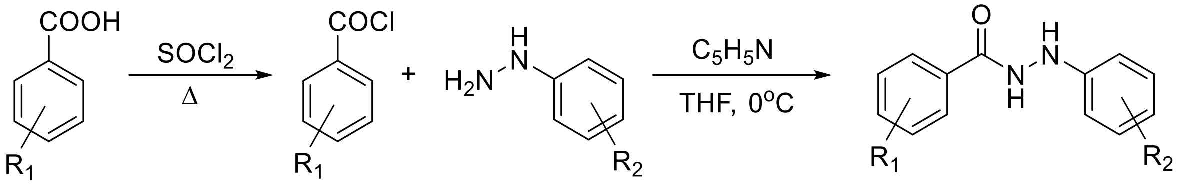

General procedure. Benzoic acid (10 mmol) was dissolved in $SOCl_2$ (12.5 mmol) in a 100 mL round-bottomed flask. The mixture was heated for 2 hours at 100°C (5 mL $SOCl_2$ was added after 20 min to make a clear solution), cooled and then distilled to remove excess $SOCl_2$. Phenylhydrazine (1.1equiv.) was dissolved in pyridine (2.2 equiv.) in a 50 mL flask and cooled at 5°C with an ice bath. In parallel, benzoyl chloride (1 equiv.) was dissolved in 5 mL of THF. The acyl chloride solution was then added dropwise at 5°C to the phenylhydrazine solution. After addition, the temperature was allowed to warm to room temperature and the medium was stirred during 2 hours. Pyridinium chloride gradually appeared in the medium. After completion of the reaction, water (30 mL) is added, pyridinium chloride dissolved and benzoyl phenylhydrazine precipitated. The precipitate was filtered, washed by diethylether (3x10 mL) and dried.

4-Chloro-N'-phenylbenzohydrazide (3a)

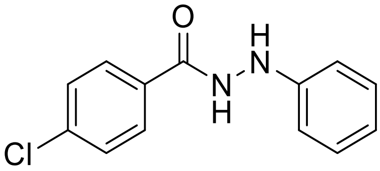

Compound **3a** was prepared according to the general procedure from phenylhydrazine (2.03 mL, 20.6 mmol) which was solubilized in pyridine (3.33 mL, 41.3 mmol) and 4-chlorobenzoyl chloride (3.28 g, 18.8 mmol) which was dissolved in 5 mL in THF. Yield 3.88 g (84%). White solid.

^{1}H NMR (400 Hz, DMSO-d_6): δ 10.45 (d, J = 2.9 Hz, 1H, NH), 7.93 (d, J = 8.5 Hz, 2H, Ar), 7.59 (d, J = 8.4 Hz, 2H, Ar), 7.15 (t, J = 7.3 Hz, 2H, Ar), 6.78 (d, J = 7.7 Hz, 2H, Ar), 6.72 (t, J = 7.3 Hz, 1H, Ar). Mp 140-142 °C (lit. 140.5-141.6 °C [3])

4-Bromo-N'-phenylbenzohydrazide (3b)

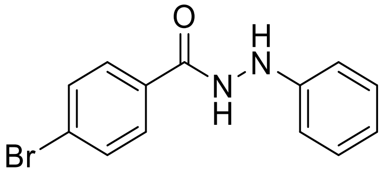

Compound **3b** was prepared according to the general procedure from phenylhydrazine (1.02 mL, 10.4 mmol) which was solubilized in pyridine (1.67 mL, 20.8 mmol) and 4-bromobenzoyl chloride (2.07 g, 9.4 mmol) which was dissolved in 5 mL in THF. Yield 2.34 g (77%). White solid.

^{1}H NMR (400 Hz, DMSO-d_6): δ 10.45 (s, 1H, NH), 7.95 (s, 1H, NH), 7.86 (d, J = 8.7 Hz, 2H, Ar), 7.73 (d, J = 8.7 Hz, 2H, Ar), 7.15 (t, J = 8.2 Hz, 2H, Ar), 6.78 (d, J = 7.7 Hz, 2H, Ar), 6.72 (tt, J = 7.3, 1.1 Hz, 1H, Ar). Mp 195-196 °C (lit. 196-198 °C [4])

4-Fluoro-N'-phenylbenzohydrazide (3c)

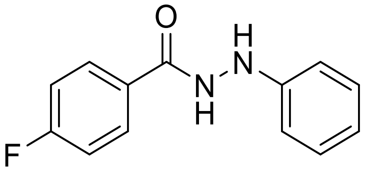

Compound **3c** was prepared according to the general procedure from phenylhydrazine (0.32 mL, 3.2 mmol) which was solubilized in pyridine (0.52 mL, 6.4 mmol) and 4-fluorobenzoyl chloride (0.46 g, 2.9 mmol) which was dissolved in 5 mL in THF. Yield 0.59 g (88%). White solid.

^{1}H NMR (400 Hz, DMSO-d_6): δ 10.39 (s, 1H, NH), 8.04 – 7.96 (m, 2H, Ar), 7.92 (s, 1H, NH), 7.35 (t, J = 9.1 Hz,2H, Ar), 7.19 – 7.12 (m, 2H, Ar), 6.78 (d, J = 7.8 Hz, 2H, Ar), 6.72 (t, J = 7.7 Hz, 1H, Ar). Mp 170.5-172 °C (lit. 171-173 °C [4])

3-Fluoro-N'-phenylbenzohydrazide (3d)

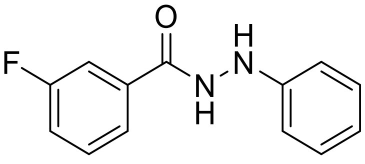

Compound **3d** was prepared according to the general procedure from phenylhydrazine (1.02 mL, 10.3 mmol) which was solubilized in pyridine (1.66 mL, 20.6 mmol) and 3-fluorobenzoyl chloride (1.49 g, 9.4 mmol) which was dissolved in 5 mL in THF. Yield 1.23 g (57%). White solid.

^{1}H NMR (400 Hz, DMSO-d_6): δ 10.46 (d, *J* = 2.5 Hz, 1H, NH), 7.95 (d, *J* = 3.0 Hz, 1H, NH), 7.77 (d, J = 7.8 Hz, 1H, Ar), 7.71 (ddd, J = 9.9, 2.7, 1.5 Hz, 1H, Ar), 7.57 (td, J = 8.0, 5.8 Hz, 1H, Ar), 7.44 (td, J = 8.4, 2.7, 1.0 Hz, 1H, Ar), 7.19 – 7.10 (m, 2H, Ar), 6.78 (d, J = 7.7 Hz, 2H, Ar), 6.72 (t, J = 7.3 Hz, 1H, Ar). Mp 119-122 °C (lit. 120-122 °C [4])

2,4-Dichloro-N'-phenylbenzohydrazide (3e)

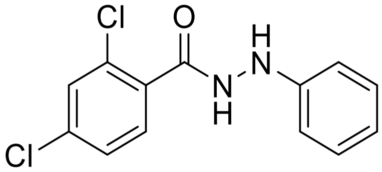

Compound **3e** was prepared according to the general procedure from phenylhydrazine (1.16 mL, 11.8 mmol) which was solubilized in pyridine (0.95 mL, 11.8 mmol) and 2,4-dichlorobenzoyl chloride (2.46 g, 11.8 mmol) which was dissolved in 5 mL in THF. Yield 1.97 g (60%). White solid.

^{1}H NMR (400 Hz, DMSO-*d$_6$*): δ 10.28 (s, 1H, NH), 7.76 (s, 1H, NH), 7.62 – 7.52 (m, 2H, Ar), 7.18 (t, J = 7.4 Hz, 2H, Ar), 6.85 (d, J = 7.7 Hz, 2H, Ar), 6.74 (tt, J = 7.2, 1.1 Hz, 1H, Ar). Mp 170-171 °C (lit. 169.1 °C [5])

4-Methyl-N'-phenylbenzohydrazide (3f)

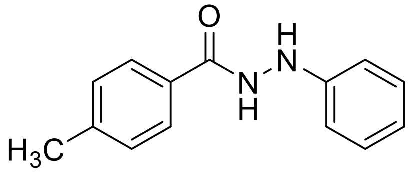

Compound **3f** was prepared according to the general procedure from phenylhydrazine (1.13 mL, 11.5 mmol) which was solubilized in pyridine (1.85 mL, 23.0 mmol) and 4-methylbenzoyl chloride (1.62 g, 10.4 mmol) which was dissolved in 5 mL in THF. Yield 1.18 g (50%). White solid.

^{1}H NMR (400 Hz, DMSO-*d$_6$*): δ 10.29 (s, 1 H, NH), 7.83 (d, J=8.15 Hz, 2H, Ar), 7.31 (d, J=7.94 Hz, 2 H, Ar), 7.09 - 7.20 (m, 2H, Ar), 6.75 - 6.82 (m, 2H, Ar), 6.68 - 6.75 (m, 1H, Ar), 2.37 (s, 3H, CH_3). Mp 166-168 °C (lit. 166-167 °C [4])

4-Methoxy-N'-phenylbenzohydrazide (3g)

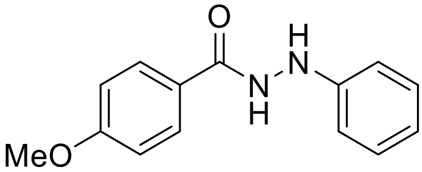

Compound **3g** was prepared according to the general procedure from phenylhydrazine (1.40 mL, 14.2 mmol) which was solubilized in pyridine (2.28 mL, 28.3 mmol) and 4-methoxylbenzoyl chloride (2.20 g, 12.9 mmol) which was dissolved in 5 mL in THF. Yield 2,44 g (78%). White solid.

^{1}H NMR (400 Hz, DMSO-*d$_6$*): δ 10.24 (s, 1H, NH), 7.96 – 7.88 (m, 2H, Ar), 7.14 (t, J = 7.3 Hz, 2H, Ar), 7.07 – 6.99 (m, 2H, Ar), 6.82 – 6.74 (m, 2H, Ar), 6.73 – 6.66 (m, 1H, Ar), 3.82 (s, 3H, OCH_3). Mp 163-164 °C (lit. 165-168 °C [6])

N'-(4-Methoxyphenyl)benzohydrazide (3h)

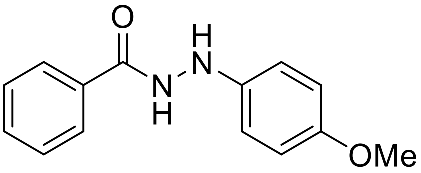

Compound **3h** was prepared according to the general procedure from (4-methoxyphenyl)hydrazine hydrochloride (0.41 g, 2.3 mmol) which was solubilized in pyridine (0.38 mL, 4.6 mmol) and benzoyl chloride (0.26mL, 2.9 mmol) which was dissolved in 5 mL in THF. Yield 0.26 g (51%). White solid.

^{1}H NMR (400 Hz, DMSO-*d$_6$*): δ 10.36 (d, *J* = 3.6 Hz, 1H, NH), 7.94 – 7.88 (m, 2H, Ar), 7.61 – 7.47 (m, 1H, Ar), 7.53 – 7.47 (m, 2H, Ar), 6.81 – 6.74 (m, 4H, Ar), 3.67 (s, 3H, OCH_3). Mp 138-140 °C (lit. 139-140 °C [7])

3,4,5-Trimethoxy-N'-phenylbenzohydrazide (3i)

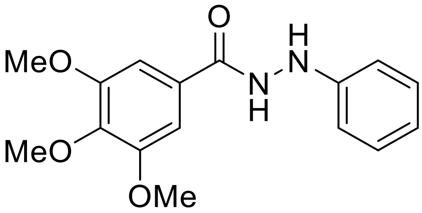

Compound **3i** was prepared according to the general procedure from phenylhydrazine (1.04 mL, 10.6 mmol) which was solubilized in Et_3N (1.47 mL, 10.6 mmol) and 3,4,5-trimethoxybenzoyl chloride (2.44 g, 10.6 mmol) which was dissolved in 5 mL in THF. Yield 2.67 g (83%). Yellow solid.

^{1}H NMR (400 Hz, DMSO-*d$_6$*): δ 10.64 (s, 1H, NH), 9.48 (s, 1H, NH), 7.06 (s, 1H, Ar), 6.23 (s, 1H, Ar), 6.05 (s, 2H, Ar), 6.00 – 5.89 (m, 1H, Ar), 5.90 – 5.78 (m, 2H, Ar), 3.02 – 2.91 (m, 9H, OCH_3). Mp 121-125 °C

N'-Phenyl-4-(trifluoromethyl)benzohydrazide (3j)

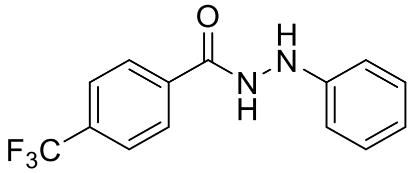

Compound **3j** was prepared according to the general procedure from phenylhydrazine (0.90 mL, 9.1 mmol) which was solubilized in pyridine (1.47 mL, 18.2 mmol) and 4-(trifluoromethyl)benzoyl chloride (1.73 g, 8.3 mmol) which was dissolved in 5 mL in THF. Yield 1.34 g (58%). White solid.

^{1}H NMR (400 Hz, DMSO-*d$_6$*): δ 10.61 (d, J = 3.1 Hz, 1H, NH), 8.12 (d, J = 8.0 Hz, 2H, Ar), 8.03 (d, J = 2.9 Hz, 1H, NH), 7.90 (d, J = 8.0 Hz, 2H, Ar), 7.16 (t, J = 7.9 Hz, 2H, Ar), 6.81 (d, J = 7.9 Hz, 2H, Ar), 6.73 (t, J = 7.3 Hz, 1H, Ar). Mp 130-131 °C

4-Nitro-N'-phenylbenzohydrazide (3k)

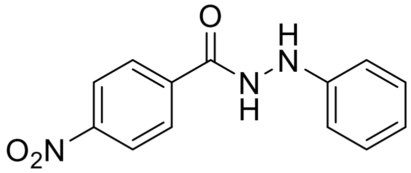

Compound **3k** was prepared according to the general procedure from phenylhydrazine (1.47 mL, 14.9 mmol) which was solubilized in pyridine (2.41 mL, 29.9 mmol) and 4-nitrobenzoyl chloride (2.53 g, 13.6 mmol) which was dissolved in 5 mL in THF. Yield 2.97 g (85%). Orange solid.

^{1}H NMR (400 Hz, DMSO-*d_6*): δ 10.70 (s, 1H, NH), 8.35 (d, J = 8.6 Hz, 2H, Ar), 8.14 (d, J = 8.6 Hz, 2H, Ar), 7.17 (t, J = 7.6 Hz, 2H, Ar), 6.81 (d, J = 7.6 Hz, 2H, Ar), 6.74 (t, J = 7.3 Hz, 1H, Ar). Mp 200-201 °C (lit. 198-200 °C [4])

N'-(4-Nitrophenyl)benzohydrazide (3l)

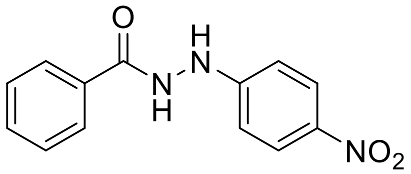

Compound **3l** was prepared according to the general procedure from (4-nitrophenyl)hydrazine (1.00 g,6.5 mmol) which was solubilized in pyridine (1.05 mL, 13.1 mmol) and benzoyl chloride (0.69 mL, 5.9 mmol) which was dissolved in 5 mL in THF. Yield 1.50 g (98%). White solid.
^{1}H NMR (400 Hz, DMSO-*d_6*): δ 10.65 (s, 1H, NH), 9.25 (s, 1H, NH), 8.12 – 8.06 (m, 2H, Ar), 7.97 – 7.91 (m, 2H, Ar), 7.65 – 7.58 (m, 1H, Ar), 7.54 (td, J = 7.6, 7.1, 1.7, 2H, Ar), 6.87 – 6.81 (m, 2H, Ar). Mp 192-194 °C (lit. 192-193 °C [7])

2-Chloro-5-nitro-N'-phenylbenzohydrazide (3m)

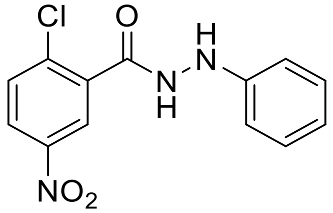

Compound **3m** was prepared according to the general procedure from 4-nitro-phenylhydrazine (0.44 mL,4.5 mmol) which was solubilized in Et_3N (0.62 mL, 4.5 mmol) and 2-chloro-5-nitrobenzoyl chloride (0.98 g, 4.5 mmol) which was dissolved in 5 mL in THF. Yield 1.22 g (93%). White solid.
^{1}H NMR (400 Hz, DMSO-*d_6*): δ 10.46 (d, J = 2.7 Hz, 1H, NH), 8.33 – 8.31 (m, 1H, Ar), 8.08 (d, J = 2.7 Hz, 1H, Ar), 7.88 (d, J = 8.8 Hz, 1H, Ar), 7.19 (t, J = 7.3 Hz, 2H, Ar), 6.86 (d, J = 7.6 Hz, 2H, Ar), 6.76 (t, J = 7.5 Hz, 1H, Ar). Mp 160-162 °C (lit. 159.9-161.6 °C)

4-Cyano-N'-phenylbenzohydrazide (3n)

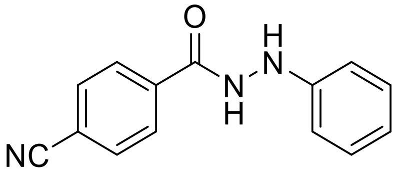

Compound **3n** was prepared according to the general procedure from phenylhydrazine (1.00 mL, 10.2 mmol) which was solubilized in pyridine (1.42 mL, 10.2 mmol) and 4-cyanobenzoyl chloride (1.69 g, 10.2 mmol) which was dissolved in 5 mL in THF. Yield 1.84 g (76%). Yellow solid.
^{1}H NMR (400 Hz, DMSO-*d_6*): δ 11.92 (s, 1H, NH), 10.61 (s, 1H, NH), 8.01 – 7.93 (m, 4H, Ar), 7.20 – 7.10 (m, 2H, Ar), 6.79 (d, J = 8.0 Hz, 2H, Ar), 6.72 (t, J = 7.1 Hz, 1H, Ar). Mp 165-167 °C

N'-(2,4-Dinitrophenyl)-4-methoxyphenylbenzohydrazide (3o)

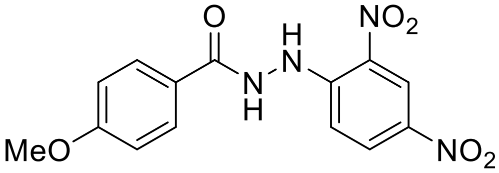

Compound **3o** was prepared according to the general procedure from (2,4-dinitrophenyl)hydrazine (0.90 mL, 4.5 mmol) which was solubilized in pyridine (0.73 mL, 9.1 mmol) and 4-methoxybenzoyl chloride (0.71 g, 4.1 mmol) which was dissolved in 5 mL in THF. Yield 1.88 g (86%). Yellow solid.

^{1}H NMR (400 Hz, DMSO-*d₆*): δ 10.92 (s, 1H, NH), 10.25 (s, 1H, NH), 8.88 (d, *J* = 2.7 Hz, 1H, Ar), 8.32 (dd, *J* = 9.6, 2.7 Hz, 1H, Ar), 7.95 (d, *J* = 8.8 Hz, 2H, Ar), 7.29 (d, *J* = 9.5 Hz, 1H, Ar), 7.08 (d, *J* = 8.6 Hz, 2H, Ar), 3.84 (s, 3H, OCH_3). Mp 210.5-213 °C (lit. 213-215 °C)

N'-Phenylacetohydrazide (3p)

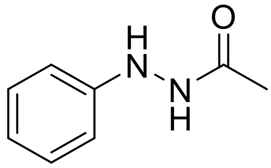

Phenylhydrazine (1.82 mL, 18.5 mmol) was solubilized in acetic acid (26.46 mL, 462.3 mmol) in a 50 mL flask. After addition, the temperature was allowed to warm to room temperature and the medium was stirred during 3.5 hours. **3p** gradually appeared in the medium. After completion of the reaction solvent was evaporated under reduced pressure. Next, DCM (50 mL) and NaOH (15% w/w, 15 mL) were added to the orange solid. The aqueous phase was extracted with DCM (2 × 30 mL). The combined organic layers were dried over Na_2SO_4 and the solvents evaporated in vacuo. Yield 1.79 g (65%). Orange solid.

^{1}H NMR (400 Hz, DMSO-*d₆*): δ 9.60 (s, 1H, NH), 7.15 – 7.09 (m, 2H, Ar), 6.71 – 6.66 (m, 3H, Ar), 1.89 (s, 3H, CH_3). Mp 128-130 °C (lit. 128.5-129.5 °C [8])

Synthesis of hydrazonoyl chlorides 4 [9]

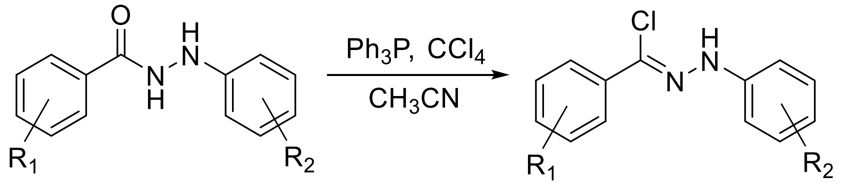

General procedure. To a suspension of benzoyl phenylhydrazine (**3a-o**) (1 equiv.) in anhydrous acetonitrile (3 mL) under a flow of nitrogen were added triphenylphosphine (1.25equiv.) and anhydrous carbon tetrachloride (1.25 mmol) and left to react overnight at room temperature. After the completion of the reaction solvent was evaporated under reduced pressure and the crude product was purified by column chromatography using appropriate mixtures of EtOAc and petroleum ether as eluents (for **4e**, **4h**, **4i**, **4m**, **4n**, **4o**) or the precipitate formed was collected by filtration (for all other compounds), washed by CH_3CN (3x10 mL) and dried, for compounds **4f**, **4g**, **4j**, **4k**, **4l** solvent of the remaining solution was removed in vacuo and the residue was recrystallized in CH_3CN or EtOH, the precipitate formed was collected by filtration, washed with CH_3CN (3x10 mL) and dried in air.

(Z)-4-Chloro-N-phenylbenzohydrazonoyl chloride (4a)

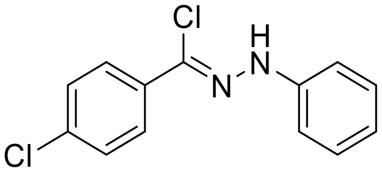

Compound **4a** was prepared according to the general procedure from suspension of **3a** (1.50 g, 6.1 mmol) in anhydrous acetonitrile under a flow of nitrogen, triphenylphosphine (1.99 g, 7.6 mmol) and anhydrous carbon tetrachloride (0,76 mL, 7.6 mmol). **4a** was precipitated by EtOH (10 mL), filtered, washed with CH_3CN and dried. Yield 1.34g (83%). White solid.

^{1}H NMR (400 Hz, DMSO-*d$_6$*): δ 9.97 (s, 1H, NH), 7.88 (d, J = 8.6 Hz, 2H, Ar), 7.53 (d, J = 8.6 Hz, 2H, Ar), 7.39 – 7.32 (m, 2H, Ar), 7.31 – 7.22 (m, 2H, Ar), 6.88 (tt, J = 7.2, 1.3 Hz, 1H, Ar). Mp 150-151 °C (lit. 151-152 °C [10])

(Z)-4-Bromo-N-phenylbenzohydrazonoyl chloride (4b)

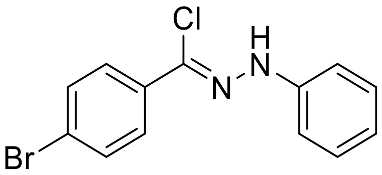

Compound **4b** was prepared according to the general procedure from suspension of **3b** (2.30g, 7.9 mmol) in anhydrous acetonitrile under a flow of nitrogen, triphenylphosphine (2.59 g, 9.8 mmol) and anhydrous carbon tetrachloride (0,95 mL, 9.8 mmol). After completion, the precipitate**4b**was collected by filtration, washed with CH_3CN and dried. Yield 1.73 g (73%). White solid.

^{1}H NMR (400 Hz, DMSO-*d$_6$*): δ 9.97 (s, 1H, NH), 7.85 – 7.77 (m, 2H, Ar), 7.69 – 7.59 (m, 2H, Ar), 7.38 – 7.31 (m, 2H, Ar), 7.31 – 7.22 (m, 2H, Ar), 6.87 (tt, J=7.2,1.2 Hz, 1H, Ar). Mp 143-145 °C (lit. 144-145 °C [11])

(Z)-4-Fluoro-N-phenylbenzohydrazonoyl chloride (4c)

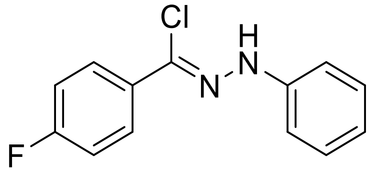

Compound **4c** was prepared according to the general procedure from suspension of **3c**(0.52 g, 2.2 mmol) in anhydrous acetonitrile under a flow of nitrogen, triphenylphosphine (0.74 g, 2.8 mmol) and anhydrous carbon tetrachloride (0.52 mL, 3.4 mmol). After completion, the precipitate **4c** was collected by filtration, washed with CH_3CN and dried. Yield 0.43g (80%). White solid.

^{1}H NMR (400 Hz, DMSO-*d$_6$*): δ 9.88 (s, 1H, NH), 7.96 – 7.88 (m, 2H, Ar), 7.38 – 7.22 (m, 6H, Ar), 6.87 (td, J = 7.5, 1.2 Hz, 1H, Ar).

(Z)-3-Fluoro-N-phenylbenzohydrazonoyl chloride (4d)

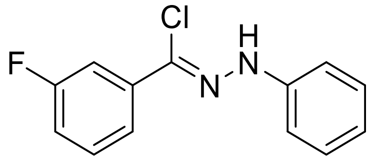

Compound **4d** was prepared according to the general procedure from suspension of **3d** (1.10 g, 4.7 mmol) in anhydrous acetonitrile under a flow of nitrogen, triphenylphosphine (1.56 g, 5.9 mmol) and anhydrous carbon tetrachloride (0.69 mL, 7.2 mmol). After completion, the precipitate **4d** was collected by filtration, washed with CH_3CN and dried. Yield 0.87 g (73%). White solid.

^{1}H NMR (400 Hz, DMSO-*d*$_6$): δ 10.01 (s, 1H, NH), 7.75 – 7.69 (m, 1H, Ar), 7.63 (dt, J = 10.6, 2.2 Hz, 1H, Ar), 7.50 (td, J = 8.1, 6.1 Hz, 1H, Ar), 7.40 – 7.33 (m, 2H, Ar), 7.31 – 7.21 (m, 3H, Ar), 6.89 (t, J = 7.3 Hz, 1H, Ar).

(Z)-2,4-Dichloro-N-phenylbenzohydrazonoyl chloride (4e)

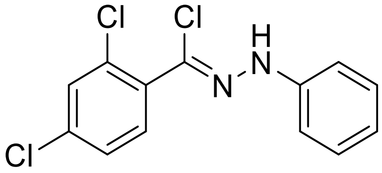

Compound **4e** was prepared according to the general procedure from suspension of **3e** (1.00g, 3.5 mmol) in anhydrous acetonitrile under a flow of nitrogen, triphenylphosphine (1.16 g, 4.4 mmol) and anhydrous carbon tetrachloride (0.43 mL,4.4 mmol). **4e** was purified by column chromatography with EtOAc/petroleum ether (1:10). Yield 0.71 g (67%). Pink-white solid.

^{1}H NMR (400 Hz, DMSO-*d*$_6$): δ 10.01 (s, 1H, NH), 7.78 (d, J = 2.1 Hz, 1H, Ar), 7.72 (d, J = 8.4 Hz, 1H, Ar), 7.55 (dd, J = 8.4, 2.2 Hz, 1H, Ar), 7.29 – 7.22 (m, 4H, Ar), 6.90 – 6.83 (m, 1H, Ar).

(Z)-4-Methyl-N-phenylbenzohydrazonoyl chloride (4f)

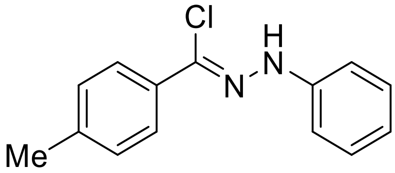

Compound **4f** was prepared according to the general procedure from suspension of **3f** (1.10g, 4.8 mmol) in anhydrous acetonitrile under a flow of nitrogen, triphenylphosphine (1.59 g, 6.1 mmol) and anhydrous carbon tetrachloride (0.59 mL, 6.1 mmol). After completion, the precipitate **4f** was collected by filtration, washed with CH_3CN and dried. Solvent of the remaining solution was removed in vacuo and the residue was recrystallized in CH_3CN, the precipitate formed was collected by filtration, washed with CH_3CN and dried. Yield 0.91 g (77%). White solid.

^{1}H NMR (400 Hz, DMSO-*d*$_6$): δ 9.79 (s, 1 H, NH), 7.77 (d, J=8.22 Hz, 2 H, Ar), 7.31 - 7.37 (m, 2 H, Ar), 7.21 - 7.30 (m, 4 H, Ar), 6.86 (t, J=7.18 Hz, 1 H, Ar), 2.34 (s, 3 H, CH_3). Mp 132-133 °C (lit. 134-136 °C [12])

(Z)-4-Methoxy-N-phenylbenzohydrazonoyl chloride (4g)

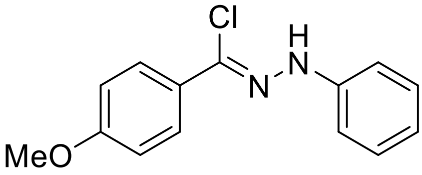

Compound **4g** was prepared according to the general procedure from suspension of **3g** (2.40g, 9.9 mmol) in anhydrous acetonitrile under a flow of nitrogen, triphenylphosphine (3.25 g, 12.4 mmol) and anhydrous carbon tetrachloride (1.44 mL, 14.8 mmol). After completion, the precipitate **4g** was collected by filtration, washed with CH_3CN and dried. Solvent of the remaining solution was removed in vacuo and the residue was recrystallized in CH_3CN, the precipitate formed was collected by filtration, washed with CH_3CN and dried. Yield 1.68 g (65%). White solid.

^{1}H NMR (400 Hz, DMSO-*d$_6$*): δ 9.72 (s, 1H, NH), 7.87 – 7.76 (m, 2H, Ar), 7.36 – 7.20 (m, 4H, Ar), 7.08 – 6.97 (m, 2H, Ar), 6.85 (t, J = 7.0 Hz, 1H, Ar), 3.81 (s, 3H, OCH_3). Mp 119-120 °C (lit. 117-121 °C [6])

(Z)-N-(4-Methoxyphenyl)benzohydrazonoyl chloride (4h)

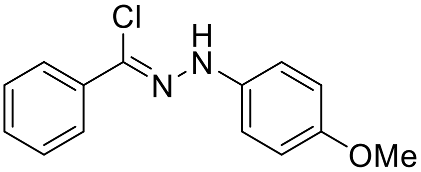

Compound **4h** was prepared according to the general procedure from suspension of **3h** (0.25g, 1.0 mmol) in anhydrous acetonitrile under a flow of nitrogen, triphenylphosphine (0.34 g, 1.3 mmol) and anhydrous carbon tetrachloride (0.15 mL, 1.5 mmol). **4h** was purified by column chromatography with EtOAc/petroleum ether (1:6). Yield 0.15 g (56%). White solid.

^{1}H NMR (400 Hz, DMSO-*d$_6$*): δ 9.70 (s, 1H, NH), 7.90 – 7.82 (m, 2H, Ar), 7.50 – 7.35 (m, 3H, Ar), 7.32 – 7.22 (m, 2H, Ar), 6.92 – 6.84 (m, 2H, Ar), 3.71 (s, 3H, OCH_3). Mp 95-97 °C (lit. 96-98 °C [13])

(Z)-3,4,5-Trimethoxy-N-phenylbenzohydrazonoyl chloride (4i)

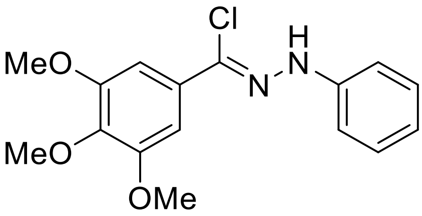

Compound **4i** was prepared according to the general procedure from suspension of **3i** (1.45g, 4.8 mmol) in anhydrous acetonitrile under a flow of nitrogen, triphenylphosphine (1.57 g, 5.9 mmol) and anhydrous carbon tetrachloride (0,58 mL, 5.9 mmol).**4i** was purified by column chromatography with EtOAc/petroleum ether (1:4). Yield 0.66g (43%). Yellow solid.

^{1}H NMR (400 Hz, DMSO-*d$_6$*): δ 9.83 (s, 1H, NH), 7.38 – 7.30 (m, 2H, Ar), 7.30 – 7.23 (m, 2H, Ar), 7.14 (s, 2H, Ar), 6.86 (tt, J = 7.2, 1.2 Hz, 1H, Ar), 3.86 (s, 6H, OCH_3), 3.71 (s, 3H, OCH_3).

(Z)-N-Phenyl-4-(trifluoromethyl)benzohydrazonoyl chloride (4j)

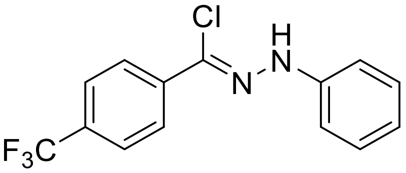

Compound **4j** was prepared according to the general procedure from suspension of **3j** (1.30g, 4.6 mmol) in anhydrous acetonitrile under a flow of nitrogen, triphenylphosphine (1.52 g, 5.8 mmol) and anhydrous carbon tetrachloride (0.56 mL, 5.8 mmol). After completion, the precipitate **4j** was collected by filtration, washed with CH_3CN and dried. Solvent of the remaining solution was removed in vacuo and the residue was recrystallized in CH_3CN, the precipitate formed was collected by filtration, washed with CH_3CN and dried. Yield 0.95 g (69%). White solid.

^{1}H NMR (400 Hz, DMSO-*d₆*): δ 10.16 (s, 1H, NH), 8.08 (d, J = 8.2 Hz, 2H, Ar), 7.82 (d, J = 8.4 Hz, 2H, Ar), 7.43 – 7.35 (m, 2H, Ar), 7.34 – 7.26 (m, 2H, Ar), 6.91 (t, J = 7.2 Hz, 1H, Ar).

(Z)-4-Nitro-N-phenylbenzohydrazonoyl chloride (4k)

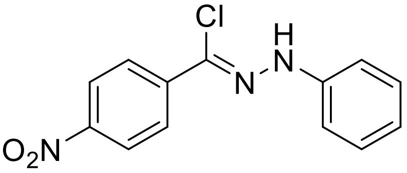

Compound **4k** was prepared according to the general procedure from suspension of **3k** (1.51g, 5.8 mmol) in anhydrous acetonitrile under a flow of nitrogen, triphenylphosphine (1.93 g, 7.3 mmol) and anhydrous carbon tetrachloride (0.71 mL, 7.3 mmol). After completion, the precipitate **4k** was collected by filtration, washed with CH_3CN and dried. Solvent of the remaining solution was removed in vacuo and the residue was recrystallized in EtOH, the precipitate formed was collected by filtration, washed with CH_3CN and dried. Yield 1.28 g (78%). Red solid.

^{1}H NMR (400 Hz, DMSO-*d₆*): δ 10.33 (s, 1H, NH), 8.20 (dd, J = 73.1, 9.0 Hz, 4H, Ar), 7.41 (d, J = 8.7 Hz, 2H, Ar), 7.31 (t, J = 8.0 Hz, 2H, Ar), 6.94 (t, J = 7.3 Hz, 1H, Ar). Mp 184-186 °C (lit. 184-188 °C [14])

(Z)-N-(4-Nitrophenyl)benzohydrazonoyl chloride (4l)

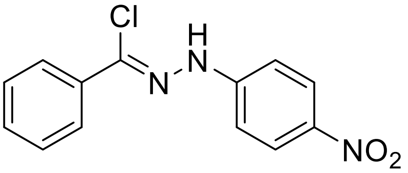

Compound **4l** was prepared according to the general procedure from suspension of **3l** (1.45g, 5.6 mmol) in anhydrous acetonitrile under a flow of nitrogen, triphenylphosphine (1.85 g, 7.0 mmol) and anhydrous carbon tetrachloride (0.68 mL, 7.0 mmol).After completion, the precipitate **4l** was collected by filtration, washed with $CH_3CN$and dried. Solvent of the remaining solution was removed in vacuo and the residue was recrystallized in EtOH, the precipitate formed was collected by filtration, washed with CH_3CN and dried. Yield 0.94 g (60%). Yellow solid.

^{1}H NMR (400 Hz, DMSO-*d₆*): δ 10.73 (s, 1 H, NH), 8.18 (d, J=9.21 Hz, 2 H, Ar), 7.95 (dd, J=7.56, 1.97 Hz, 2 H, Ar), 7.45 - 7.56 (m, 5 H, Ar). Mp 191-193 °C (lit. 190-195 °C [14])

(Z)-2-Chloro-5-nitro-N-phenylbenzohydrazonoyl chloride (4m)

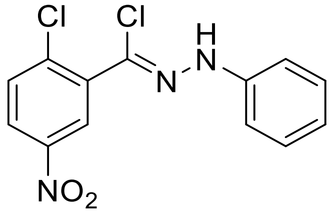

Compound **4m** was prepared according to the general procedure from suspension of **3m** (0.75g, 2.6 mmol) in anhydrous acetonitrile under a flow of nitrogen, triphenylphosphine (0.84 g, 3.2 mmol) and anhydrous carbon tetrachloride (0.31 mL, 3.2 mmol).**4m** was purified by column chromatography with EtOAc/petroleum ether (1:4). Yield 0.32 g (40%). Yellow solid.
^{1}H NMR (400 Hz, DMSO-*d$_6$*): δ 10.19 (s, 1H, NH), 8.48 (d, J = 2.7 Hz, 1H, Ar), 8.29 – 8.25 (m, 1H, Ar), 7.92 – 7.88 (m, 1H, Ar), 7.34 – 7.22 (m, 4H, Ar), 6.90 (tt, J = 6.8, 1.5 Hz, 1H, Ar).

(Z)-4-Cyano-N-phenylbenzohydrazonoyl chloride (4n)

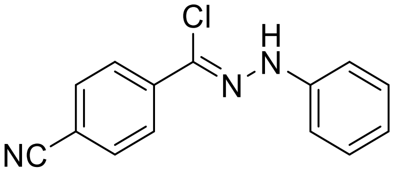

Compound **4n** was prepared according to the general procedure from suspension of **3n** (1.00g, 4.2 mmol) in anhydrous acetonitrile under a flow of nitrogen, triphenylphosphine (1.38 g, 5.3 mmol) and anhydrous carbon tetrachloride (0.51 mL, 5.3 mmol).**4m** was purified by column chromatography with EtOAc/petroleum ether (1:10). Yield 0.21 g (20%). Yellow solid.
^{1}H NMR (400 Hz, DMSO-*d$_6$*): δ 10.24 (s, 1H, NH), 8.04 (d, J = 8.2 Hz, 2H, Ar), 7.91 (d, J = 8.8 Hz, 2H, Ar), 7.40 (d, J = 7.6 Hz, 2H, Ar), 7.29 (t, J = 7.6 Hz, 2H, Ar), 6.92 (t, J = 7.0 Hz, 1H, Ar).

(Z)-N-(2,4-Dinitrophenyl)-4-methoxybenzohydrazonoyl chloride (4o)

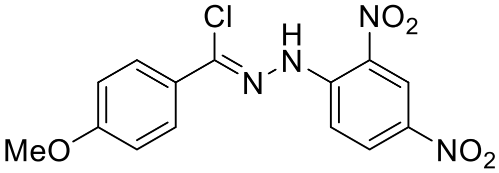

Compound **4o** was prepared according to the general procedure from suspension of **3o** (0.60 g, 1.8 mmol) in anhydrous acetonitrile under a flow of nitrogen, triphenylphosphine (0.61 g, 2.3 mmol) and anhydrous carbon tetrachloride (0.22 mL, 2.3 mmol). **4o** was purified by column chromatography with EtOAc/petroleum ether (1:10). Yield 0.21 g (32%). Red solid.
^{1}H NMR (400 Hz, DMSO-*d$_6$*): δ 11.41 (s, 1H, NH), 8.90 (d, *J* = 2.6 Hz, 1H), 8.46 (dd, *J* = 9.5, 2.7 Hz, 1H, Ar), 8.09 (d, *J* = 9.4 Hz, 1H, Ar), 8.03 – 7.95 (m, 2H, Ar), 7.11 (d, *J* = 9.0 Hz, 2H, Ar), 3.85 (s, 3H, OCH_3). Mp 203-205 °C (lit. 205-208 °C [15])

(Z)-N-Phenylacetohydrazonoyl chloride (4p)

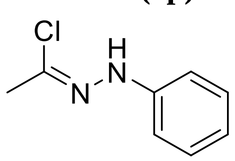

Compound **4p** was prepared according to the general procedure from suspension of **3p** (1.79 g, 11.9 mmol) in anhydrous acetonitrile under a flow of nitrogen, triphenylphosphine (3.91 g, 14.9 mmol) and anhydrous carbon tetrachloride (1.44 mL, 14.9 mmol). **4p** was purified by column chromatography with EtOAc/petroleum ether (1:6). Yield 1.49g (74%). Pink oil.
^{1}H NMR (400 Hz, DMSO-*d$_6$*): δ 9.20 (s, 1H, NH), 7.23 – 7.11 (m, 4H, Ar), 6.78 (tt, *J* = 7.1, 1.4 Hz, 1H, Ar), 2.36 (s, 3H, CH_3).

1,3-Diporal cycloaddition of nitrile imines 5 with 5-methylene-3-phenylhydantoin 6

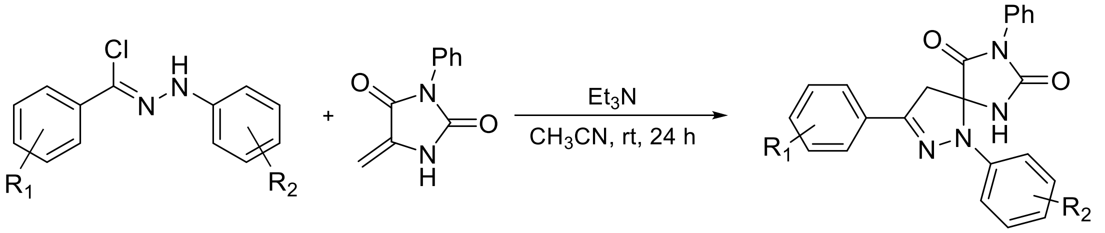

General procedure. Hydrazonoyl chloride (1.1 equiv.) and 5-methylene-3-phenylhydantoin (1 equiv.) are solubilized in acetonitrile (2 mL) under inert atmosphere and a solution of TEA (2.2 equiv.) in acetonitrile (2 mL) is added dropwise under stirring during 30 min. After the addition the reaction mixture is stirred for 24-48 hours, when solvent is removed in vacuo and the residue is purified by a column chromatography on silica gel with EtOAc/petroleum (EA/PE) ether or $MeOH/CHCl_3$ as eluent.

3-(4-Chlorophenyl)-1,8-diphenyl-1,2,6,8-tetraazaspiro[4.4]non-2-ene-7,9-dione (7a)

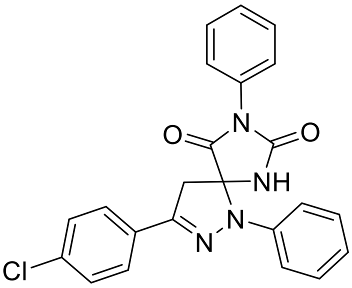

Compound **7a** was prepared from hydrazonoyl chloride **4a** (62 mg, 0.23 mmol), 5-methylene-3-phenylhydantoin (40 mg, 0.21 mmol) and TEA (0.065 mL, 0.47 mmol). Yield 88 mg (99%) White solid. Mp195 – 196°C. Chromatography: EA/PE, 1:10 – 1:6. R_f =0.32 (EA/PE, 1:4).
^{1}H NMR (400 Hz, DMSO-*d$_6$*): δ 9.59 (s, 1H, NH), 7.79 (d, J = 8.6 Hz, 2H, Ar), 7.60 – 7.51 (m, 4H, Ar), 7.49 – 7.43 (m, 1H, Ar), 7.41 – 7.33 (m, 4H, Ar), 7.13 (d, J = 7.2 Hz, 2H, Ar), 7.03 (t, J = 7.3, 1.2 Hz, 1H, Ar), 3.95 (d, J = 18.3 Hz, 1H, CH_2), 3.77 (d, J = 18.3 Hz, 1H, CH_2). **^{13}C NMR (100 MHz, DMSO-*d$_6$*):** δ 171.2, 153.4, 146.8, 142.4, 133.9, 131.4, 130.2, 129.4, 129.1, 129.0, 128.4, 127.6, 126.5, 122.5, 116.4, 81.5, 44.6. **HRMS (ESI):** calcd for$C_{23}H_{17}ClN_4O_2$ $(M+H)^+$ 417.1113, found 417.1109.

3-(4-Bromophenyl)-1,8-diphenyl-1,2,6,8-tetraazaspiro[4.4]non-2-ene-7,9-dione (7b)

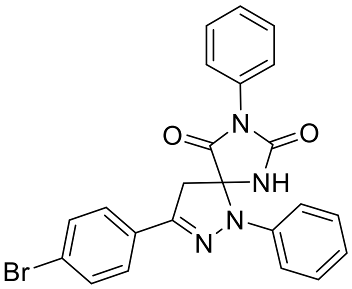

Compound **7b** was prepared from hydrazonoyl chloride **4b** (105 mg, 0.35 mmol), 5-methylene-3-phenylhydantoin (60 mg, 0.32 mmol) and TEA (0.097 mL, 0.70 mmol). Yield 137 mg (95%). White solid. Mp 205 – 206°C. Chromatography: EA/PE, 1:8 – 1:4. R_f= 0.28 (EA/PE, 1:4).

^{1}H NMR (400 Hz, DMSO-*d$_6$*): δ 9.57 (s, 1H, NH), 7.74 – 7.66 (m, 4H, Ar), 7.57 – 7.51 (m, 2H, Ar), 7.49 – 7.43 (m, 1H, Ar), 7.41 – 7.33 (m, 4H, Ar), 7.13 (d, J = 7.7 Hz, 2H, Ar), 7.03 (t, J = 7.4 Hz, 1H, Ar), 3.95 (d, J = 18.3 Hz, 1H, CH_2), 3.77 (d, J = 18.3 Hz, 1H, CH_2). **^{13}C NMR (100 MHz, DMSO-*d$_6$*):** δ 171.2, 153.4, 146.8, 142.4, 131.9, 131.4, 130.6, 129.4, 129.2, 128.5, 127.9, 126.5, 122.7, 122.5, 116.5, 81.5, 44.6. **HRMS (ESI):** calcd for $C_{23}H_{17}BrN_4O_2$ $(M+H)^+$ 461.0608, found 461.0601.

3-(4-Fluorophenyl)-1,8-diphenyl-1,2,6,8-tetraazaspiro[4.4]non-2-ene-7,9-dione (7c)

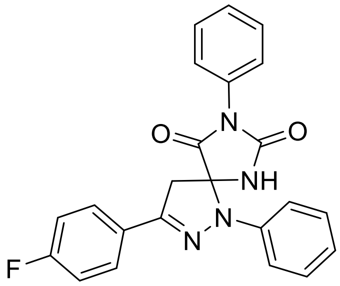

Compound **7c** was prepared from hydrazonoyl chloride **4c** (58 mg, 0.23 mmol), 5-methylene-3-phenylhydantoin (40 mg, 0.21 mmol) and TEA (0.065 mL, 0.47 mmol). Yield 68 mg (81%). White solid. Mp 192 – 194°C. Chromatography: EA/PE, 1:8 – 1:4. Rf = 0.25 (EA/PE, 1:4).

^{1}H NMR (400 Hz, DMSO-*d$_6$*): δ 9.58 (s, 1H, NH), 7.86 – 7.79 (m, 2H, Ar), 7.58 – 7.50 (m, 2H, Ar), 7.49 – 7.43 (m, 1H, Ar), 7.40 – 7.30 (m, 6H, Ar), 7.13 (d, J = 7.8 Hz, 2H, Ar), 7.03 (t, J = 7.3 Hz, 1H, Ar), 3.96 (d, J = 18.2 Hz, 1H, CH_2), 3.78 (d, J = 18.3 Hz, 1H, CH_2). **^{13}C NMR (100 MHz, DMSO-*d$_6$*):** δ 171.2, 164.0, 161.5, 153.4, 146.9, 142.5, 131.4, 129.3, 128.7 (d, J = 70.0 Hz), 128.1 (d, J = 8.5 Hz), 127.9 (d, J = 3.1 Hz), 126.5, 122.3, 116.4, 115.9 (d, J = 21.9 Hz), 81.5, 44.8. **HRMS (ESI):** calcd for $C_{23}H_{17}FN_4O_2$ $(M+H)^+$ 401.1408, found 401.1406.

3-(3-Fluorophenyl)-1,8-diphenyl-1,2,6,8-tetraazaspiro[4.4]non-2-ene-7,9-dione (7d)

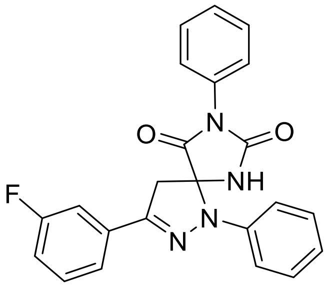

Compound **7d** was prepared from hydrazonoyl chloride **4d** (58 mg, 0.23 mmol), 5-methylene-3-phenylhydantoin (40 mg, 0.21 mmol) and TEA (0.065 mL, 0.47 mmol). Yield 74 mg (88%). White solid. Mp 198 – 199 °C. Recrystallized from DCM.

^{1}H NMR (400 Hz, DMSO-*d₆*): δ 9.60 (s, 1H, NH), 8.32 (s, 1H, Ar), 7.64 – 7.49 (m, 4H, Ar), 7.49 – 7.42 (m, 1H, Ar), 7.41 – 7.33 (m, 4H, Ar), 7.33 – 7.25 (m, 1H, Ar), 7.18 – 7.11 (m, 2H, Ar), 7.04 (t, J = 7.4 Hz, 1H, Ar), 3.96 (d, J = 18.3 Hz, 1H, CH_2), 3.79 (d, J = 18.3 Hz, 1H, CH_2). **^{13}C NMR (100 MHz, DMSO-d_6):** δ 171.1, 162.3 (d, Jcf = 243.8 Hz), 153.4, 146.7, 142.3, 133.7 (d, J = 8.1 Hz), 131.4, 130.9 (d, J = 8.3 Hz), 129.3, 129.2 (d, J = 24.9 Hz), 126.5, 122.5, 122.1, 116.5, 116.2 (d, J = 21.3 Hz), 112.3 (d, J = 23.0 Hz), 81.5, 79.2, 44.6. **HRMS (ESI):** calcd for $C_{23}H_{17}FN_4O_2$ $(M+H)^+$ 401.1408, found 401.1406.

3-(2,4-Dichlorophenyl)-1,8-diphenyl-1,2,6,8-tetraazaspiro[4.4]non-2-ene-7,9-dione (7e)

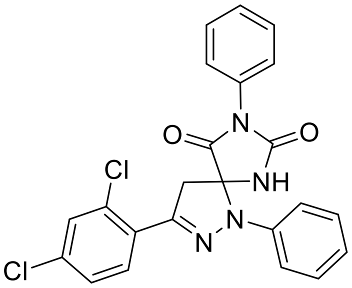

Compound **7e** was prepared from hydrazonoyl chloride **4e** (105 mg, 0.35 mmol), 5-methylene-3-phenylhydantoin (60 mg, 0.32 mmol) and TEA (0.97 mL, 0.070 mmol). Yield 135 mg (94%). White solid. Mp 173 – 175 °C. Chromatography: EA/PE, 1:10 – 1:6. R_f = 0.13 (EA/PE, 1:8).

^{1}H NMR (400 Hz, DMSO-d_6): δ 9.61 (s, 1H, NH), 7.87 (d, J = 8.5 Hz, 1H, Ar), 7.77 (d, J = 2.2 Hz, 1H, Ar), 7.60 – 7.50 (m, 3H, Ar), 7.49 – 7.43 (m, 1H, Ar), 7.42 – 7.35 (m, 4H, Ar), 7.14 (d, J = 7.7 Hz, 2H, Ar), 7.06 (t, J = 7.3, 1.1 Hz, 1H, Ar), 4.09 (d, J = 18.3 Hz, 1H, CH_2), 3.88 (d, J = 18.3 Hz, 1H, CH_2). **^{13}C NMR (100 MHz, DMSO-d_6):** δ 171.56, 153.86, 145.61, 142.52, 134.64, 132.43, 131.93, 131.83, 130.84, 129.85, 129.54, 129.32, 128.89, 128.18, 127.02, 123.25, 117.13, 81.99, 47.29. **HRMS (ESI):** calcd for $C_{23}H_{16}Cl_2N_4O_2$ $(M+H)^+$ 451.0723, found 451.0721.

1,8-Diphenyl-3-(p-tolyl)-1,2,6,8-tetraazaspiro[4.4]non-2-ene-7,9-dione (7f)

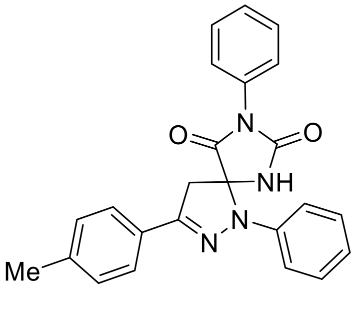

Compound **7f** was prepared from hydrazonoyl chloride **4f** (74 mg, 0.29 mmol), 5-methylene-3-phenylhydantoin (50 mg, 0.26 mmol) and TEA (0.080 mL, 0.58 mmol). Yield 90 mg (86%). White solid. Mp 186 – 187 °C. Chromatography: EA/PE, 1:8 – 1:1. R_f = 0.30 (EA/PE, 1:4).
^{1}H NMR (400 Hz, DMSO-*d$_6$*): δ 9.57 (s, 1H, NH), 7.67 (d, J = 8.0 Hz, 2H, Ar), 7.54 (t, J = 7.7 Hz, 2H, Ar), 7.49 – 7.42 (m, 1H, Ar), 7.40 – 7.27 (m, 6H, Ar), 7.12 (d, J = 8.0 Hz, 2H, Ar), 7.01 (t, J = 7.3 Hz, 1H, Ar), 3.92 (d, J = 18.2 Hz, 1H, CH_2), 3.75 (d, J = 18.2 Hz, 1H, CH_2), 2.37 (s, 3H, CH_3). **^{13}C NMR (100 MHz, DMSO-*d$_6$*):** δ 171.4, 153.5, 147.8, 142.7, 139.2, 131.4, 129.5, 129.3, 129.1, 128.6, 128.4, 126.5, 125.9, 122.1, 116.2, 81.2, 45.0, 21.1. **HRMS (ESI):** calcd for $C_{24}H_{20}N_4O_2$ $(M+H)^+$ 397.1659, found 397.1658.

3-(4-Methoxyphenyl)-1,8-diphenyl-1,2,6,8-tetraazaspiro[4.4]non-2-ene-7,9-dione (7g)

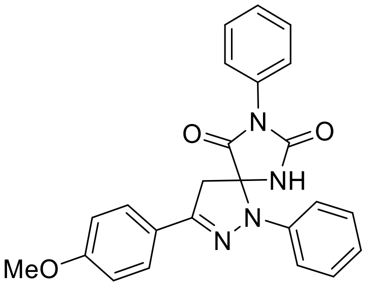

Compound **7g** was prepared from hydrazonoyl chloride **4g** (61 mg, 0.23 mmol), 5-methylene-3-phenylhydantoin (40 mg, 0.21 mmol) and TEA (0.065 mL, 0.47 mmol). Yield 70 mg (81%). White solid. Mp 184 – 186 °C. Chromatography: $CHCl_3$ – MeOH/$CHCl_3$, 1:100. R_f = 0.20 ($CHCl_3$).
^{1}H NMR (400 Hz, DMSO-*d$_6$*): δ 9.57 (s, 1H, NH), 7.73 (d, J = 8.7 Hz, 2H, Ar), 7.58 – 7.51 (m, 2H, Ar), 7.49 – 7.43 (m, 1H, Ar), 7.42 – 7.31 (m, 4H, Ar), 7.13 (d, J = 7.6 Hz, 2H, Ar), 7.06 (d, J = 8.8 Hz, 2H, Ar), 7.01 (t, J = 7.4 Hz, 1H, Ar), 3.93 (d, J = 18.1 Hz, 1H, CH_2), 3.83 (s, 3H, CH_3), 3.76 (d, J = 18.1 Hz, 1H, CH_2). **^{13}C NMR (100 MHz, DMSO-*d$_6$*):** δ 171.9, 160.8, 153.9, 148.1, 143.3, 131.9, 129.7, 129.5, 128.8, 128.0, 127.0, 124.3, 122.4, 116.6, 114.8, 81.6, 55.8, 45.5. **HRMS (ESI):** calcd for $C_{24}H_{20}N_4O_3$ $(M+H)^+$ 413.1608, found 413.1613.

1-(4-Methoxyphenyl)-3,8-diphenyl-1,2,6,8-tetraazaspiro[4.4]non-2-ene-7,9-dione (7h)

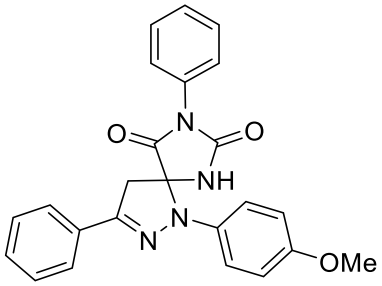

Compound **7h** was prepared from hydrazonoyl chloride **4h** (84 mg, 0.32 mmol), 5-methylene-3-phenylhydantoin (55 mg, 0.29 mmol) and TEA (0.089 mL, 0.64 mmol). Yield 88 mg (73%). White solid. Mp 191 – 193 °C. Chromatography: $CHCl_3$– MeOH/$CHCl_3$, 1:100. R_f = 0.23 ($CHCl_3$).

^{1}H NMR (400 Hz, DMSO-*d₆*): δ 9.52 (s, 1H, NH), 7.81 – 7.69 (m, 2H, Ar), 7.57 – 7.39 (m, 6H, Ar), 7.35 – 7.25 (m, 2H, Ar), 7.09 (d, J = 9.0 Hz, 2H, Ar), 6.97 (d, J = 9.1 Hz, 2H, Ar), 3.87 (d, J = 17.9 Hz, 1H, CH_2), 3.75 (s, 3H, CH_3), 3.72(d, J = 17.9 Hz, 2H, CH_2). **^{13}C NMR (100 MHz, DMSO-d_6):** δ 171.6, 156.0, 153.7, 147.8, 136.2, 131.8, 131.7, 129.5, 129.3, 129.1, 128.6, 126.8, 126.0, 120.5, 114.7, 82.8, 55.5, 44.1. **HRMS (ESI):** calcd for $C_{24}H_{20}N_4O_3$ $(M+H)^+$ 413.1608, found 413.1611.

1,8-Diphenyl-3-(3,4,5-trimethoxyphenyl)-1,2,6,8-tetraazaspiro[4.4]non-2-ene-7,9-dione (7i)

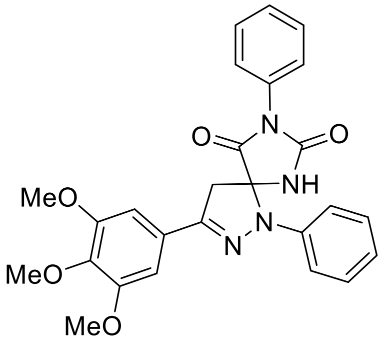

Compound **7i** was prepared from hydrazonoyl chloride **4i** (56 mg, 0.18 mmol), 5-methylene-3-phenylhydantoin (30 mg, 0.16 mmol) and TEA (0.049 mL, 0.35 mmol). Yield 64 mg (85%). White solid. Mp 206 – 207 °C. Chromatography: MeOH/$CHCl_3$, 1:100 – 1:50. R_f = 0.10 (EA/PE, 1:4).

^{1}H NMR (400 Hz, DMSO-d_6): δ 9.62 (s, 1H, NH), 7.55 (t, J = 7.6 Hz, 2H, Ar), 7.50 – 7.42 (m, 1H, Ar), 7.41 – 7.33 (m, 4H, Ar), 7.14 (d, J = 8.0 Hz, 2H, Ar), 7.08 – 6.97 (m, 3H, Ar), 3.99 (d, J = 18.3 Hz, 1H, CH_2), 3.86 (s, 6H, m-OCH_3), 3.82 (d, J = 18.7 Hz, 1H, CH_2), 3.71 (s, 3H, p-OCH_3). **^{13}C NMR (100 MHz, DMSO-d_6):** δ 171.3, 153.4, 153.1, 147.8, 142.6, 138.8, 131.4, 129.4, 129.2, 128.5, 126.8, 126.6, 122.2, 116.2, 103.4, 81.3, 60.2, 56.0, 45.2. **HRMS (ESI):** calcd for $C_{26}H_{24}N_4O_5$ $(M+H)^+$ 473.1820, found 473.1812.

1,8-Diphenyl-3-(4-(trifluoromethyl)phenyl)-1,2,6,8-tetraazaspiro[4.4]non-2-ene-7,9-dione (7j)

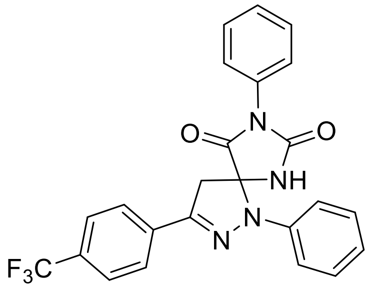

Compound **7j** was prepared from hydrazonoyl chloride **4j** (222 mg, 0.74 mmol), 5-methylene-3-phenylhydantoin (70 mg, 0.37 mmol) and TEA (0.207 mL, 1.49 mmol). Yield 100 mg (60%). White solid. Mp 189 – 191 °C. Chromatography: EA/PE, 1:12 – 1:6. Rf = 0.35 (EA/PE, 1:4).
^{1}H NMR (400 Hz, DMSO-*d₆*): δ 9.64 (s, 1H, NH), 7.97 (d, J = 8.1 Hz, 2H, Ar), 7.85 (d, J = 8.2 Hz, 2H, Ar), 7.59 – 7.51 (m, 2H, Ar), 7.49 – 7.43 (m, 1H, Ar), 7.43 – 7.35 (m, 4H, Ar), 7.17 (d, J = 7.9 Hz, 2H, Ar), 7.06 (t, J = 7.3 Hz, 1H, Ar), 4.02 (d, J = 18.3 Hz, 1H, CH_2), 3.83 (d, J = 18.3 Hz, 1H, CH_2). **^{13}C NMR (100 MHz, DMSO-d_6):** δ 171.1, 153.4, 146.4, 142.1, 135.2, 131.4, 129.4, 129.1, 129.2 (q, J = 31 Hz), 128.4, 128.2, 126.5, 126.5, 125.8 (q, J = 3.8 Hz), 124.6 (q, Jcf = 272 Hz), 122.8, 116.6, 81.6, 44.4. **HRMS (ESI):** calcd for $C_{24}H_{17}F_3N_4O_2$ $(M+H)^+$ 451.1376, found 451.1368.

3-(4-Nitrophenyl)-1,8-diphenyl-1,2,6,8-tetraazaspiro[4.4]non-2-ene-7,9-dione (7k)

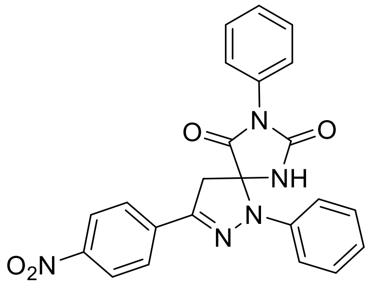

Compound **7k** was prepared from hydrazonoyl chloride **4k** (79 mg, 0.26 mmol), 5-methylene-3-phenylhydantoin (45 mg, 0.24 mmol) and TEA (0.073 mL, 0.53 mmol). Yield 90 mg (83%). Yellow solid. Mp 231 – 233 °C. Chromatography: EA/PE, 1:10 – 1:4. Rf = 0.15 (EA/PE, 1:4).
^{1}H NMR (400 Hz, DMSO-d_6): δ 9.64 (s, 1H, NH), 8.33 (d, J = 8.8 Hz, 2H, Ar), 8.00 (d, J = 8.6 Hz, 2H, Ar), 7.58 – 7.51 (m, 2H, Ar), 7.50 – 7.37 (m, 5H, Ar), 7.18 (d, J = 8.0 Hz, 2H, Ar), 7.08 (t, J = 7.3 Hz, 1H, Ar), 4.03 (d, J = 18.3 Hz, 1H, CH_2), 3.85 (d, J = 18.4 Hz, 1H, CH_2).**^{13}C NMR (100 MHz, DMSO-d_6):** δ 170.9, 153.4, 147.3, 145.9, 141.8, 137.5, 131.4, 129.5, 129.1, 128.5, 126.8, 126.5, 124.2, 123.1, 116.8, 81.8, 44.2. **HRMS (ESI):** calcd for $C_{23}H_{17}N_5O_4$ $(M+H)_+$ 428.1353, found 428.1350.

1-(4-nitrophenyl)-3,8-diphenyl-1,2,6,8-tetraazaspiro[4.4]non-2-ene-7,9-dione (7l) and 1-(4-Nitrophenyl)-N,3-diphenyl-1H-pyrazole-5-carboxamide (9a)

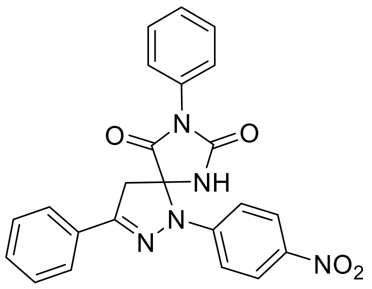

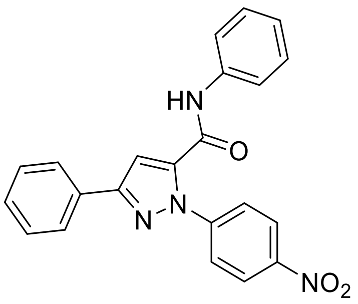

Compounds **7l** and **9a** were prepared from hydrazonoyl chloride **4l** (129 mg, 0.47 mmol), 5-methylene-3-phenylhydantoin (80 mg, 0.43 mmol) and TEA (0.130 mL, 0.94 mmol) and separated by a column chromatography on silica gel using EtOAc/petroleum (1:10 – 1:4).
Yield of compound **7l** 66 mg (36%). Yellow solid. Mp 198 – 200 °C. R_f = 0.35 (EA/PE, 1:4).
^{1}H NMR (400 Hz, DMSO-*d₆*): δ 9.69 (s, 1H, NH), 8.30 (d, J = 9.4 Hz, 2H, Ar), 7.88 – 7.80 (m, 2H, Ar), 7.61 – 7.45 (m, 8H, Ar), 7.24 (d, J = 9.4 Hz, 2H, Ar), 4.15 (d, J = 18.8 Hz, 1H, CH_2), 3.93 (d, J = 18.8 Hz, 1H, CH_2). **^{13}C NMR (100 MHz, DMSO-d_6):** δ 170.4, 153.3, 150.9, 147.1, 140.2, 131.2, 130.5, 130.3, 129.1, 129.0, 128.7, 126.9, 126.4, 126.1, 113.3, 79.9, 46.0. **HRMS (ESI):** calcd for $C_{23}H_{17}N_5O_4$ $(M+H)^+$ 428.1353, found 428.1352.

Yield of compound **9a** 28 mg (23%). Red solid. Dp 250 – 251 °C. Rf = 0.6 (EA/PE, 1:4).
^{1}H NMR (400 Hz, DMSO-d_6): δ 10.82 (s, 1H, NH), 8.36 (dt, *J* = 9.2, 2.9 Hz, 2H, Ar), 7.99 – 7.92 (m, 2H, Ar), 7.85 (dt, *J* = 9.0, 2.2 Hz, 2H, Ar), 7.72 – 7.67 (m, 2H, Ar), 7.67 (s, 1H, CH),7.54 – 7.46 (m, 2H, Ar), 7.43 (tt, *J* = 7.5, 1.4 Hz, 1H, Ar), 7.39 – 7.31 (m, 2H, Ar), 7.14 (tt, *J* = 7.7, 1.2 Hz, 1H, Ar). **^{13}C NMR (100 MHz, DMSO-d_6):** δ 158.1, 152.1, 146.6, 144.9, 139.6, 138.7, 131.9, 129.5, 129.3, 129.3, 126.0, 125.2, 125.0, 124.8, 120.6, 108.8. **HRMS (ESI):** calcd for $C_{22}H_{16}N_4O_3$ $(M+H)^+$ 385.1295, found 385.1299.

3-(2-Chloro-5-nitrophenyl)-1,8-diphenyl-1,2,6,8-tetraazaspiro[4.4]non-2-ene-7,9-dione (7m)

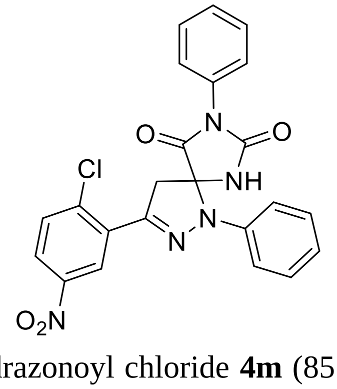

Compound **7m** was prepared from hydrazonoyl chloride **4m** (85 mg, 0.27 mmol), 5-methylene-3-phenylhydantoin (47 mg, 0.25 mmol) and TEA (0.076 mL, 0.55 mmol). Yield 40 mg (35%). Yellow solid. Mp 196 – 198 °C. Chromatography: EA/PE, 1:8 – 1:2. R_f = 0.31 (EA/PE, 1:4)
^{1}H NMR (400 Hz, DMSO-d_6): δ 9.63 (s, 1H, NH), 8.57 (d, J = 2.7 Hz, 1H, Ar), 8.26 (dd, J = 8.8, 2.8 Hz, 1H, Ar), 7.90 (d, J = 8.9 Hz, 1H, Ar), 7.55 (t, J = 7.7 Hz, 2H, Ar), 7.49 – 7.36 (m, 5H, Ar), 7.16 (d, J = 7.9 Hz, 2H, Ar), 7.09 (t, J = 7.4 Hz, 1H, Ar), 4.18 (d, J = 18.3 Hz, 1H, CH_2), 3.96 (d, J = 18.4 Hz, 1H, CH_2). **^{13}C NMR (100 MHz, DMSO-d_6):** δ 171.3, 153.8, 146.9,

145.1, 142.2, 138.0, 133.0, 131.8, 131.7, 129.9, 129.5, 128.9, 127.0, 125.0, 123.6, 117.4, 82.3, 47.0. **HRMS (ESI):** calcd for $C_{23}H_{16}ClN_5O_4$ $(M+H)^+$ 462.0964, found 462.0966.

4-(7,9-Dioxo-1,8-diphenyl-1,2,6,8-tetraazaspiro[4.4]non-2-en-3-yl)benzonitrile (7n)

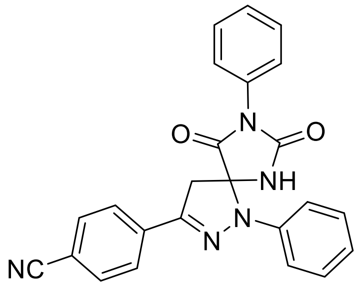

Compound **7n** was prepared from hydrazonoyl chloride **4n** (90 mg, 0.35 mmol), 5-methylene-3-phenylhydantoin (60 mg, 0.32 mmol) and TEA (0.097 mL, 0.70 mmol). Yield 42 mg (32%). White solid. Mp 243 – 244 °C. Chromatography: EA/PE, 1:10 – 1:4. R_f = 0.3 (EA/PE, 1:4).
^{1}H NMR (400 Hz, DMSO-*d₆*): δ 9.63 (s, 1H, NH), 7.98 – 7.90 (m, 4H, Ar), 7.58 – 7.52 (m, 2H, Ar), 7.49 – 7.43 (m, 1H, Ar), 7.43 – 7.35 (m, 4H, Ar), 7.17 (d, J = 7.6 Hz, 2H, Ar), 7.07 (t, J = 7.3 Hz, 1H, Ar), 4.00 (d, J = 18.1 Hz, 1H, CH_2), 3.82 (d, J = 18.4 Hz, 1H, CH_2).**^{13}C NMR (100 MHz, DMSO-d_6): δ** 171.0, 153.4, 146.2, 142.0, 135.6, 132.8, 131.4, 129.5, 129.2, 128.5, 126.5, 126.5, 122.9, 118.8, 116.7, 111.2, 81.7, 44.2. **HRMS (ESI):** calcd for $C_{24}H_{17}N_5O_2$ $(M+H)^+$ 408.1455, found 408.1459.

1-(2,4-dinitrophenyl)-3-(4-methoxyphenyl)-8-phenyl-1,2,6,8-tetraazaspiro[4.4]non-2-ene-7,9-dione (7o)

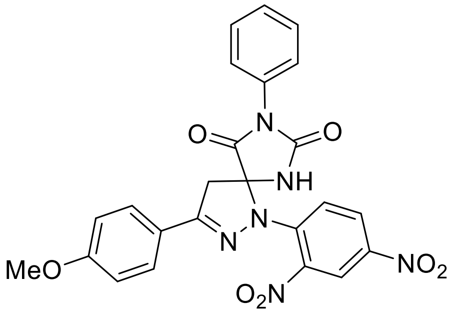

Compound **7o** was not obtained in the reaction of hydrazonoyl chloride **4o** (144 mg, 0.41 mmol), 5-methylene-3-phenylhydantoin (70 mg, 0.37 mmol) and TEA (0.114 mL, 0.82 mmol). As a result of the reaction complex mixture of different products was obtained.

3-Methyl-1,8-diphenyl-1,2,6,8-tetraazaspiro[4.4]non-2-ene-7,9-dione (7p)

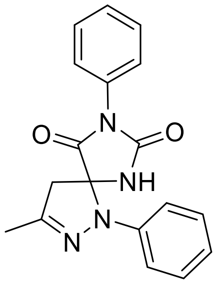

Compound **7p** was prepared from hydrazonoyl chloride **4p** (19 mg, 0.11 mmol), 5-methylene-3-phenylhydantoin (19 mg, 0.10 mmol) and TEA (0.031 mL, 0.22 mmol). Yield 20 mg (63%).

Orange solid. Mp 85 – 87 °C. Chromatography: MeOH/$CHCl_3$, 1:100 – 1:50. R_f = 0.37 (MeOH/$CHCl_3$, 1:4).
^{1}H NMR (400 Hz, $CDCl_3$): δ 7.48 – 7.41 (m, 2H, Ar), 7.40 – 7.32 (m, 1H, Ar), 7.30 – 7.21 (m, 4H, Ar), 7.12 – 7.06 (m, 2H, Ar), 7.06 – 7.030(m, 1H, Ar), 6.83 (bs, 1H), 3.54 (dd, J – 17.9, 1.4 Hz, 1H, CH_2), 3.01 (d, J – 17.9 Hz, 1H, CH_2), 2.02 (s, 3H, CH_3). **^{13}C NMR (100 MHz, $CDCl_3$):** δ 171.1, 154.2, 148.9, 142.9, 130.9, 129.3, 129.3, 128.6, 125.9, 123.6, 118.1, 81.9, 48.6, 15.5. **HRMS (ESI):** calcd for $C_{18}H_{16}N_4O_2$ $(M+H)^+$ 321.1346, found 321.1355.

Characterization data of obtained spiro products

^{1}H NMR spectrum of **7a** (400 Hz, DMSO-d_6)

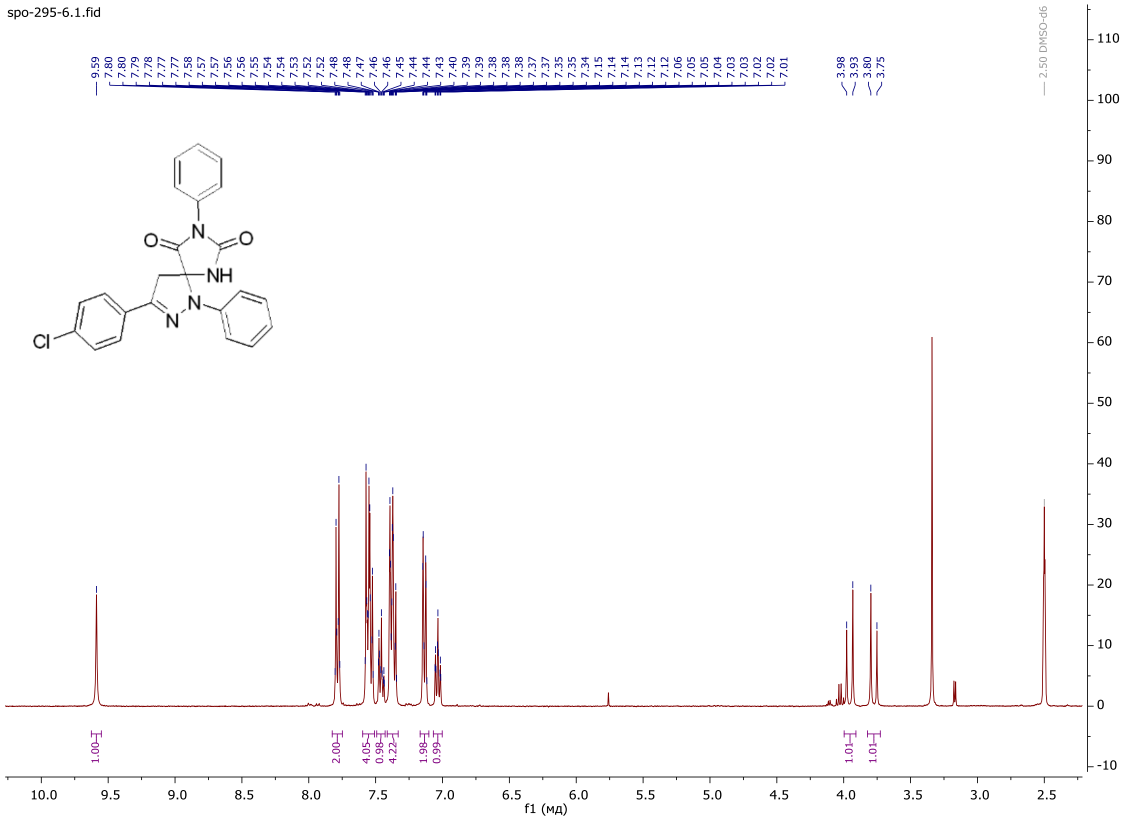

^{13}C NMR spectrum of **7a** (100 MHz, DMSO-d_6)

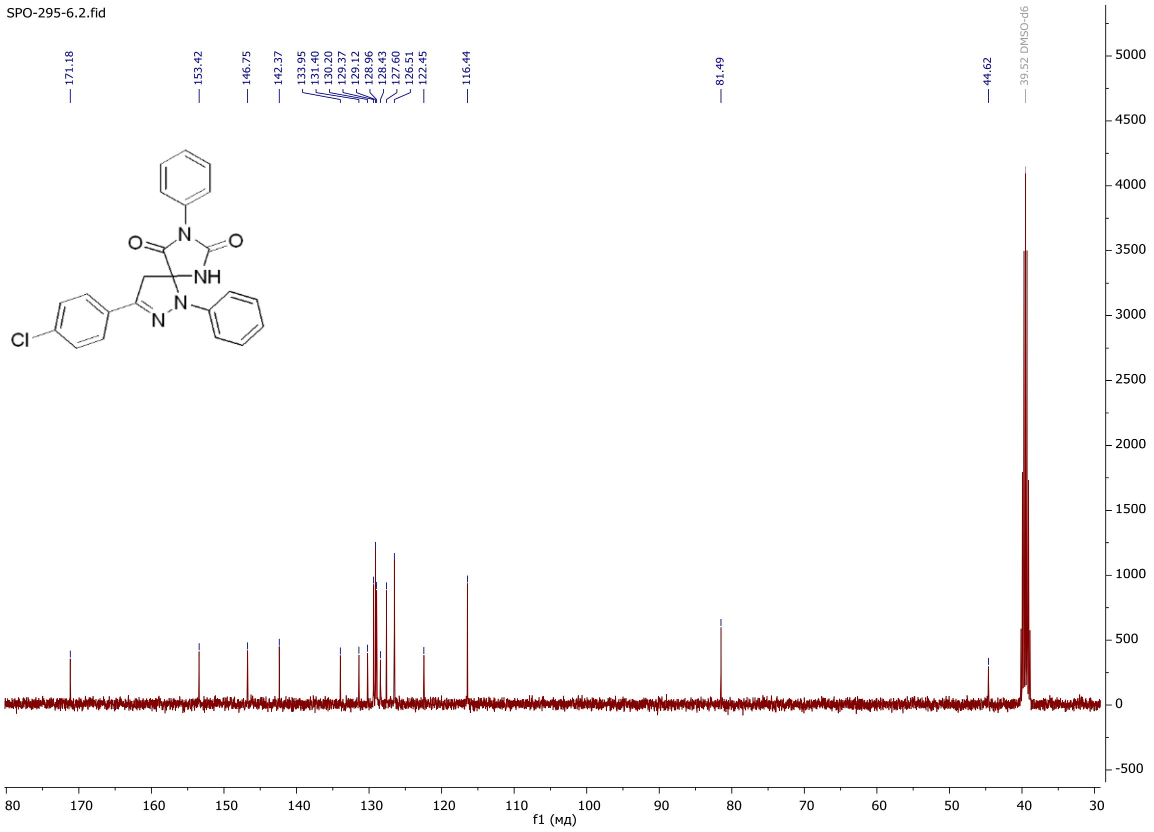

^{1}H NMR spectrum of **7b** (400 Hz, DMSO-d_6)

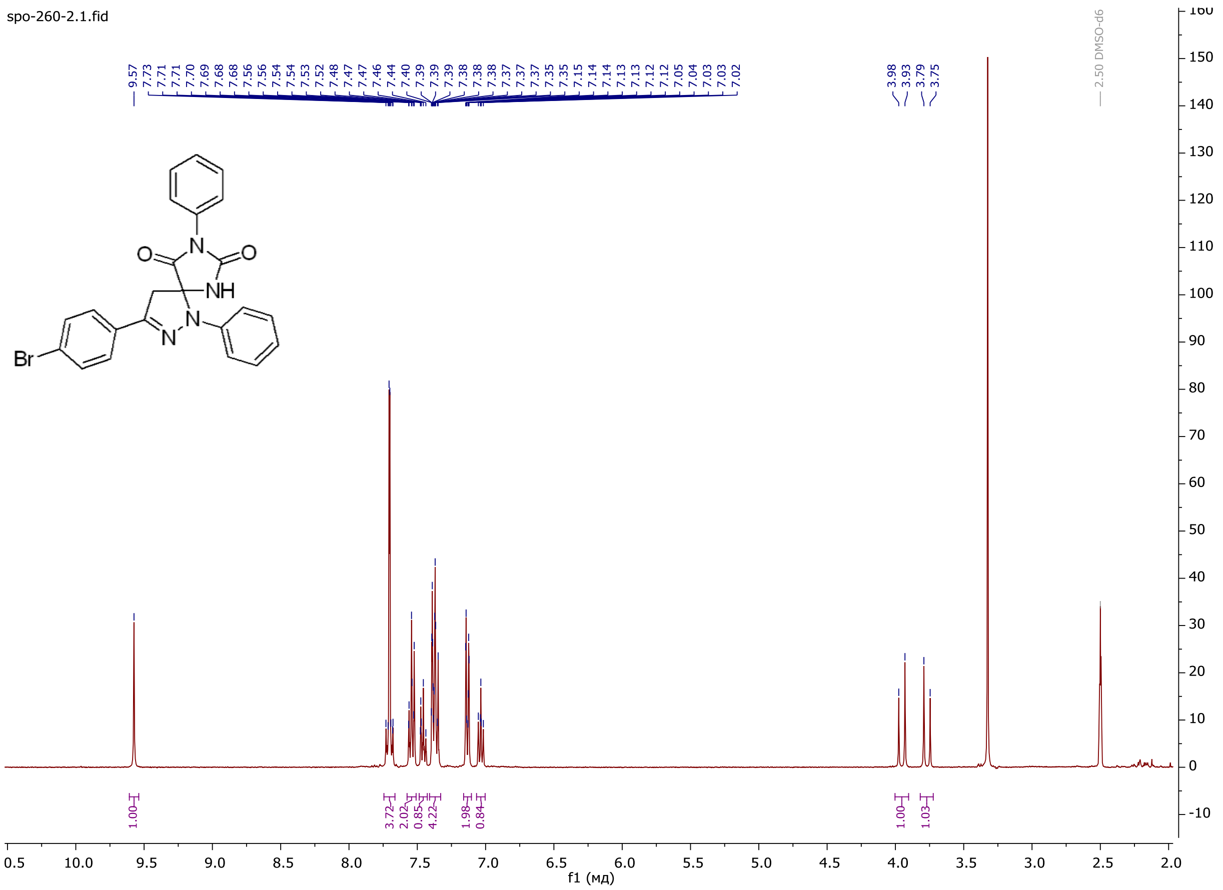

^{13}C NMR spectrum of **7b** (100 MHz, DMSO-d_6)

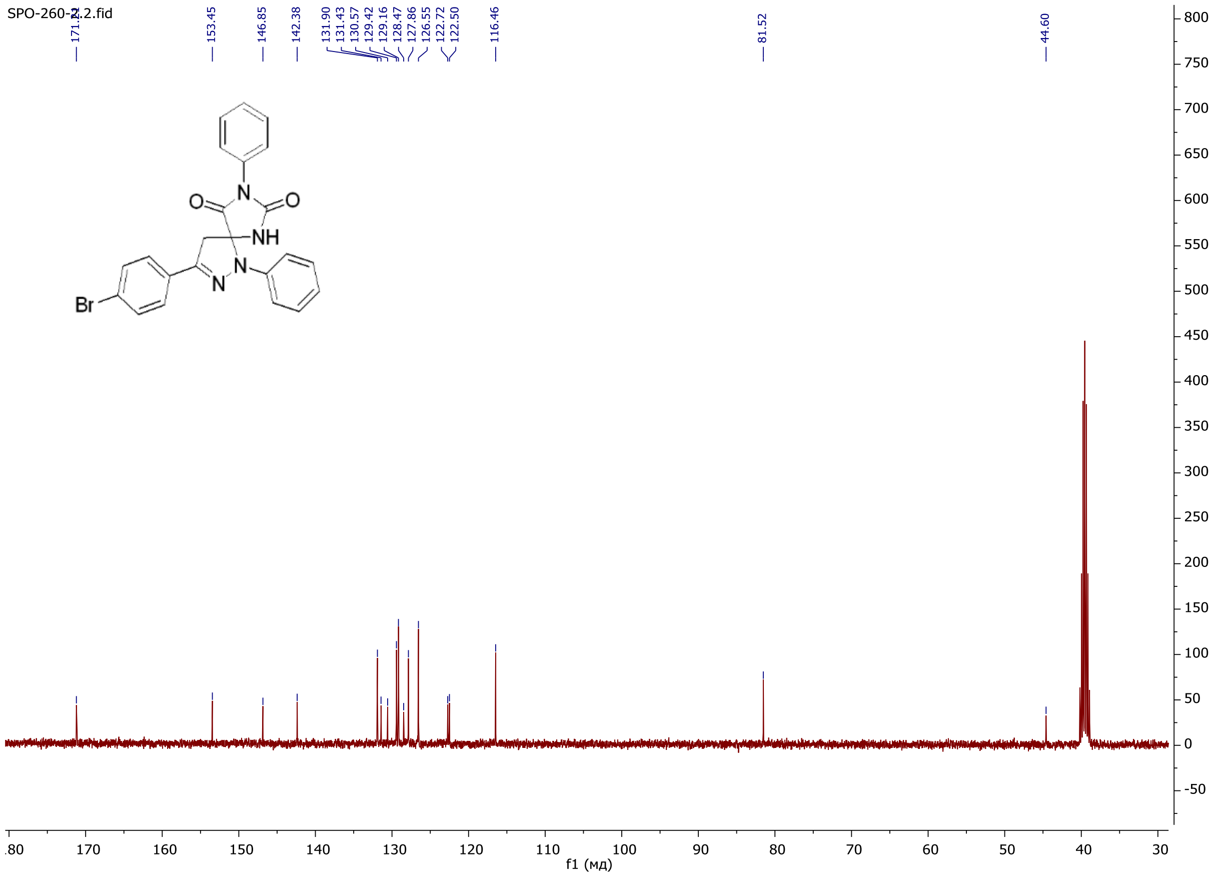

^{1}H NMR spectrum of **7c** (400 Hz, DMSO-d_6)

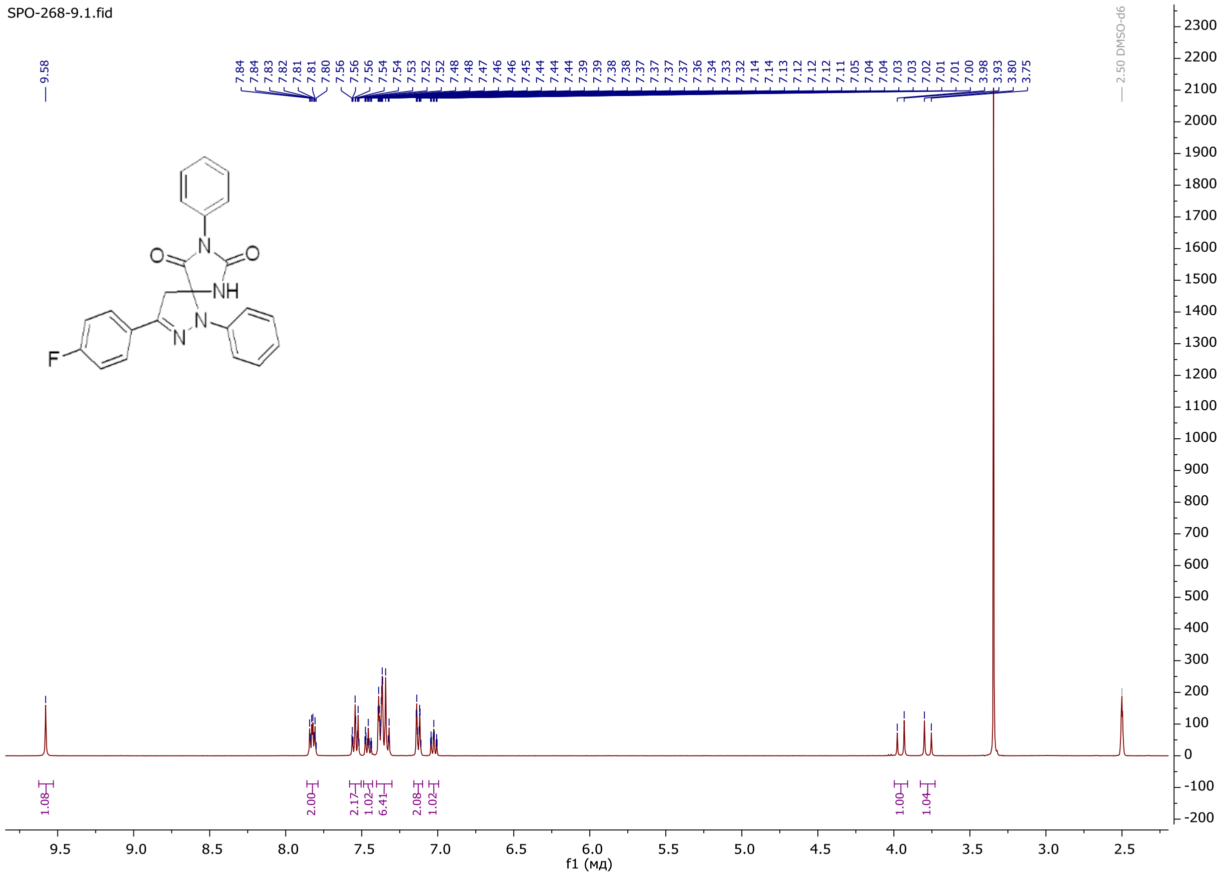

^{13}C NMR spectrum of **7c** (100 MHz, DMSO-d_6)

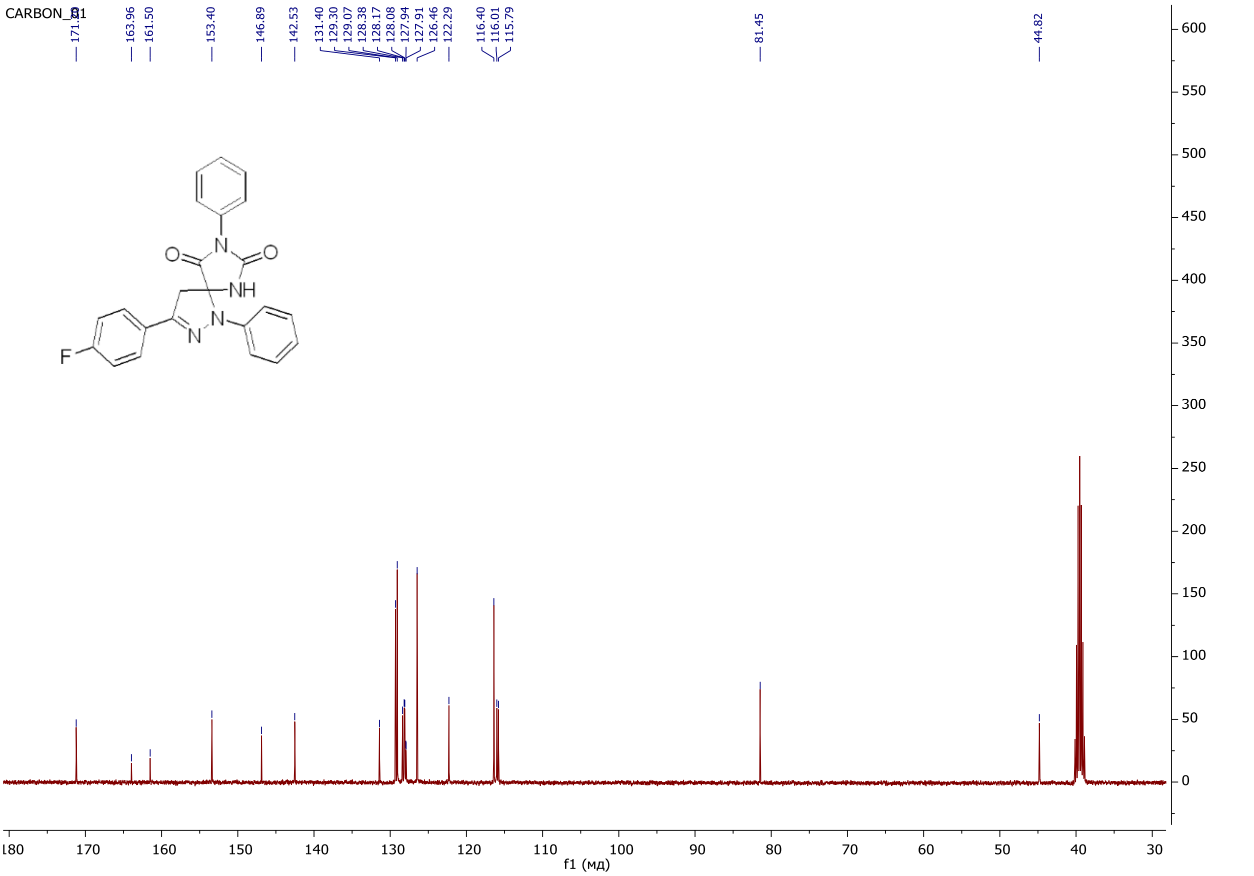

^{1}H NMR spectrum of **7d** (400 Hz, DMSO-d_6)

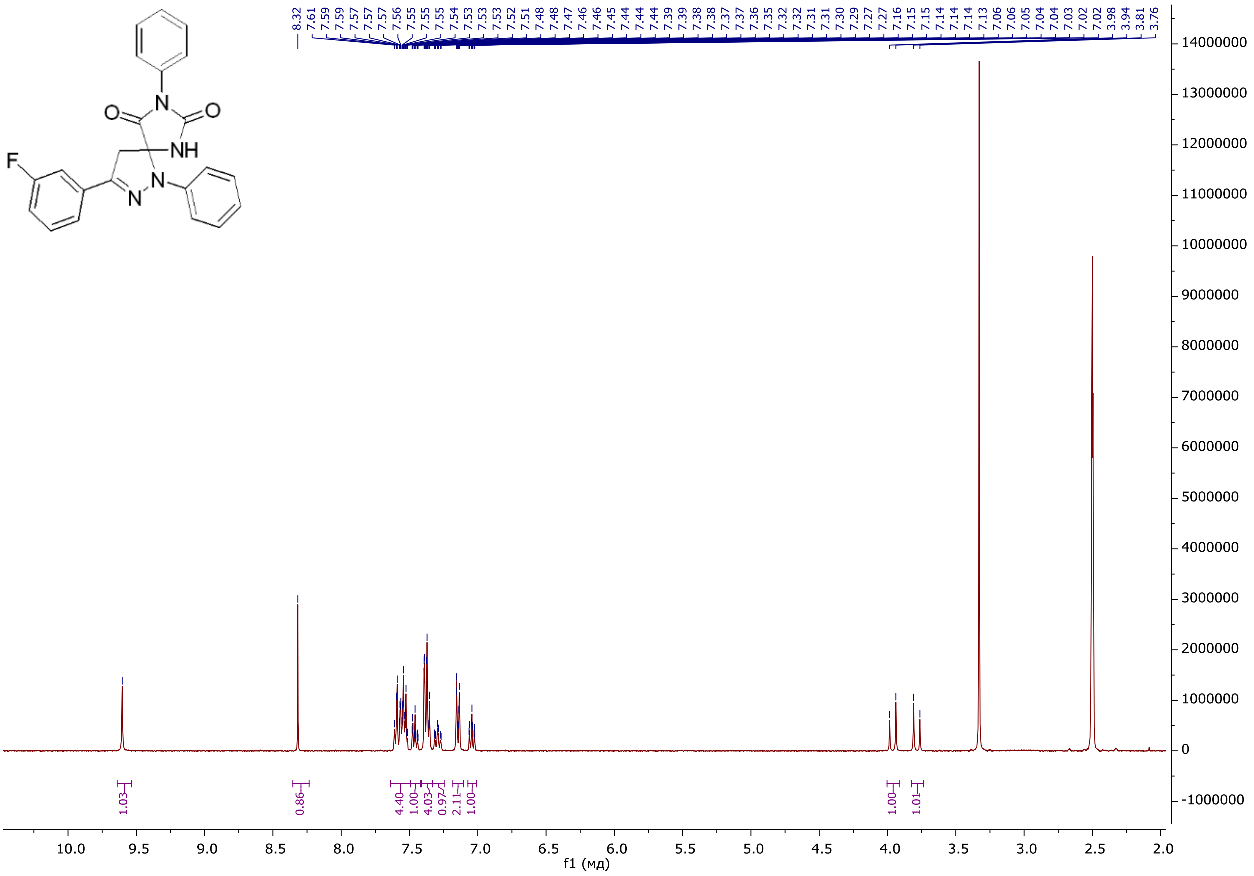

^{13}C NMR spectrum of **7d** (100 MHz, DMSO-d_6)

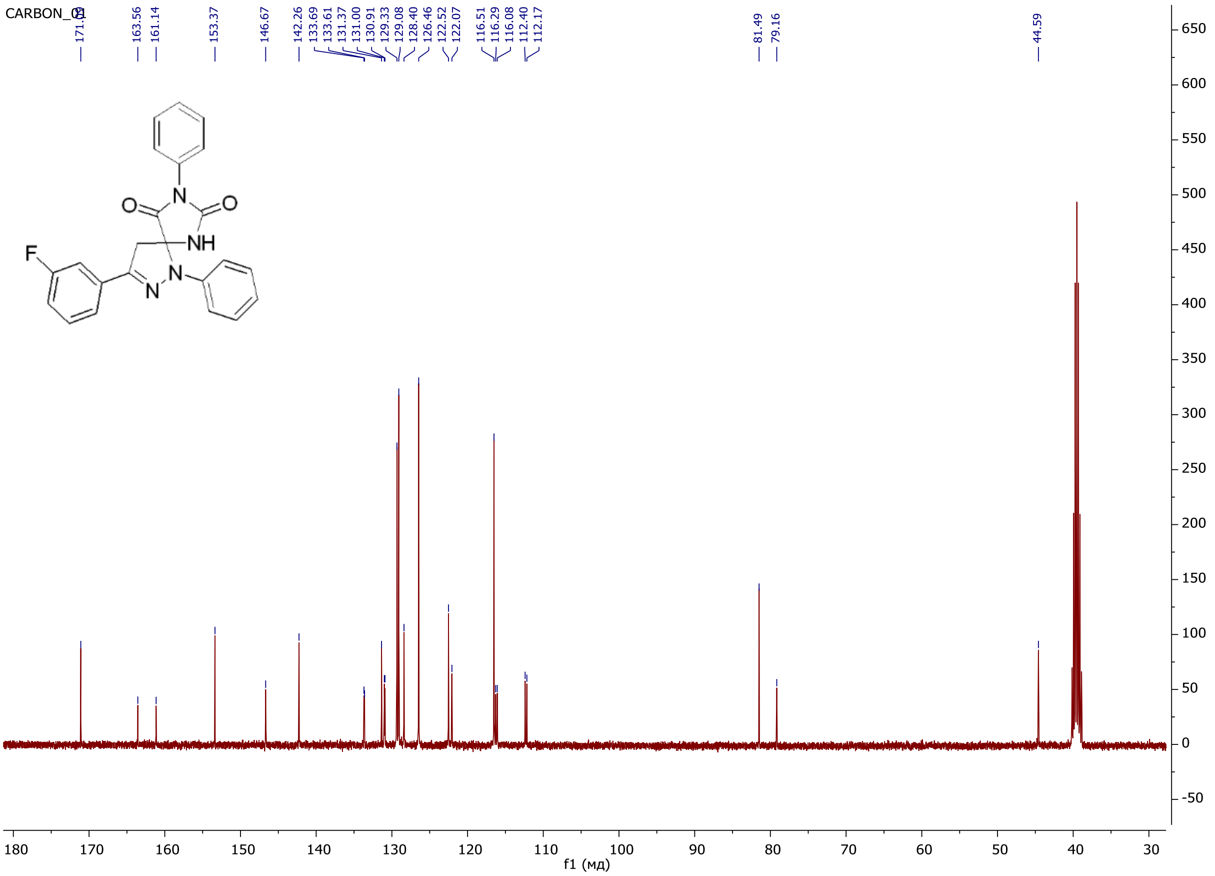

^{1}H NMR spectrum of **7e** (400 Hz, DMSO-d_6)

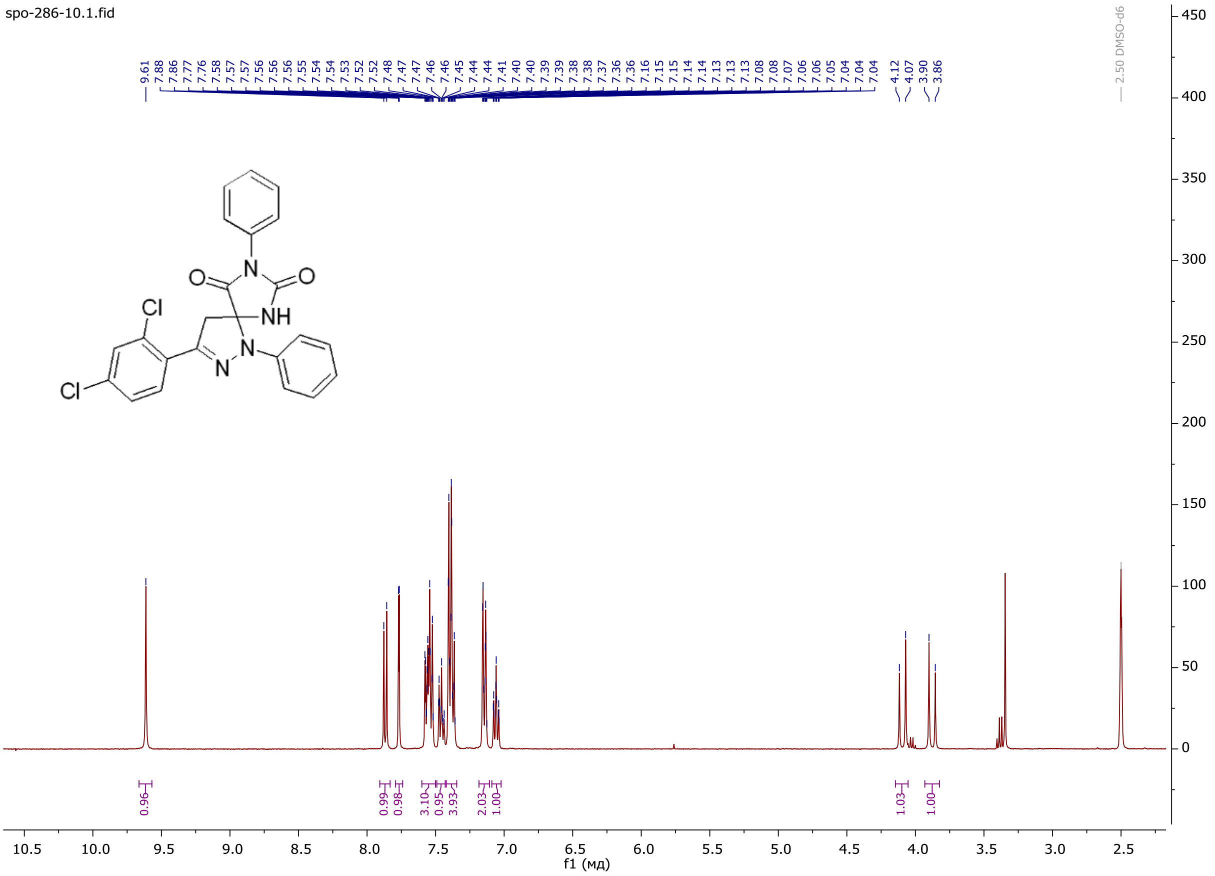

^{13}C NMR spectrum of **7e** (100 MHz, DMSO-d_6)

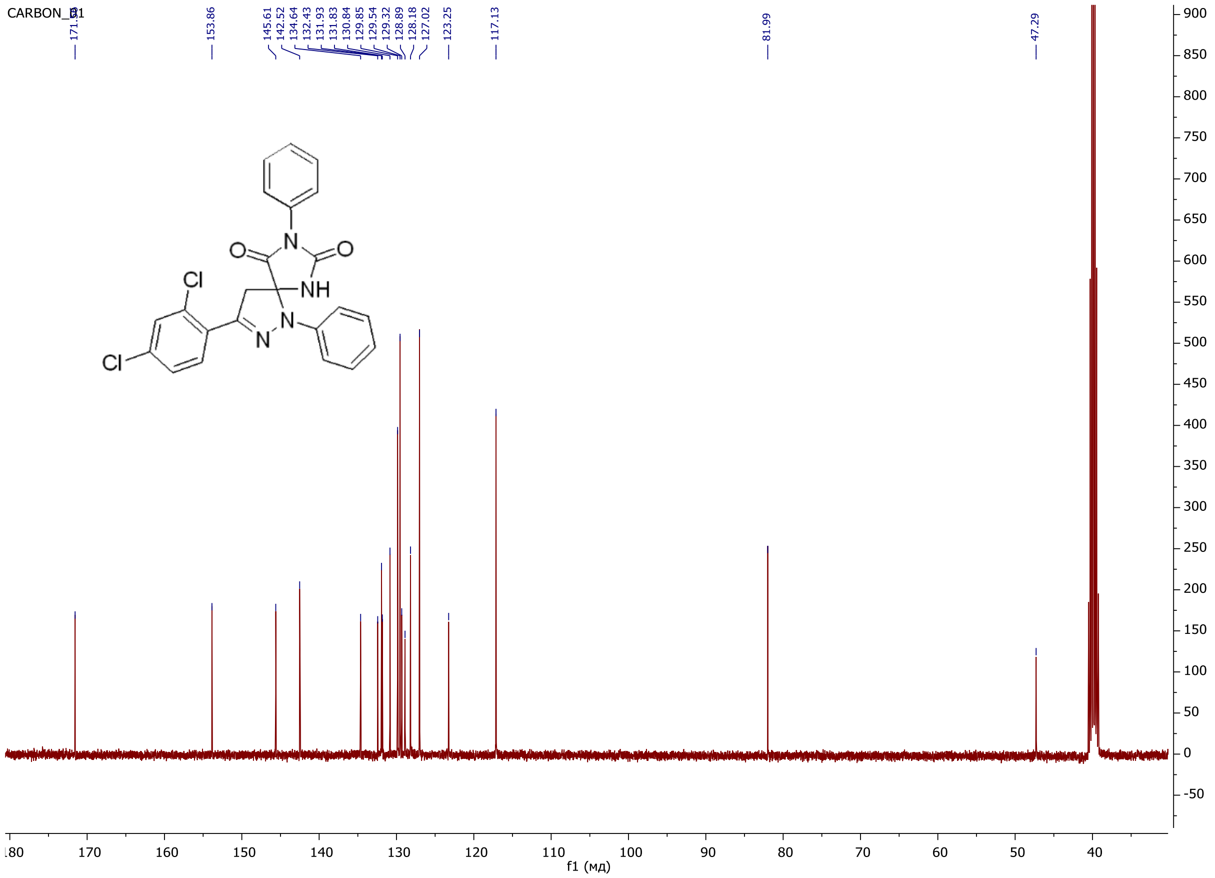

^{1}H NMR spectrum of **7f** (400 Hz, DMSO-d_6)

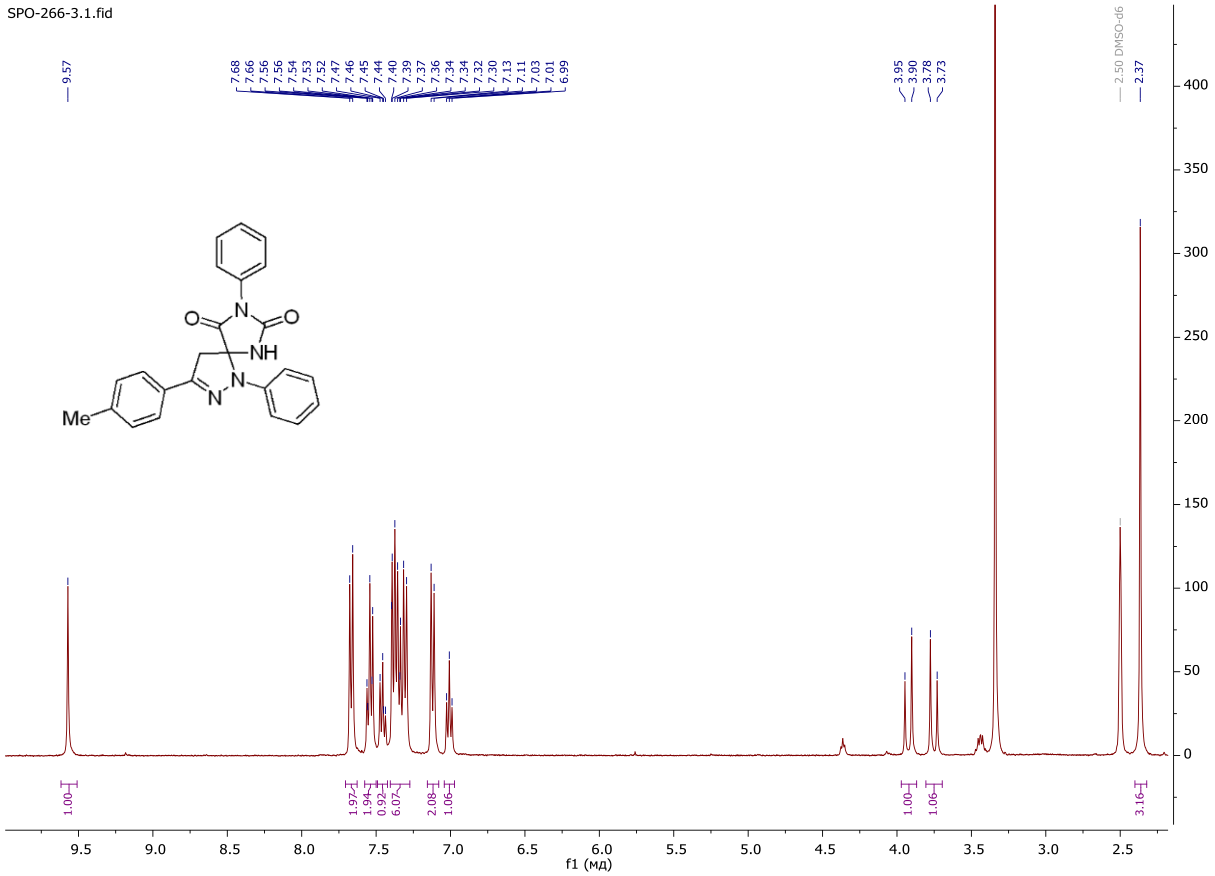

^{13}C NMR spectrum of **7f** (100 MHz, DMSO-d_6)

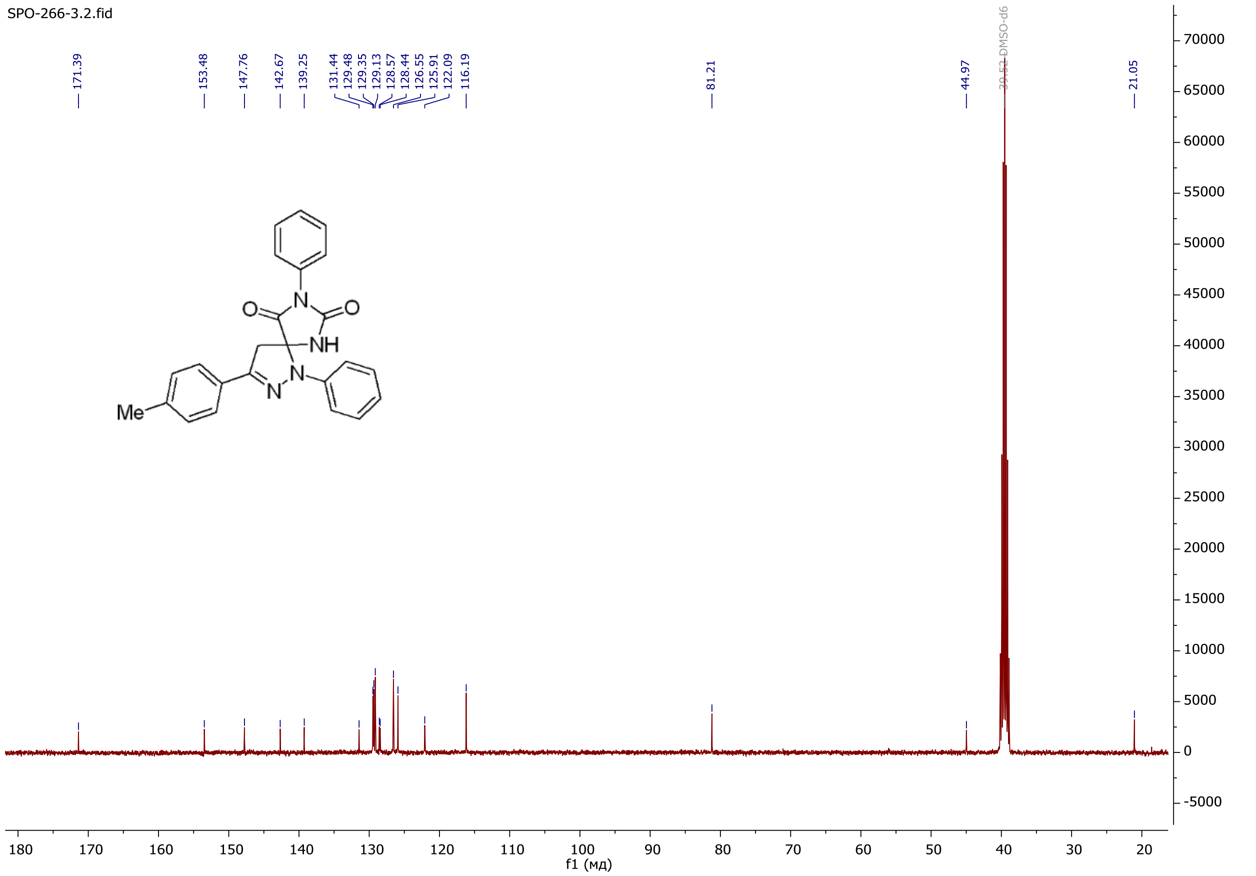

^{1}H NMR spectrum of **7g** (400 Hz, DMSO-d_6)

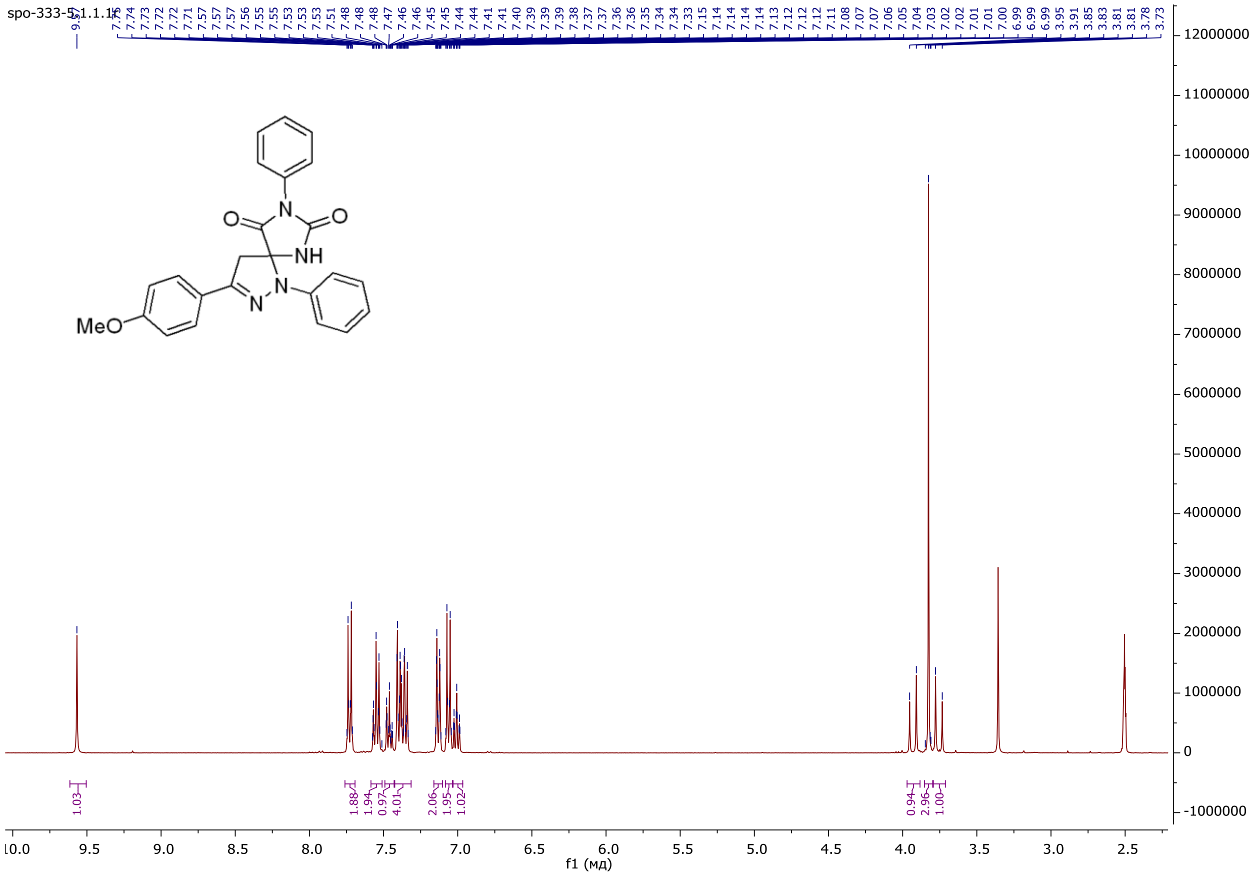

^{13}C NMR spectrum of **7g** (100 MHz, DMSO-d_6)

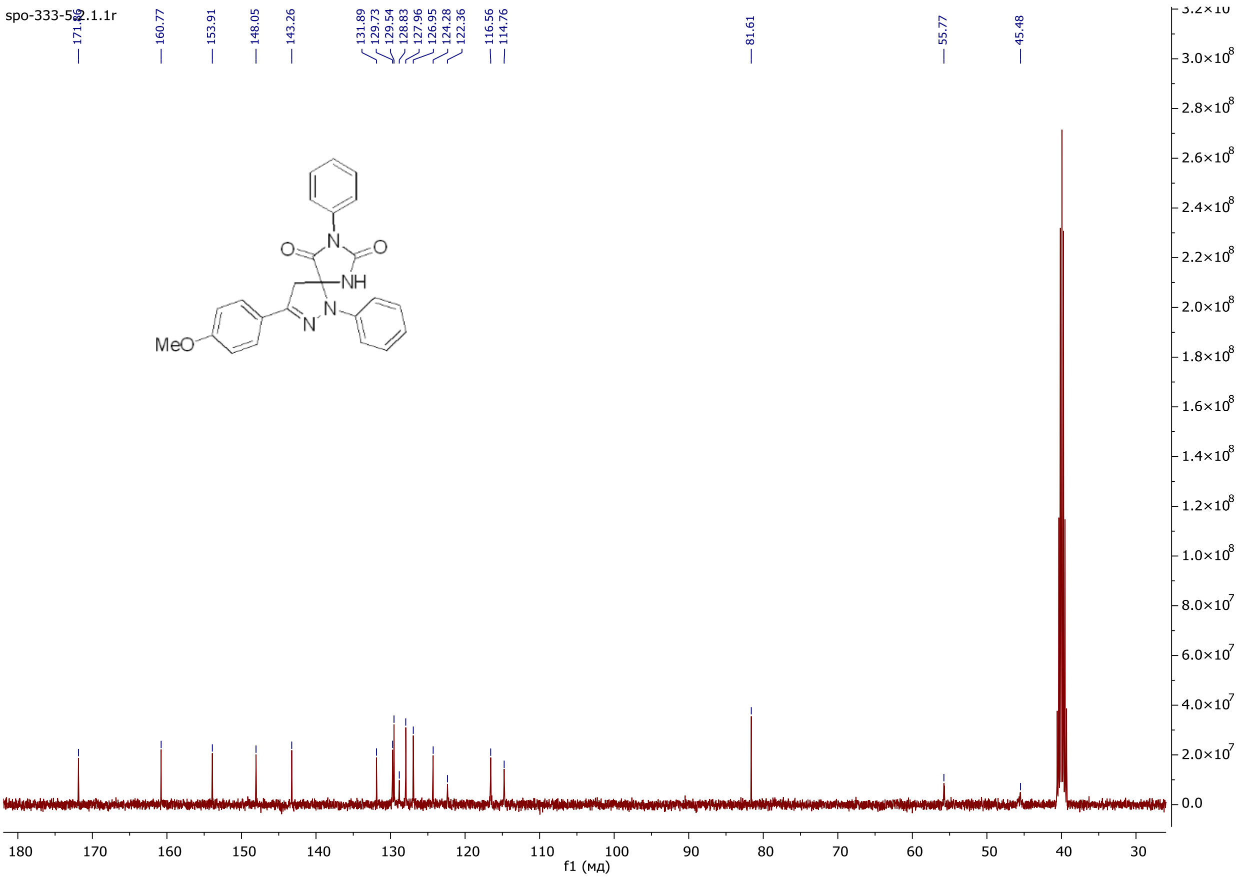

^{1}H NMR spectrum of **7h** (400 Hz, DMSO-d_6)

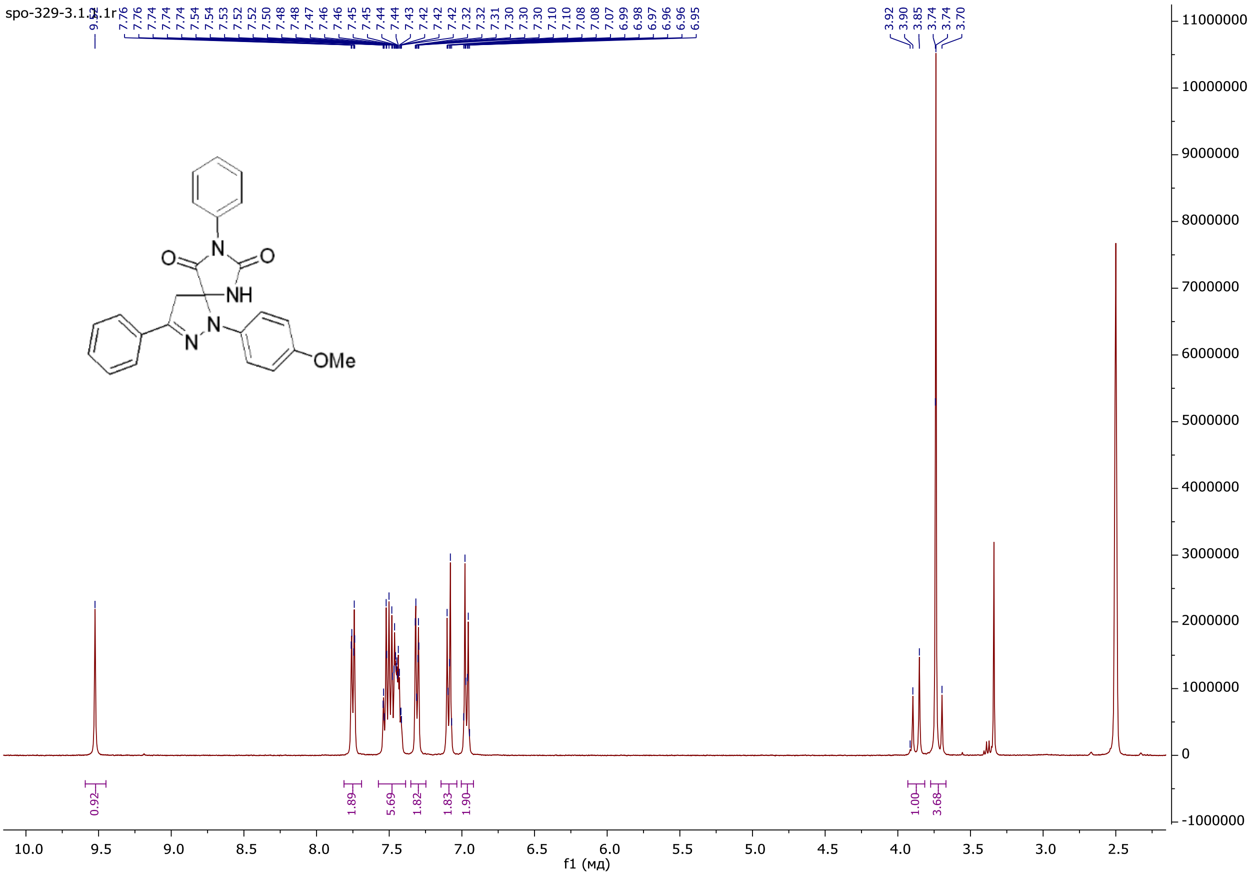

^{13}C NMR spectrum of **7h** (100 MHz, DMSO-d_6)

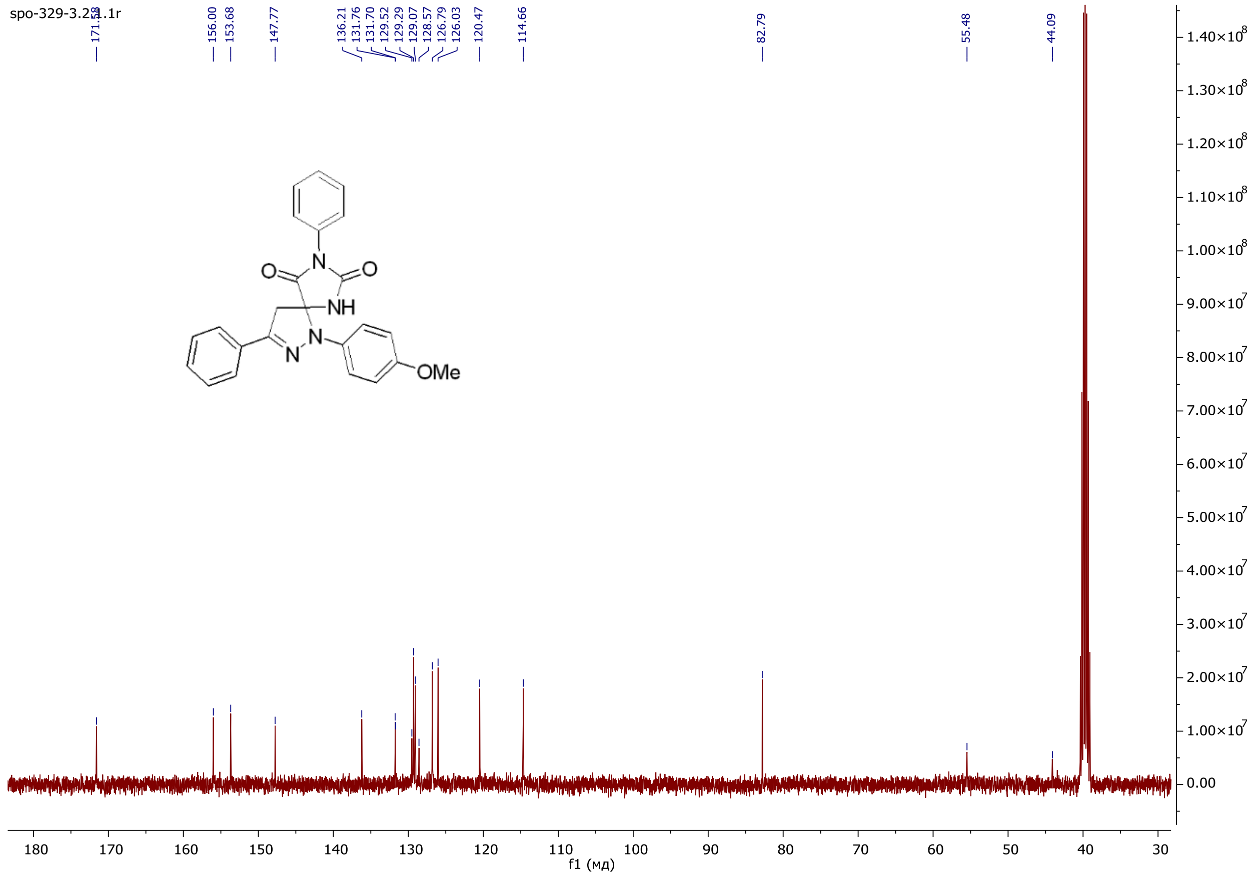

^{1}H NMR spectrum of **7i** (400 Hz, DMSO-d_6)

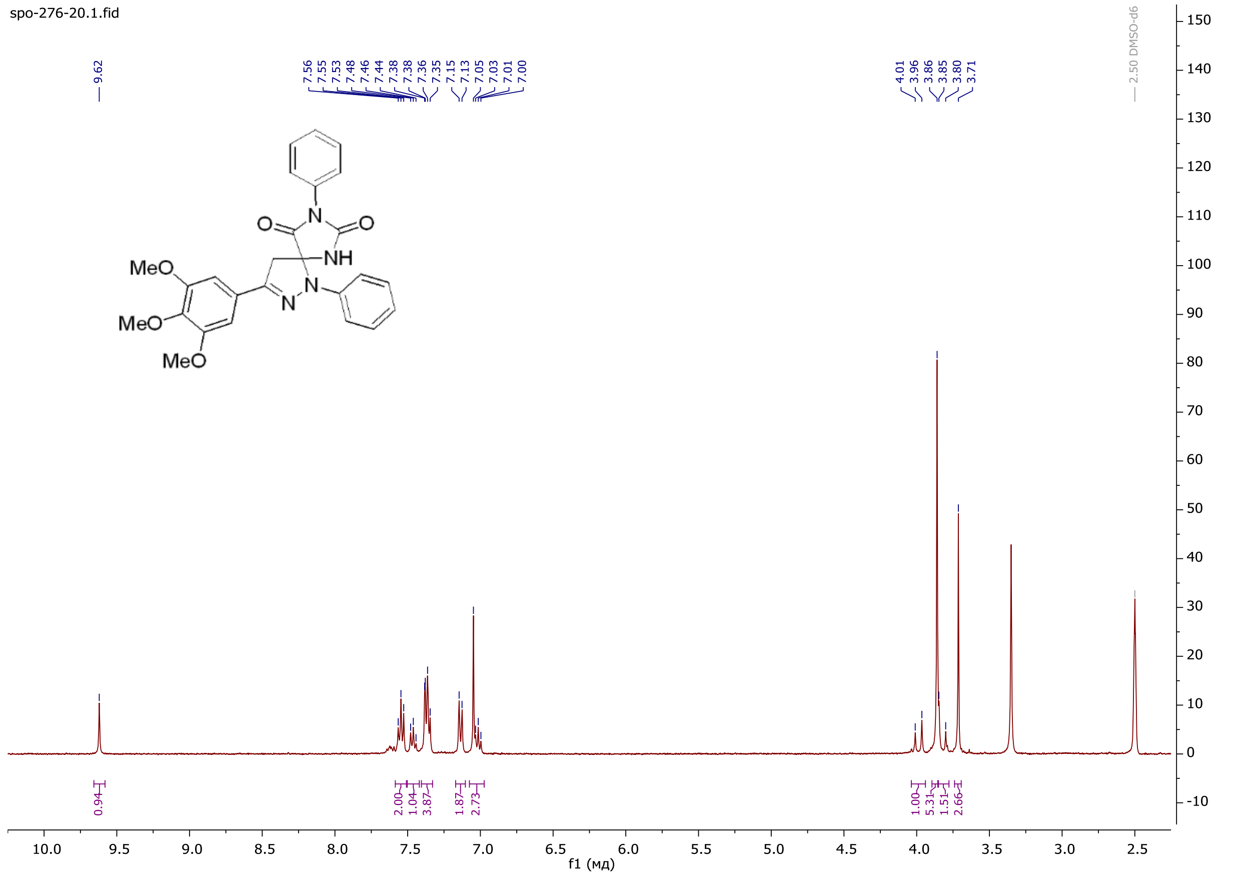

^{13}C NMR spectrum of **7i** (100 MHz, DMSO-d_6)

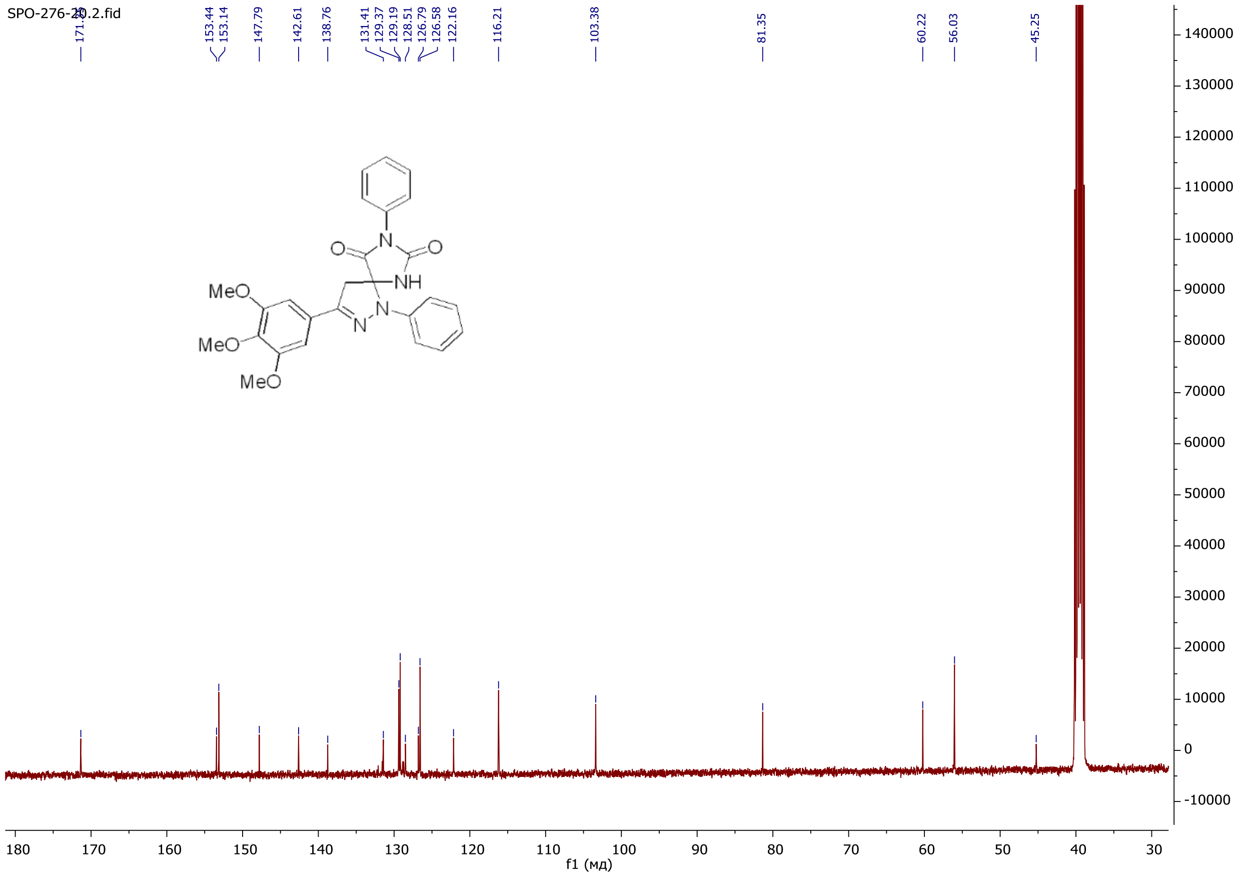

^{1}H NMR spectrum of **7j** (400 Hz, DMSO-d_6)

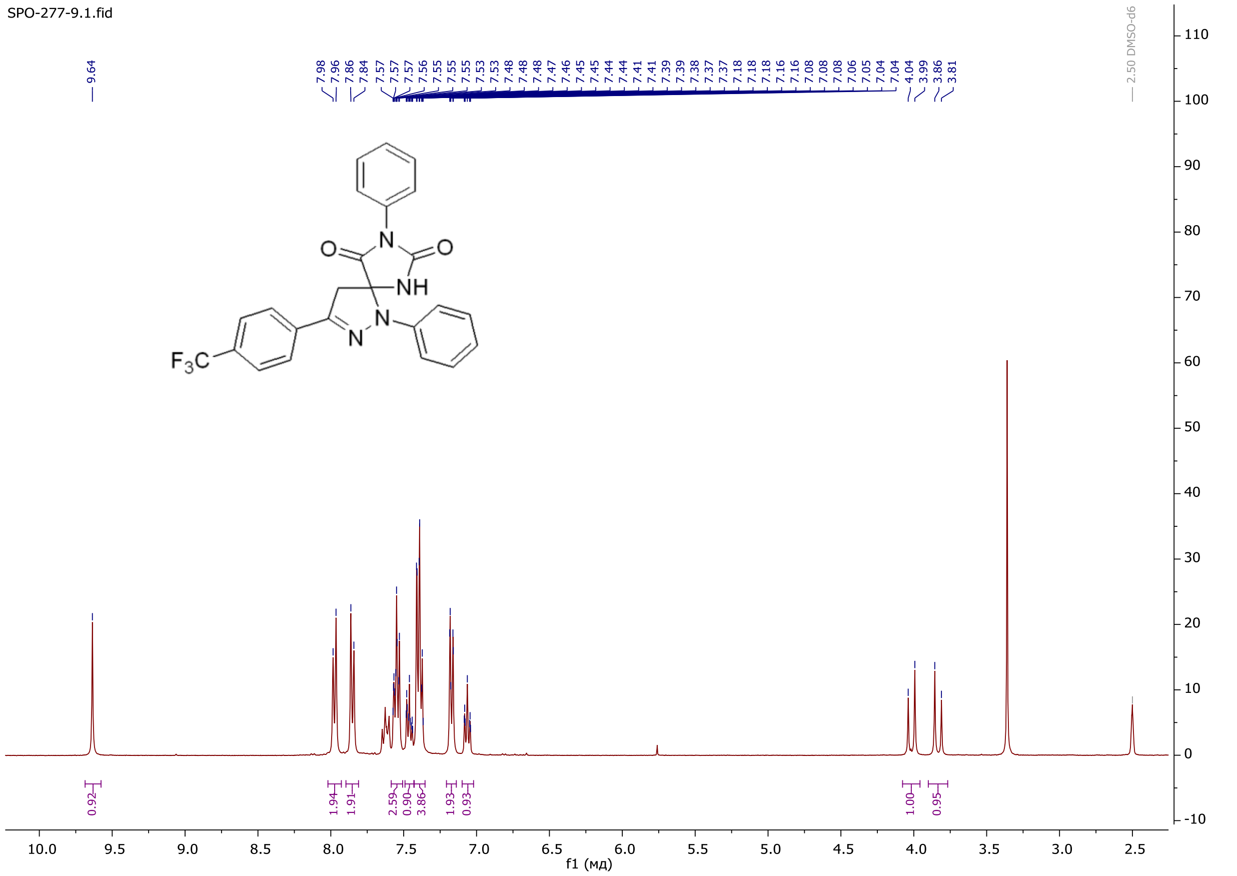

^{13}C NMR spectrum of **7j** (100 MHz, DMSO-d_6)

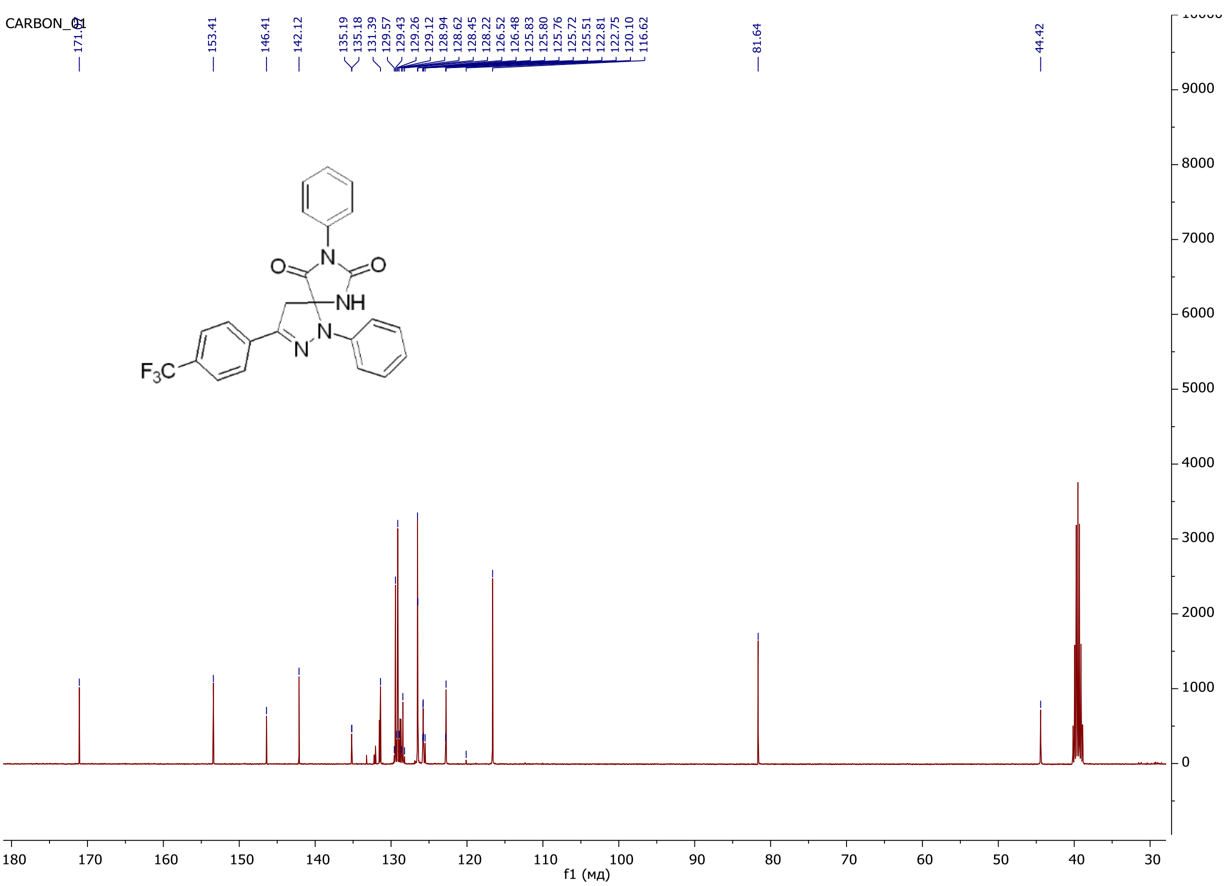

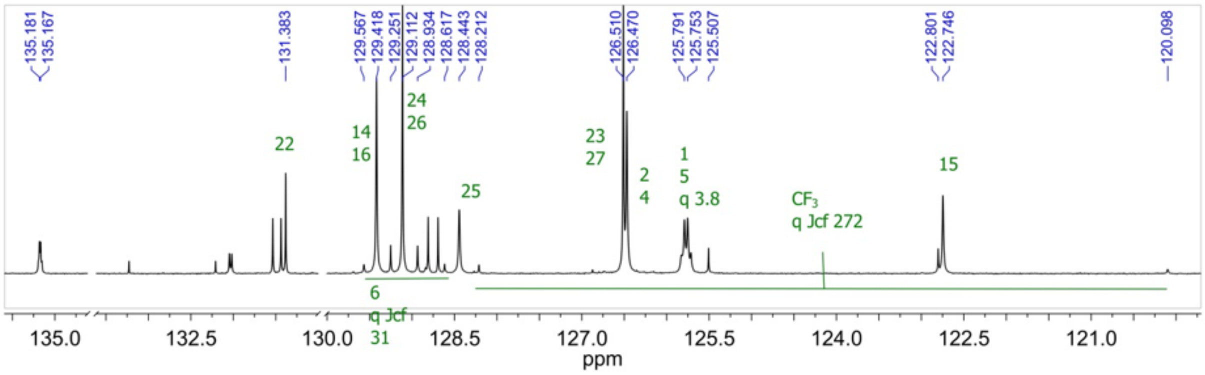

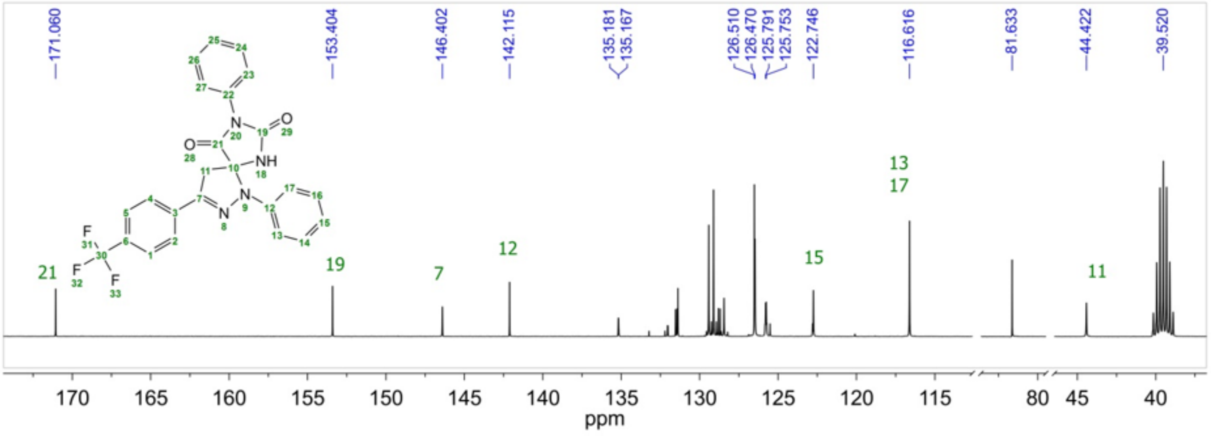

HSQC ^{1}H-^{13}C NMR spectra of compound **7j**

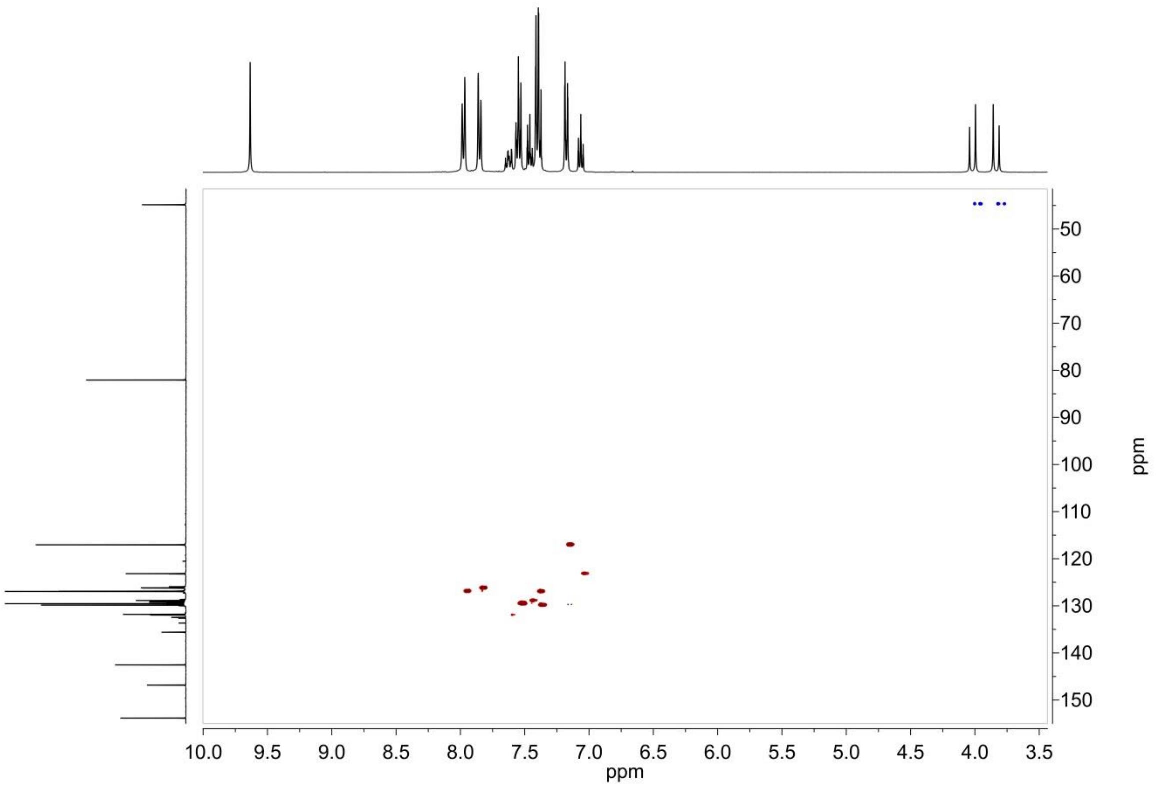

HMBC ^{1}H-^{13}C NMR spectra of compound **7j**

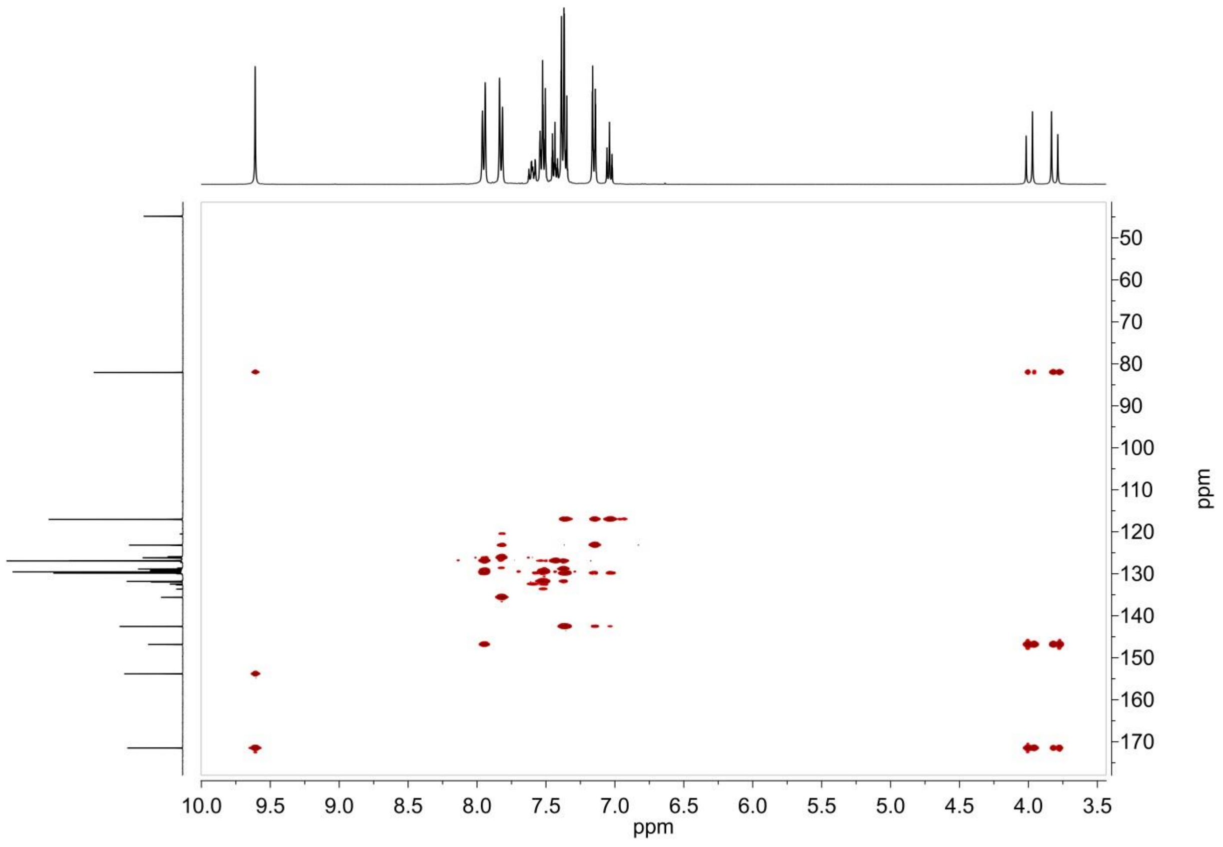

NOESY 1D NMR spectra of compound **7j**

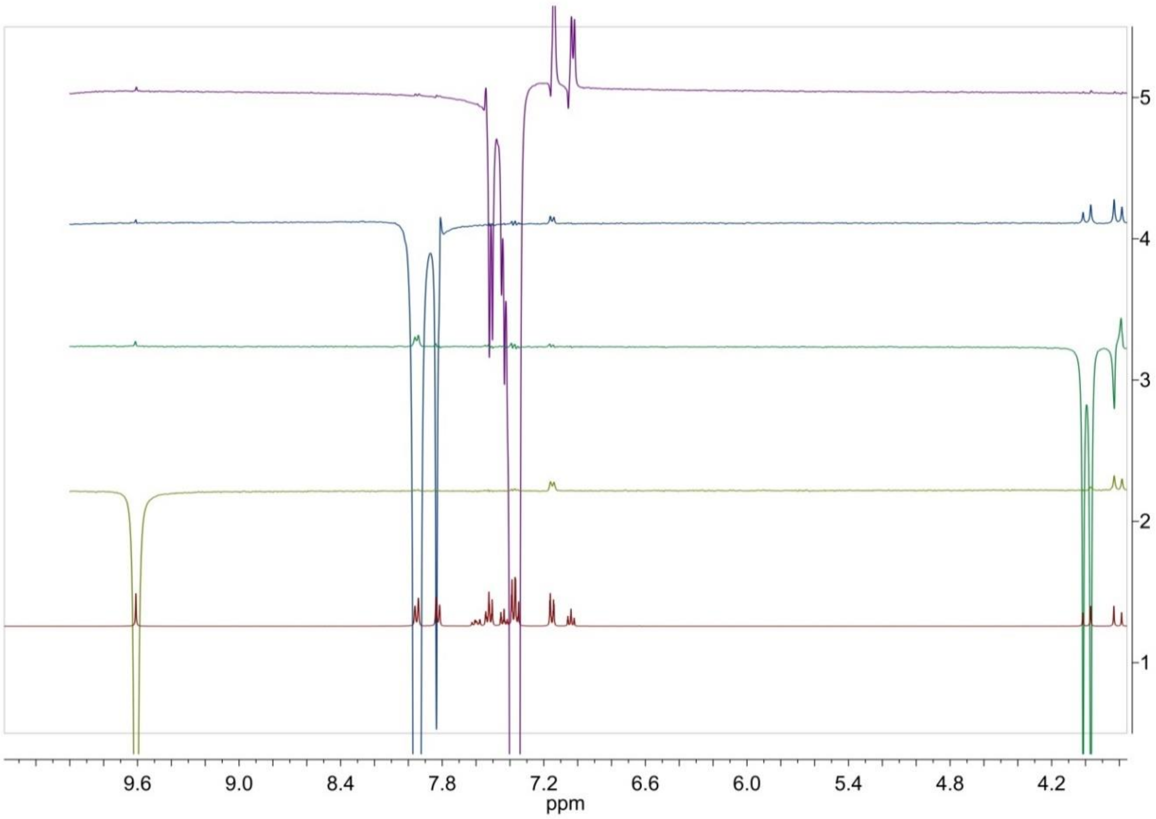

ROESY NMR spectra of compound **7j**

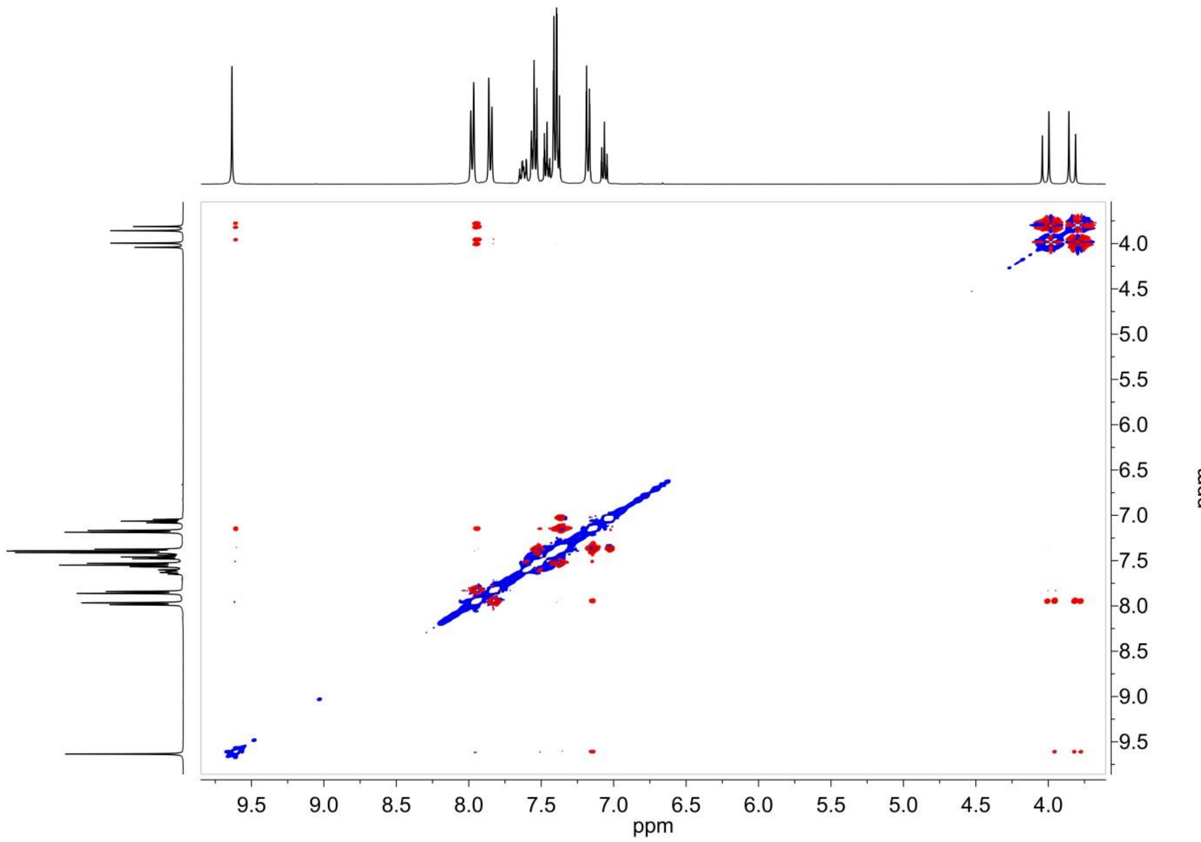

TOSCY 1D NMR spectra of compound **7j**

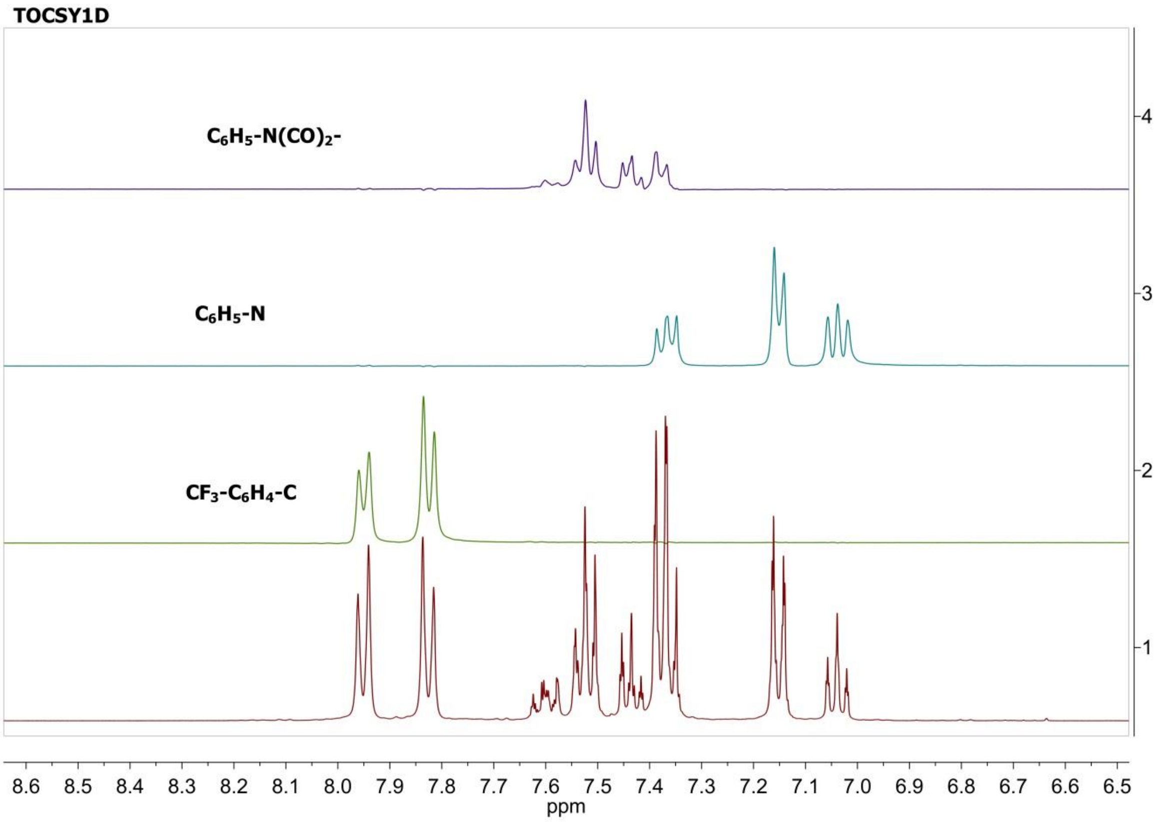

^{1}H NMR spectrum of **7k** (400 Hz, DMSO-d_6)

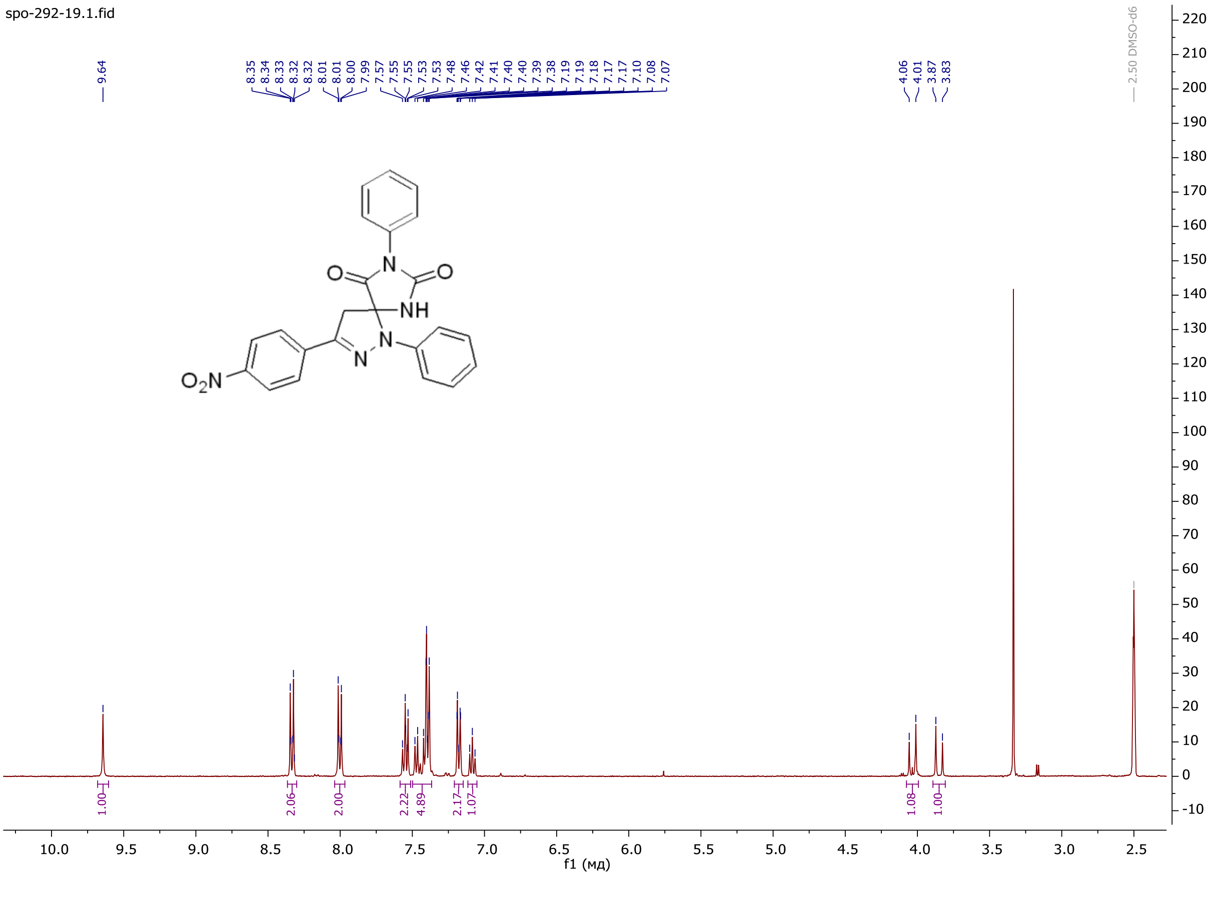

^{13}C NMR spectrum of **7k** (100 MHz, DMSO-d_6)

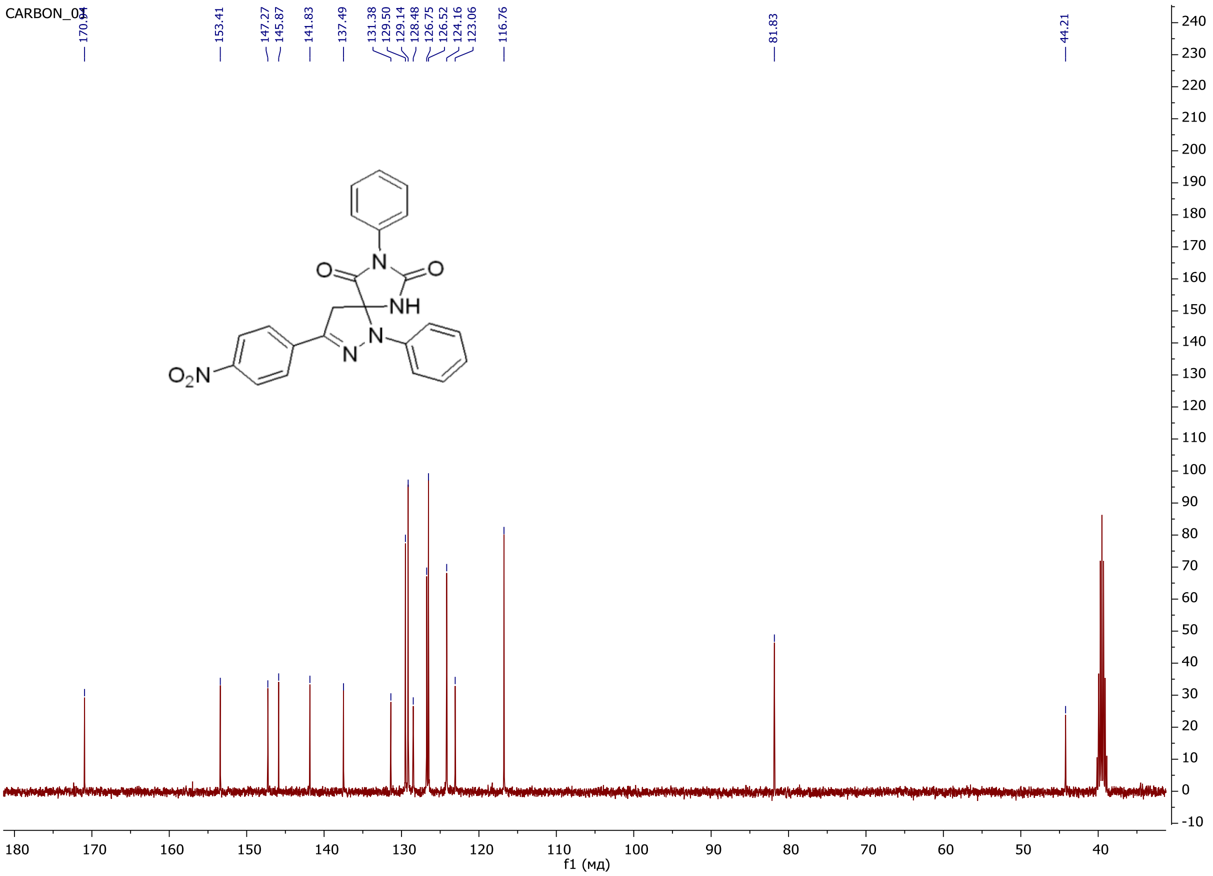

HSQC ^{1}H-^{13}C NMR spectra of compound **7k**

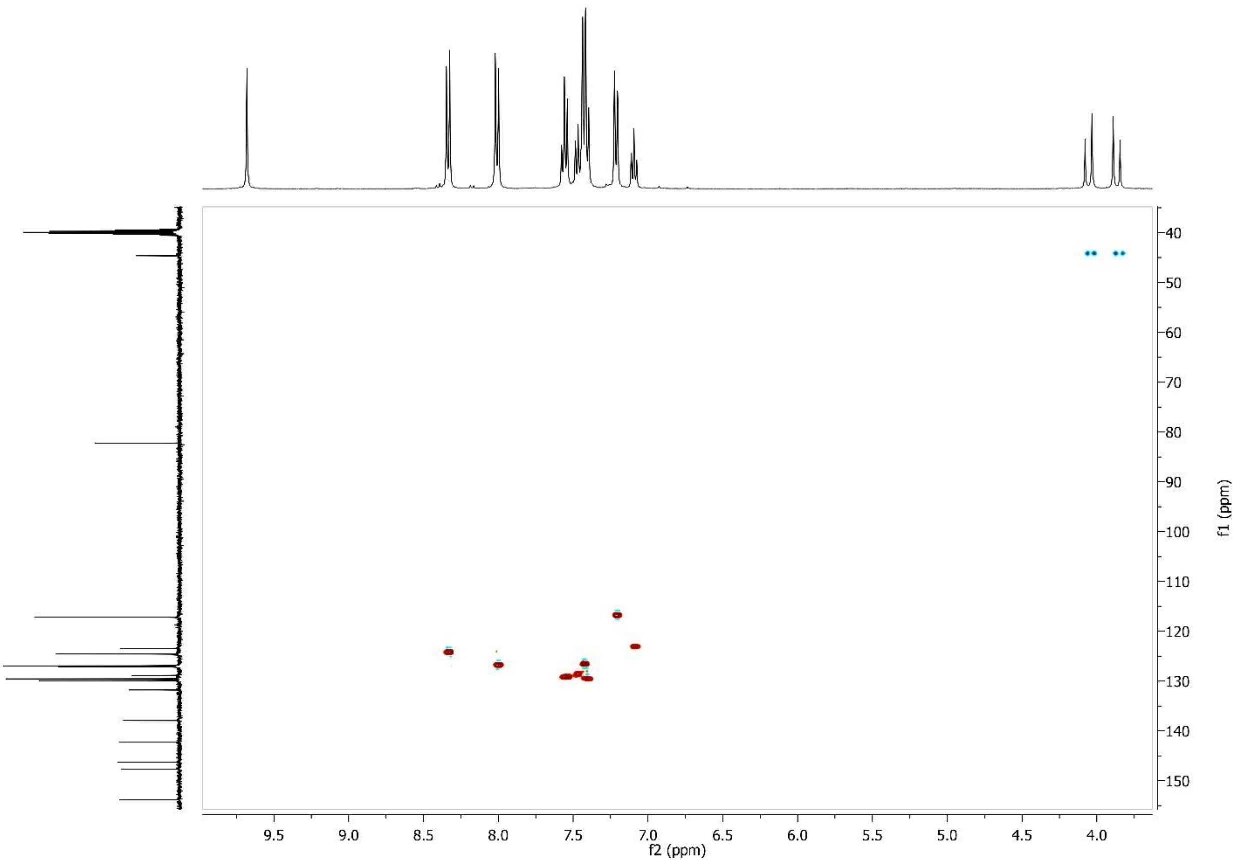

HMBC ^{1}H-^{13}C NMR spectra of compound **7k**

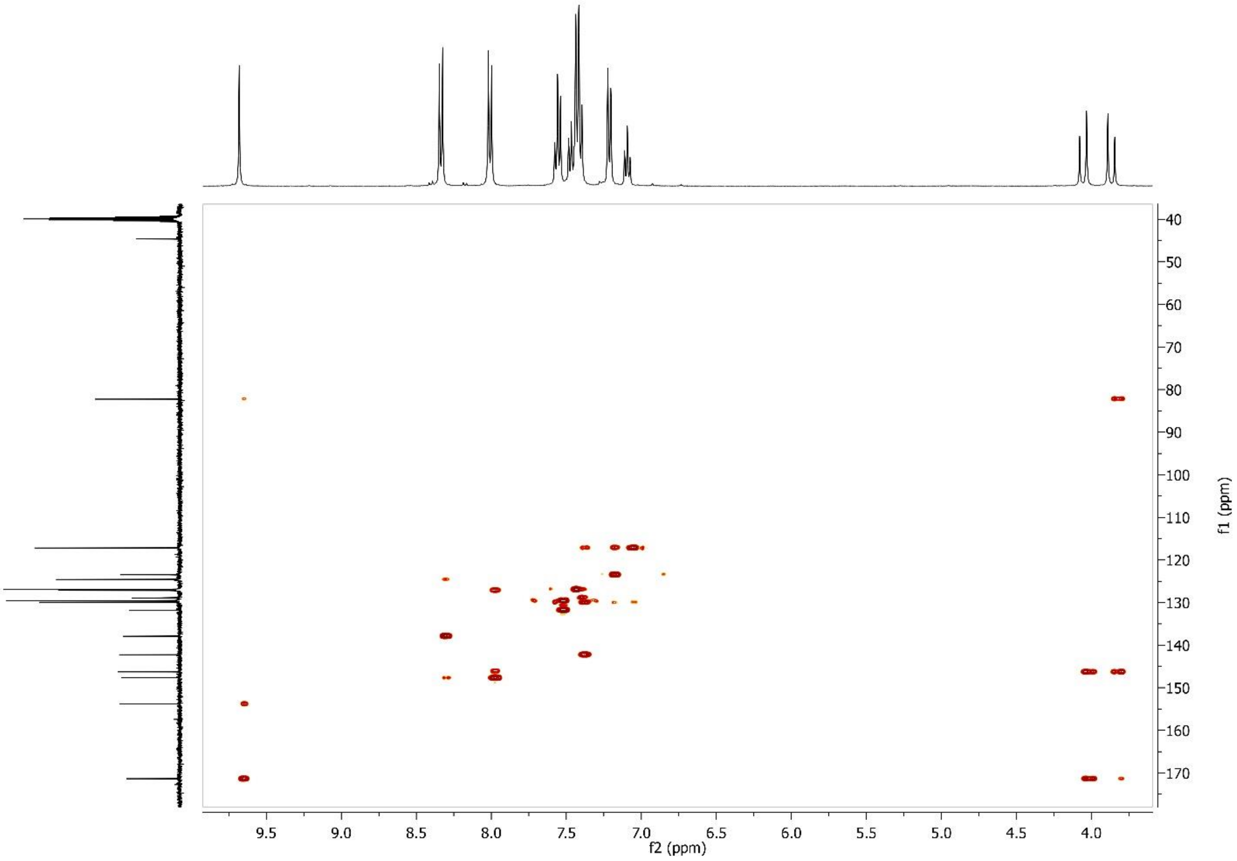

NOESY 1D NMR spectra of compound **7k**

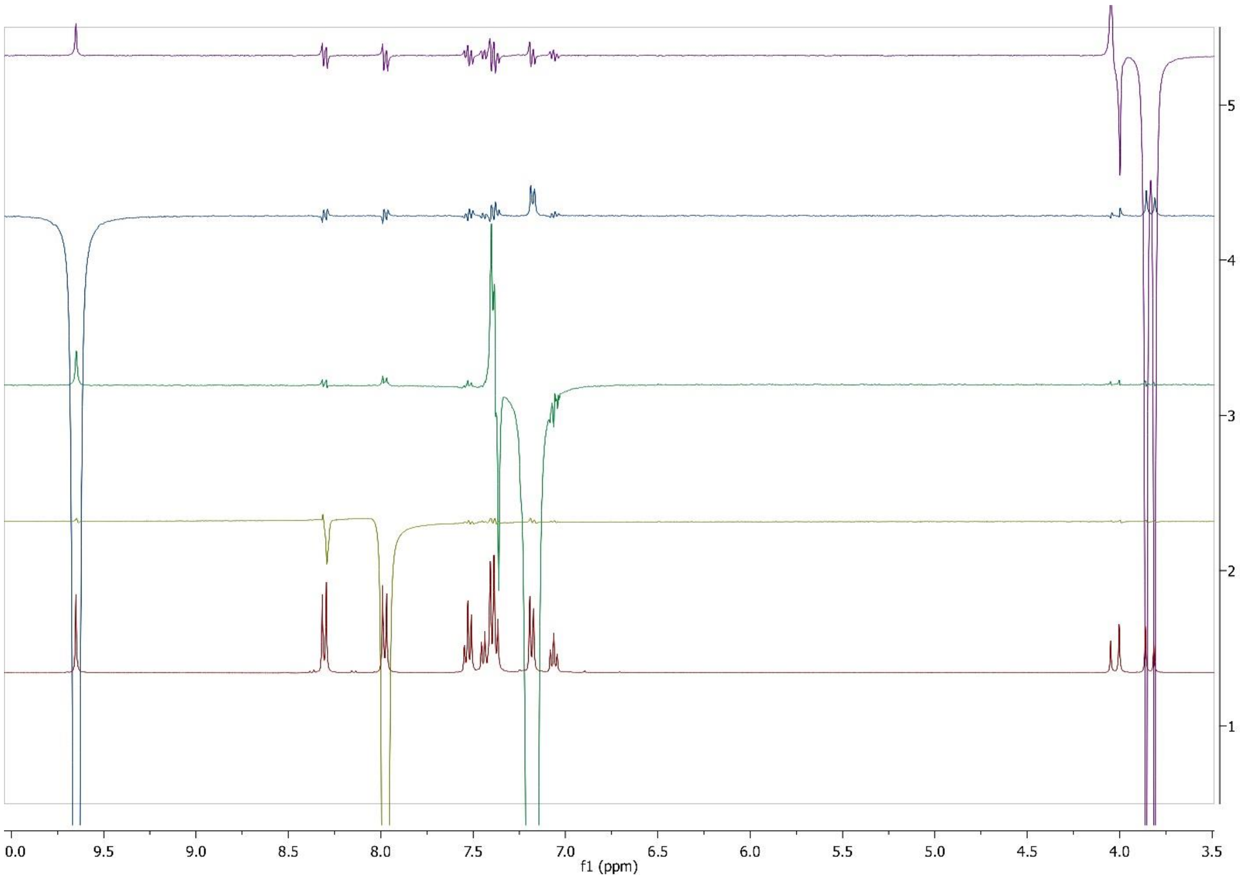

^{1}H NMR spectrum of **7l** (400 Hz, DMSO-d_6)

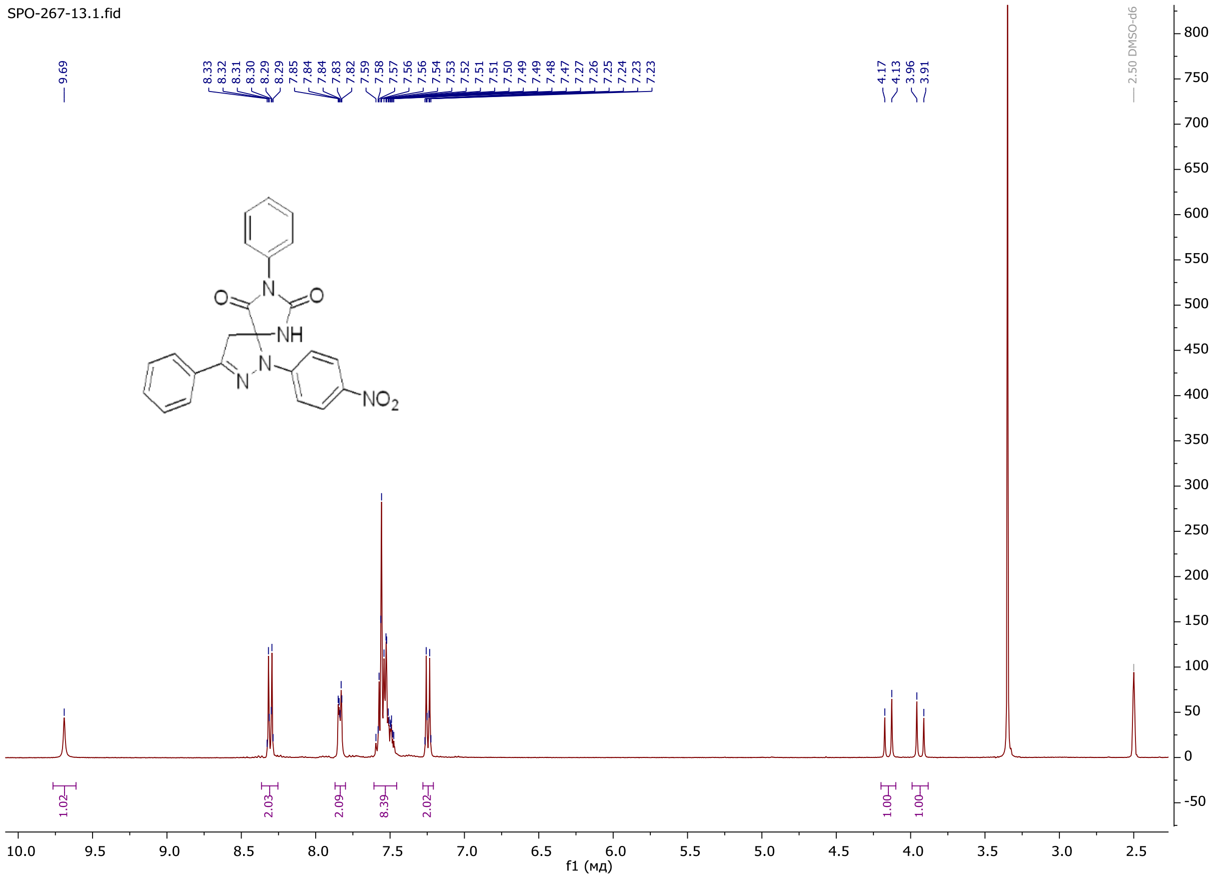

^{13}C NMR spectrum of **7l** (100 MHz, DMSO-d_6)

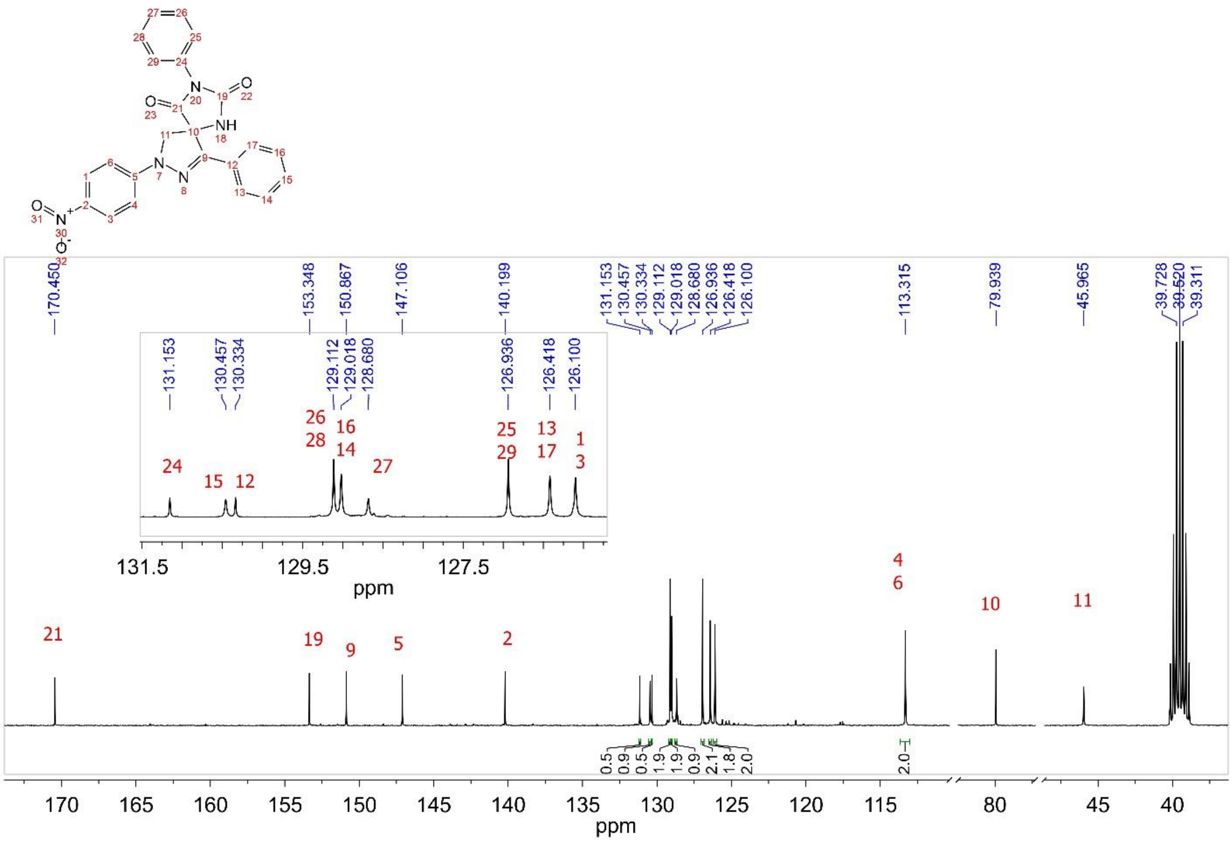

HSQC ^{1}H-^{13}C NMR spectra of compound **7l**

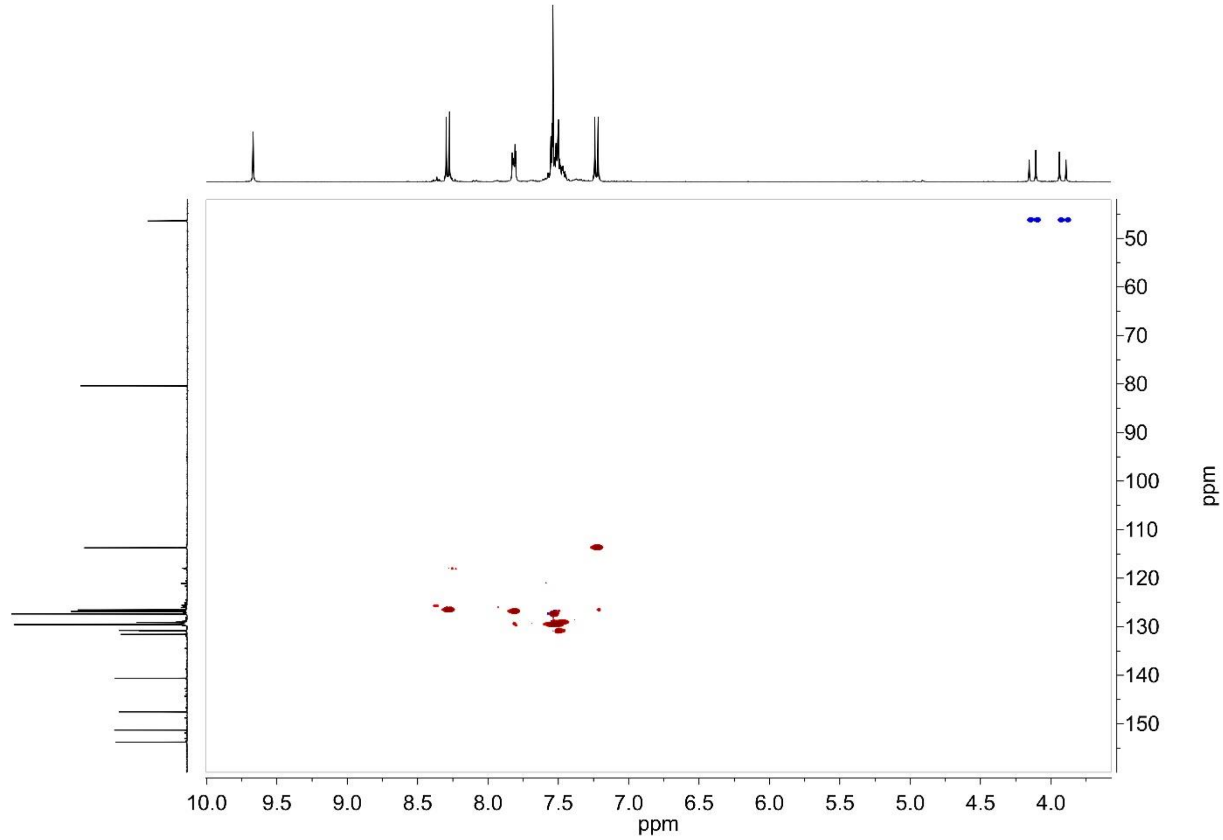

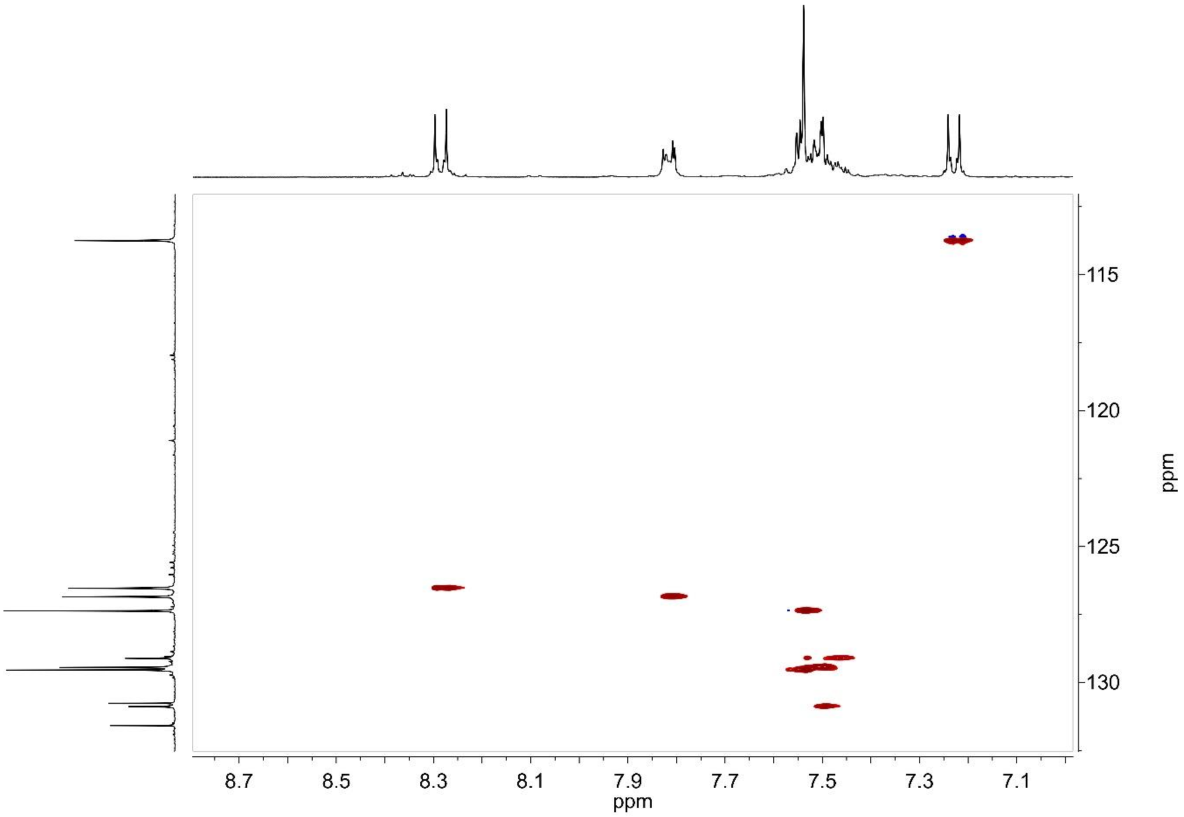

HMBC ^{1}H-^{13}C NMR spectra of compound **7l**

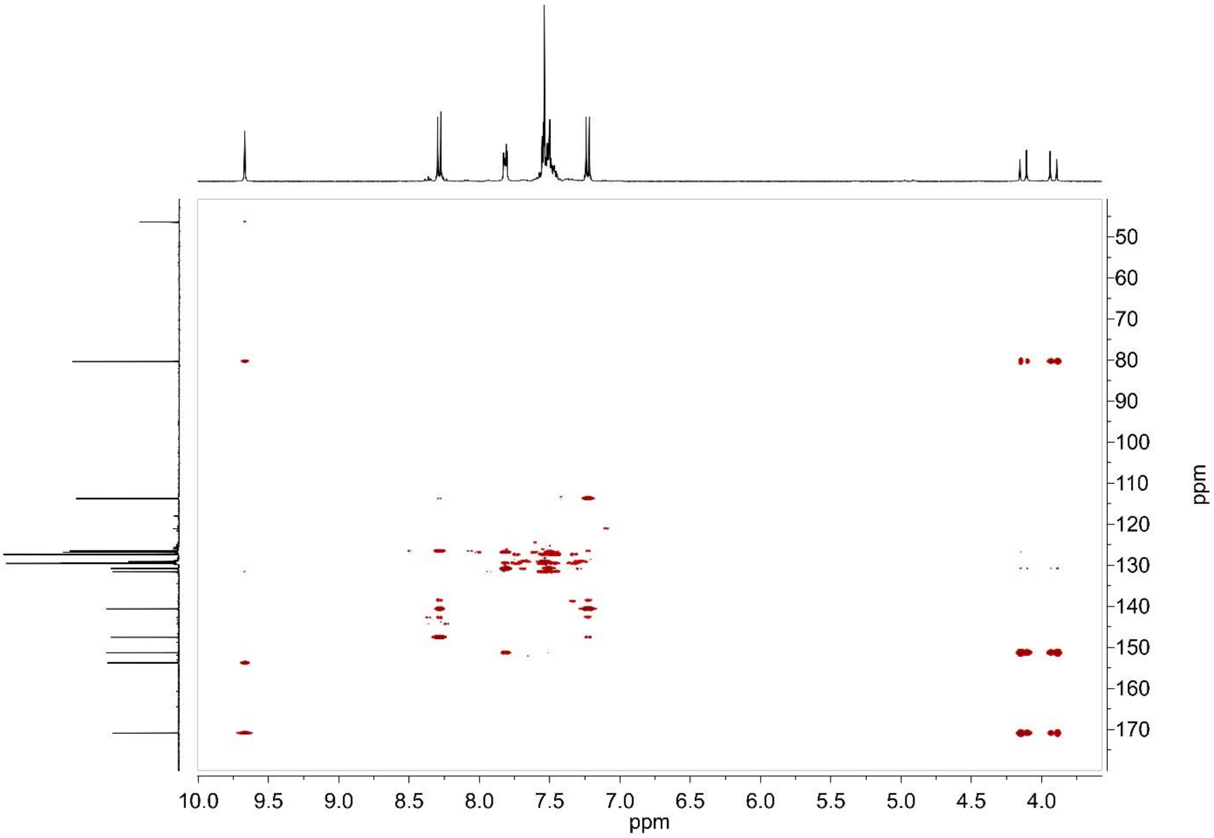

^{1}H NMR spectrum of **9a** (400 Hz, DMSO-d_6)

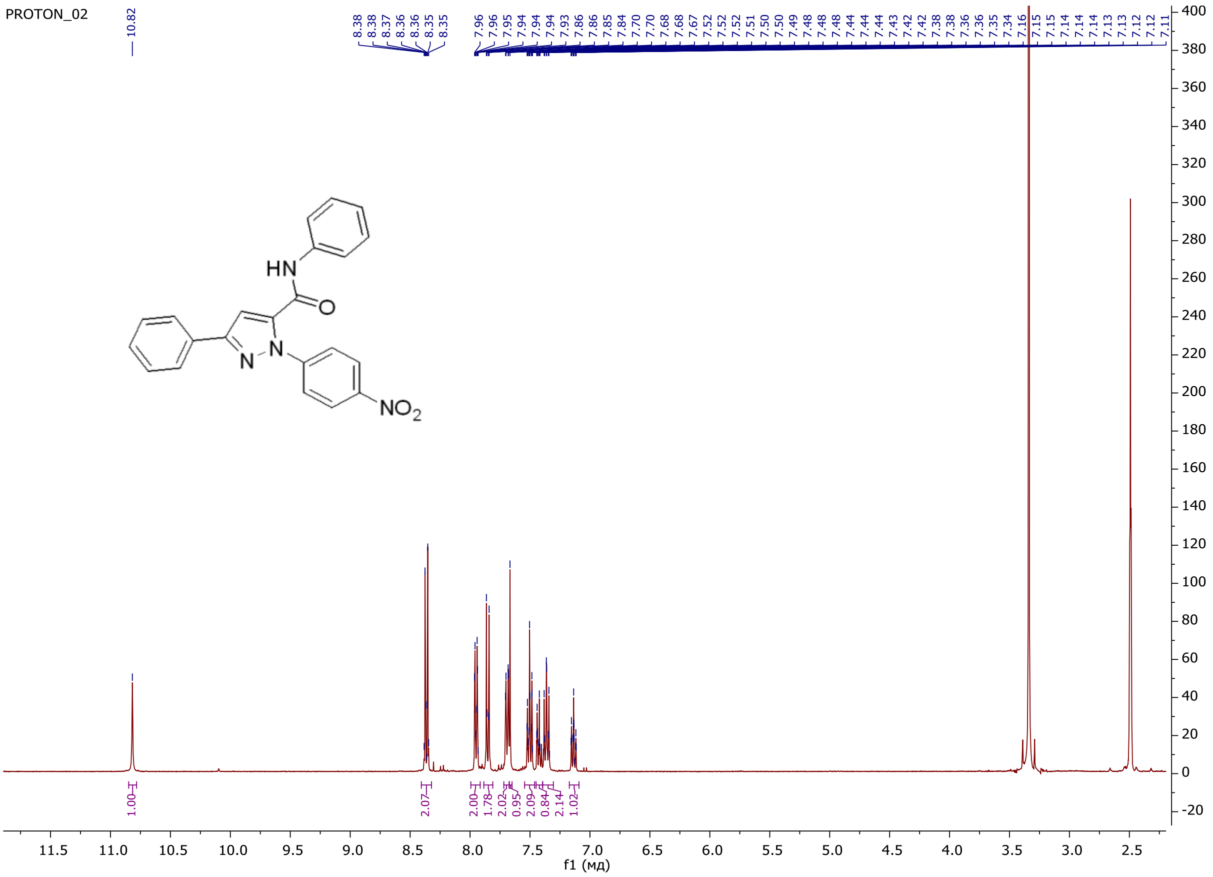

^{13}C NMR spectrum of **9a** (100 MHz, DMSO-d_6)

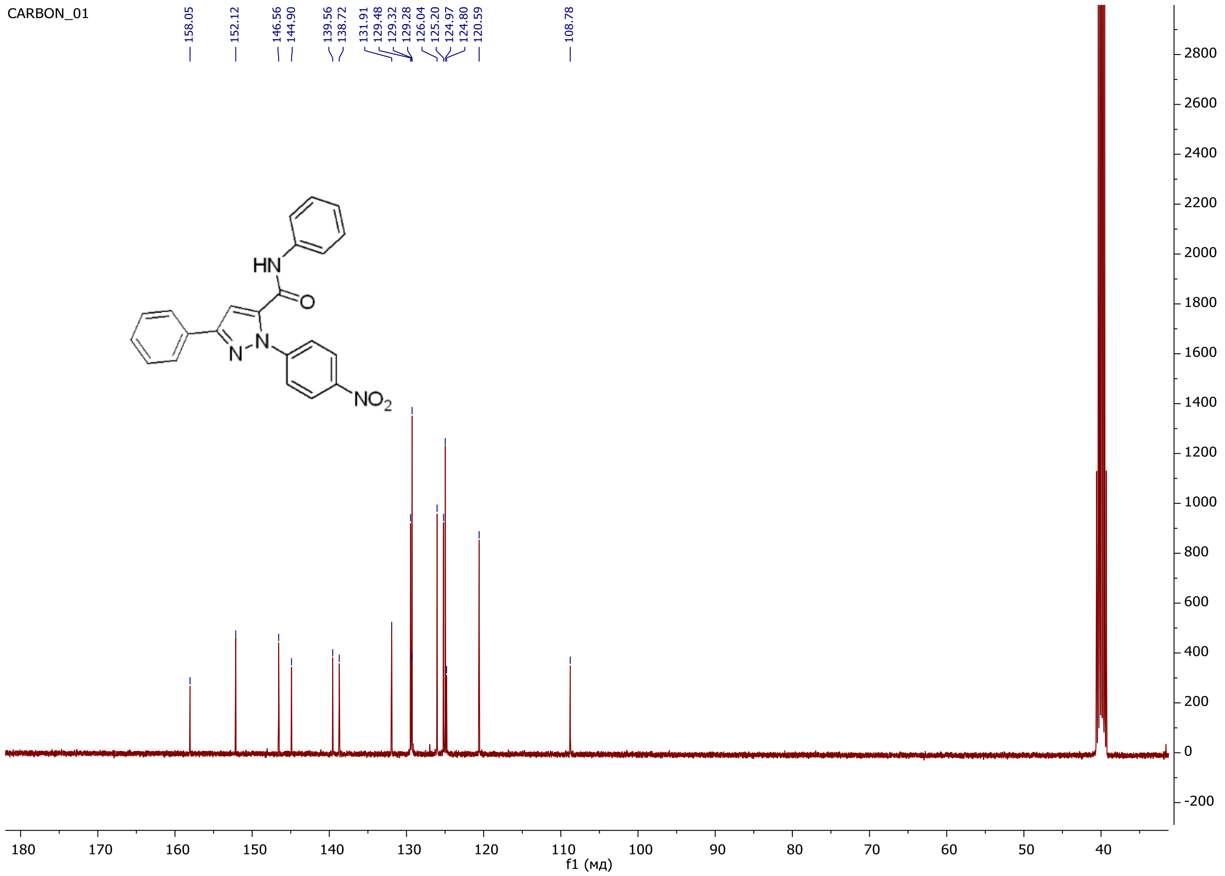

HSQC ^{1}H-^{13}C NMR spectra of compound **9a**

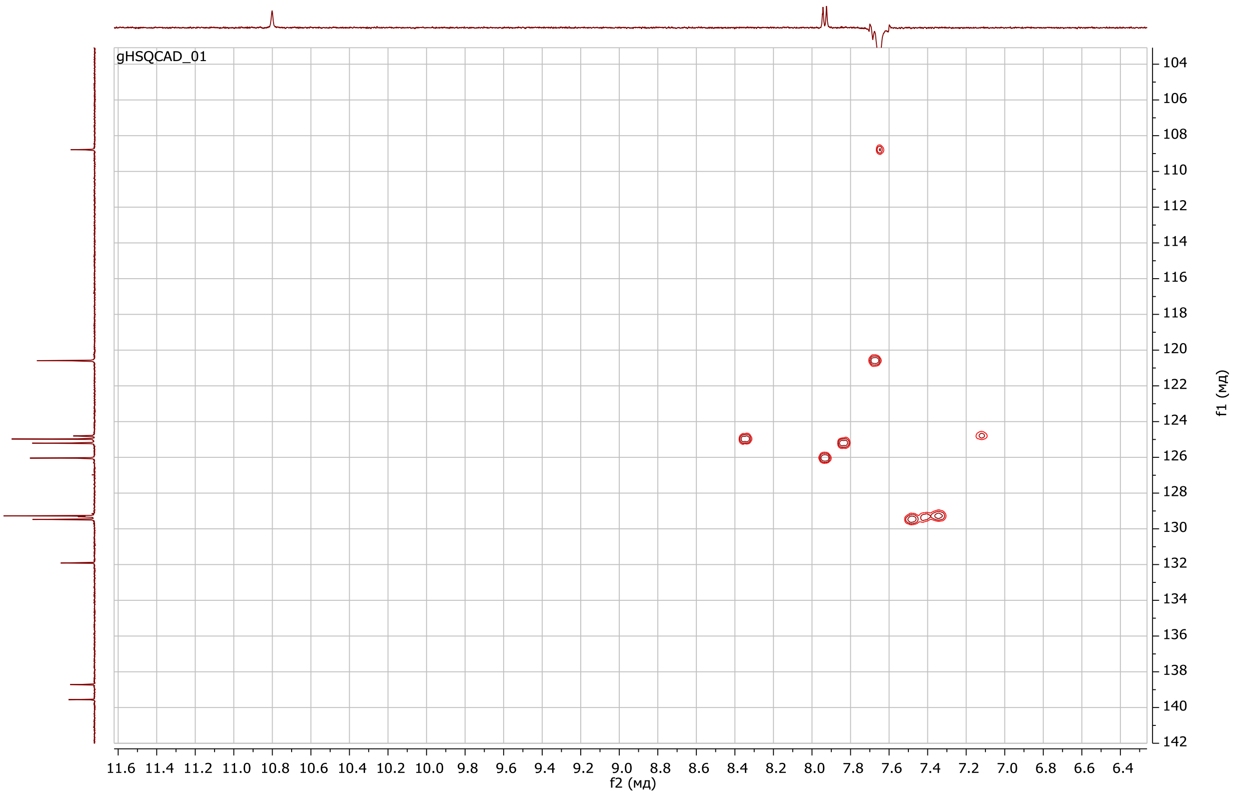

HMBC ^{1}H-^{13}C NMR spectra of compound **9a**

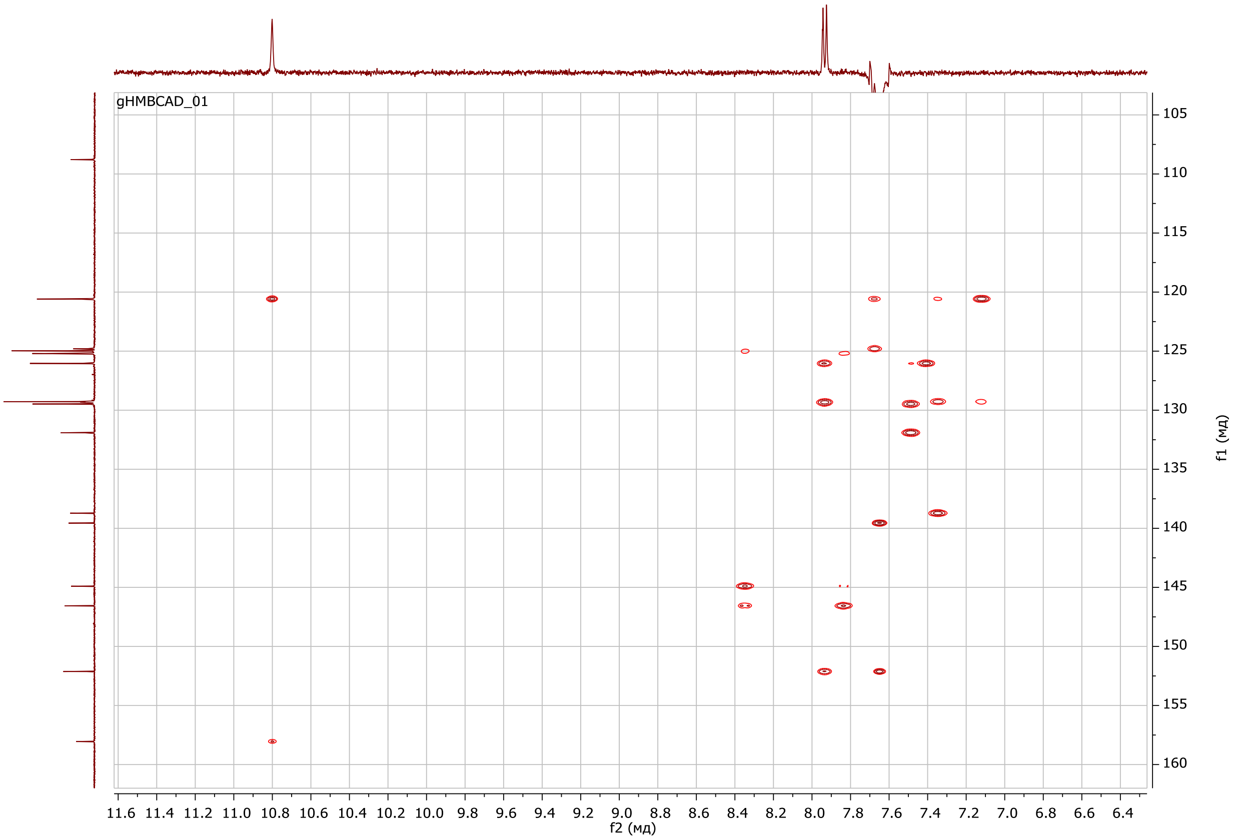

NOESY 1D NMR spectra of compound **9a**

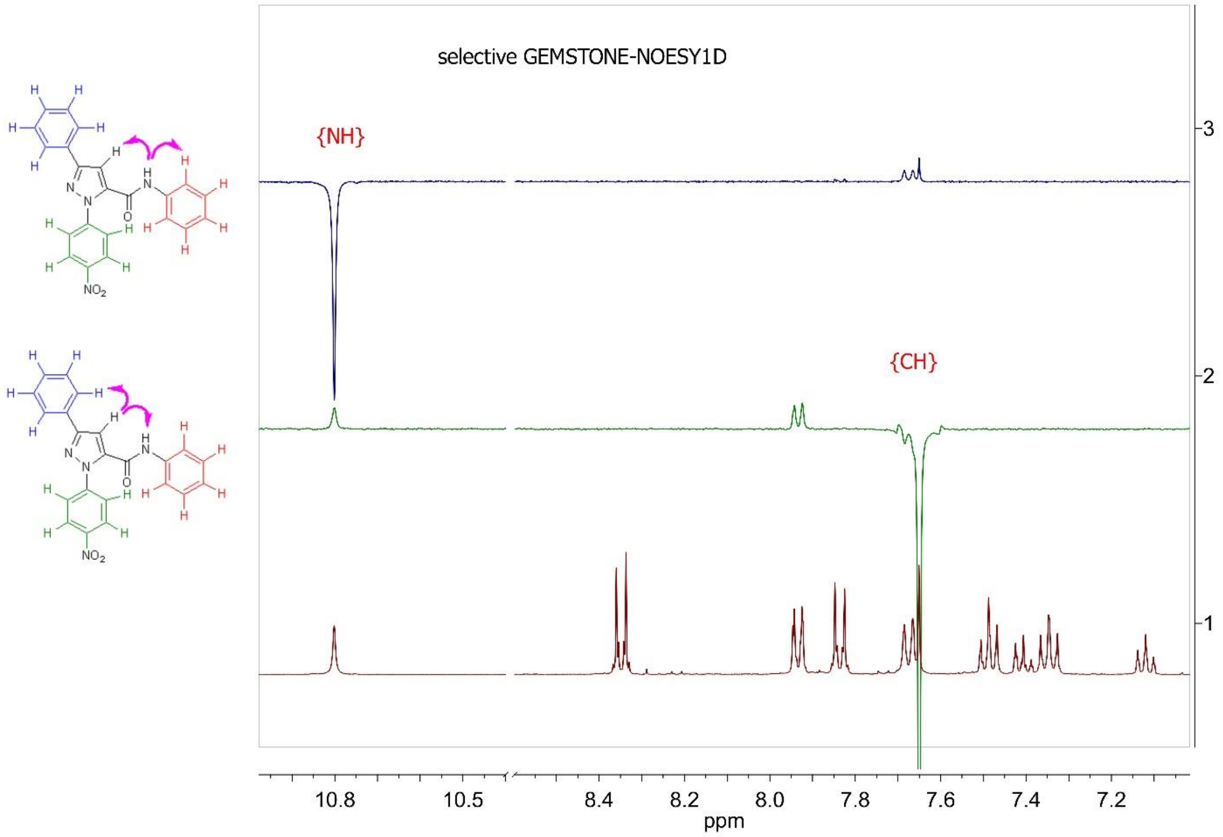

TOCSY NMR spectra of compound **9a**

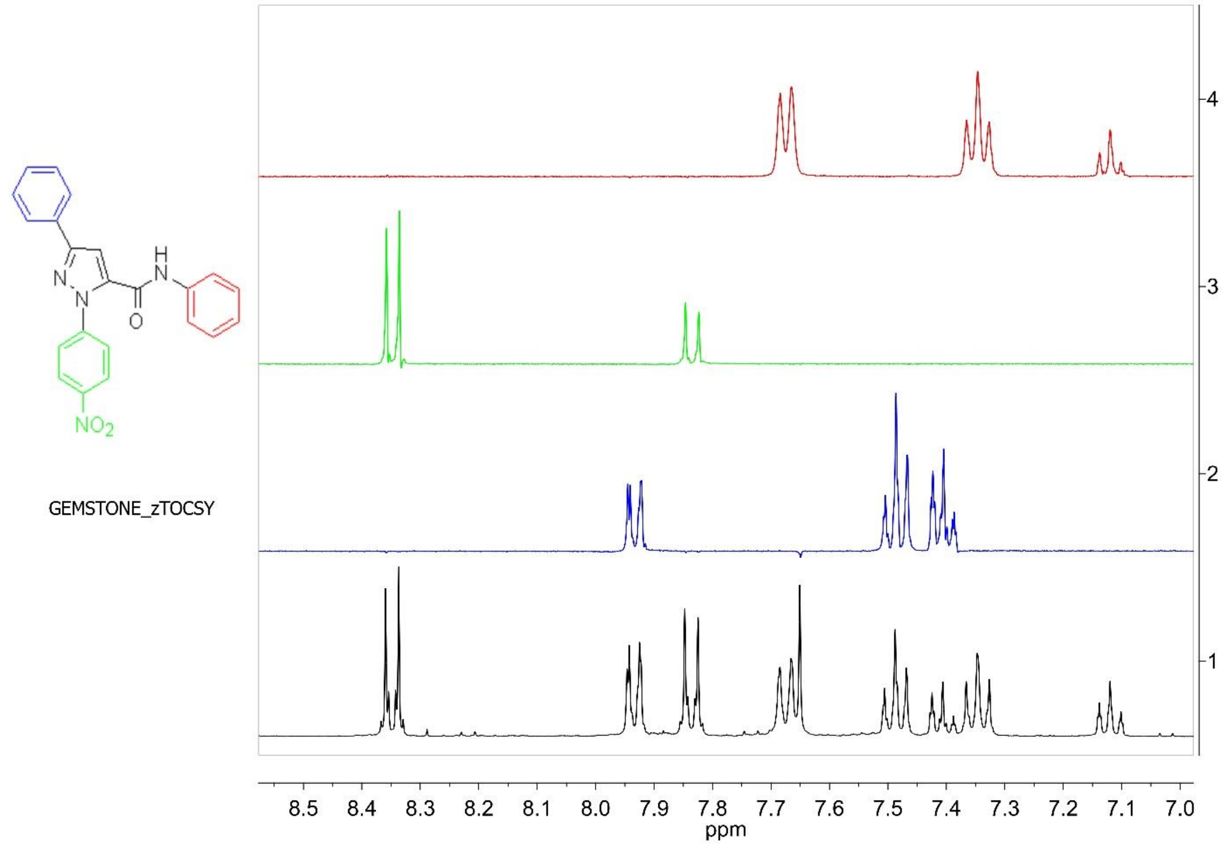

^{1}H NMR spectrum of **7m** (400 Hz, DMSO-d_6)

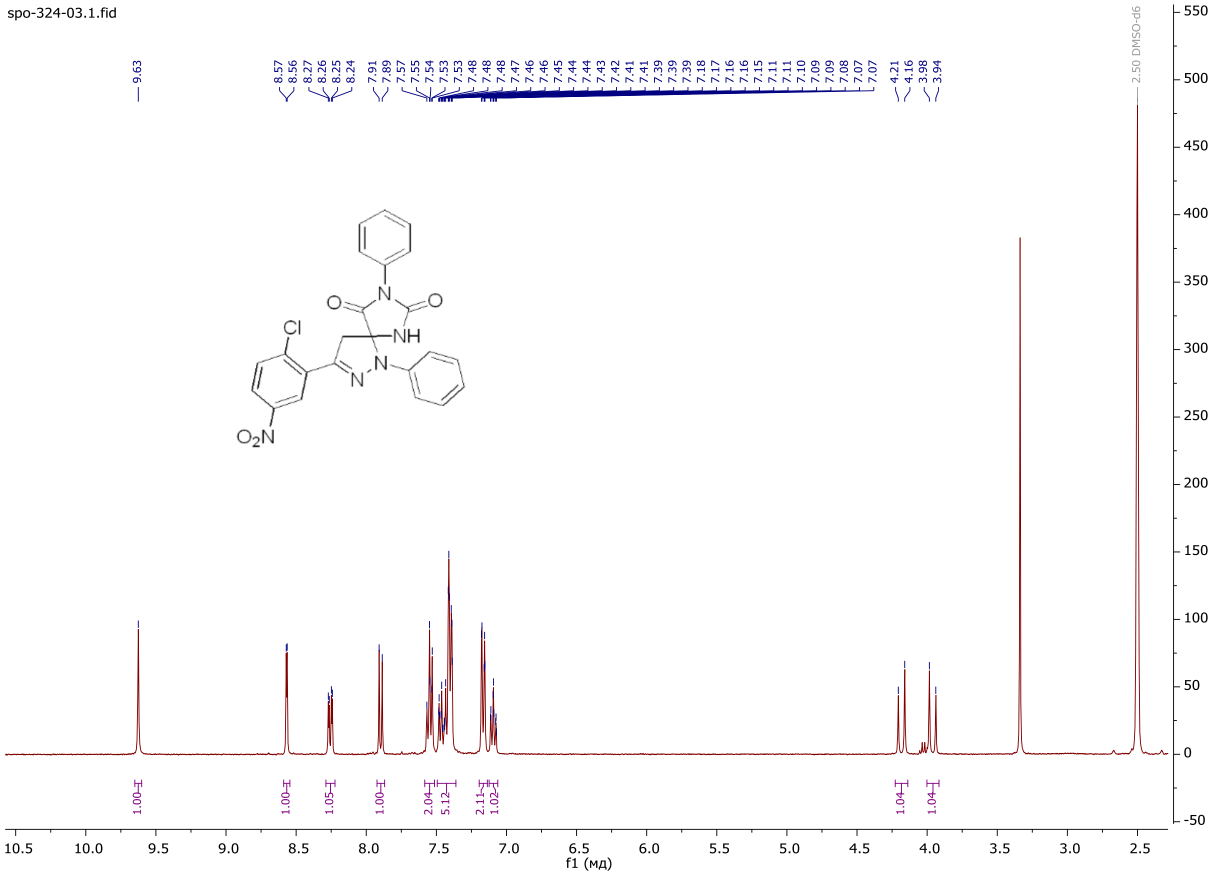

^{13}C NMR spectrum of **7m** (100 MHz, DMSO-d_6)

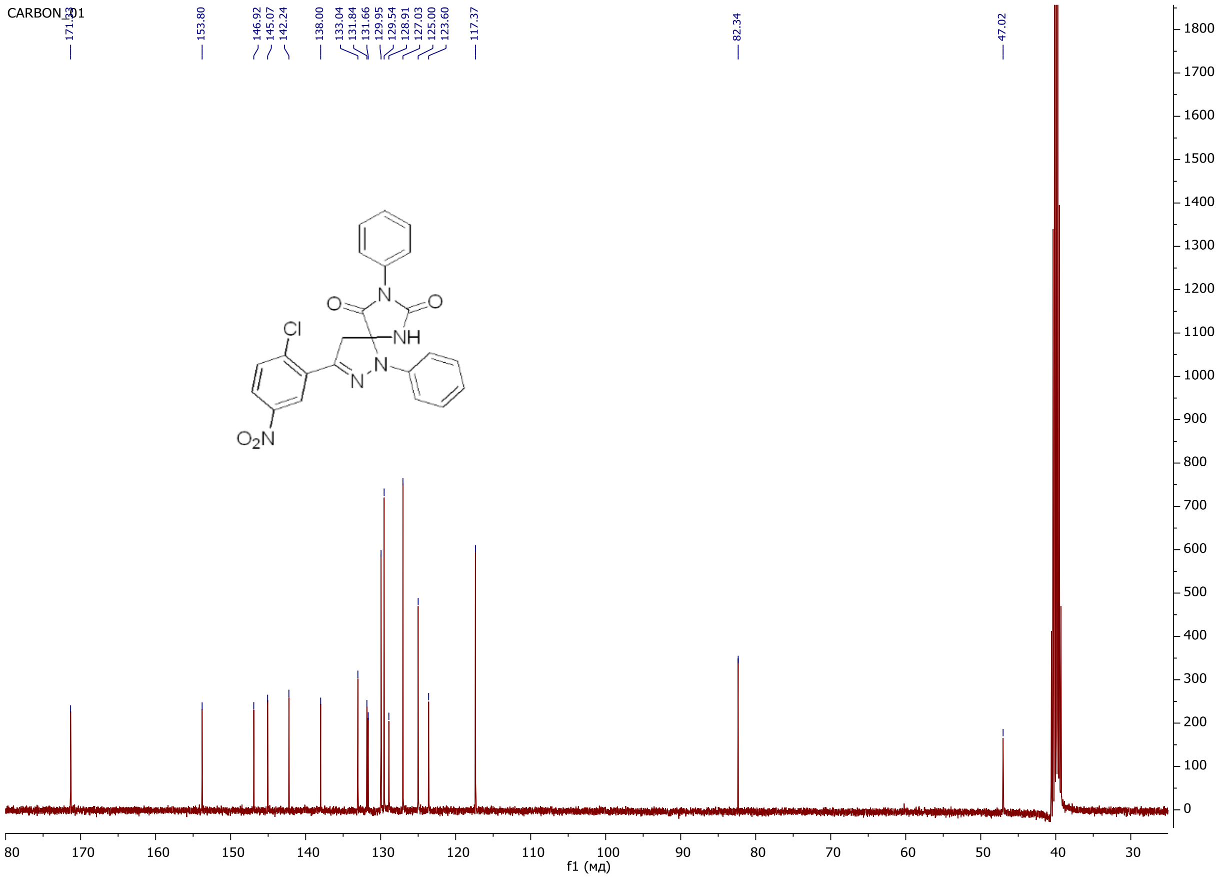

^{1}H NMR spectrum of **7n** (400 Hz, DMSO-d_6)

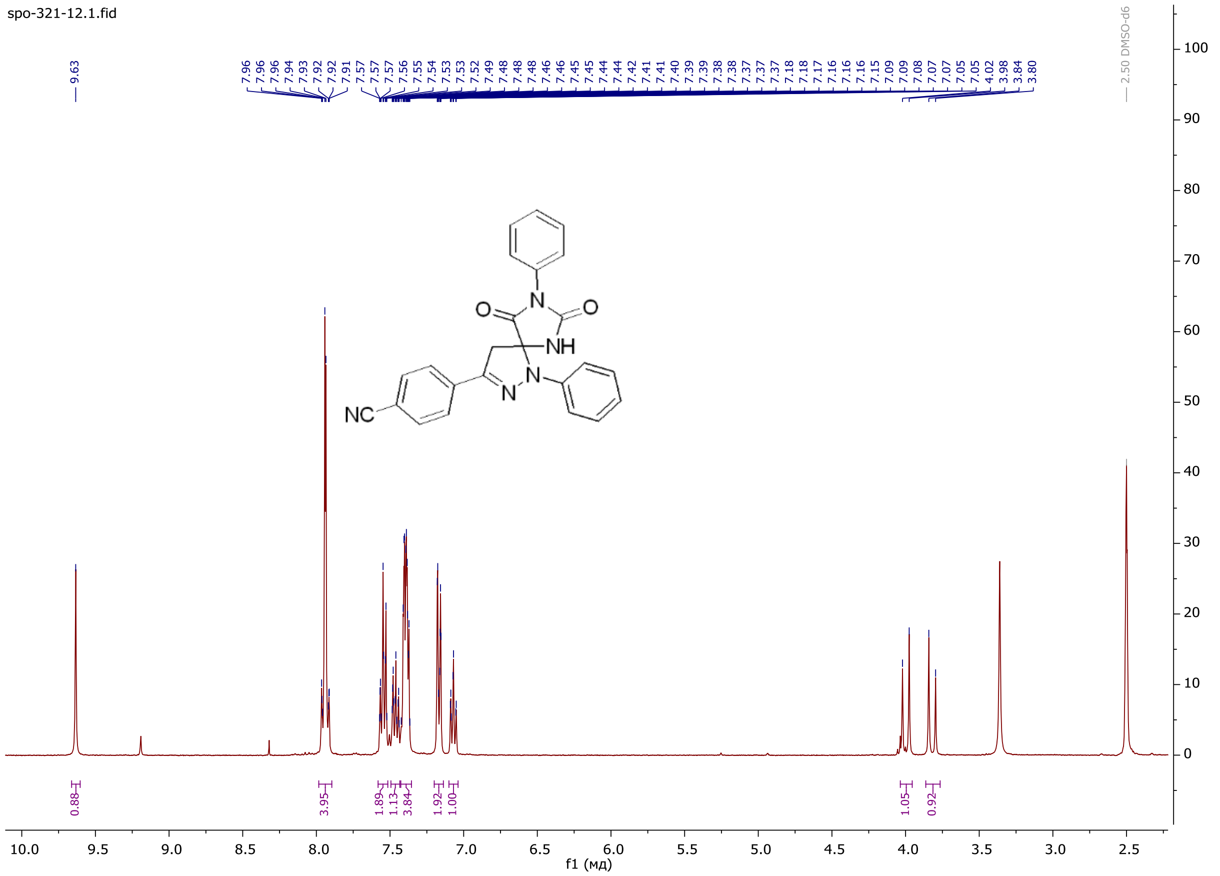

^{13}C NMR spectrum of **7n** (100 MHz, DMSO-d_6)

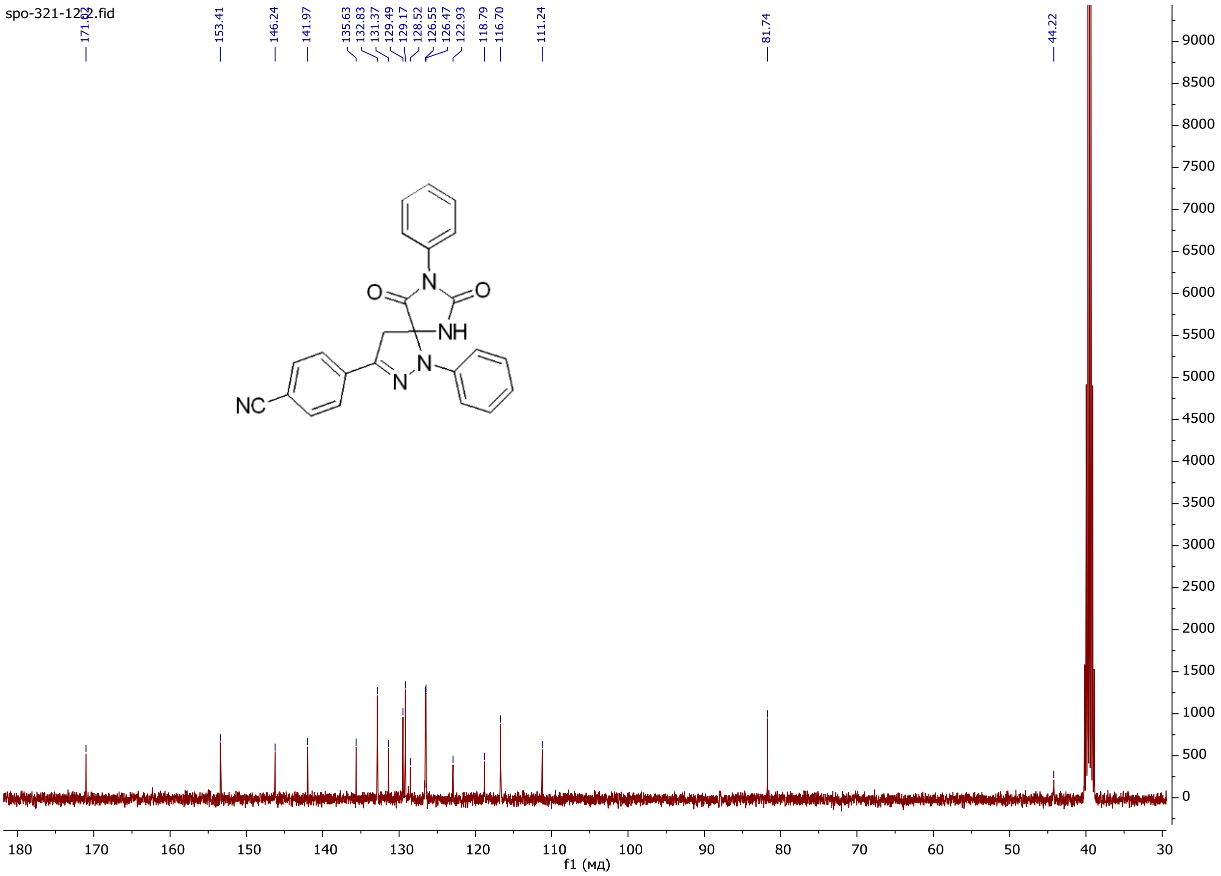

^{1}H NMR spectrum of **7p** (400 Hz, DMSO-d_6)

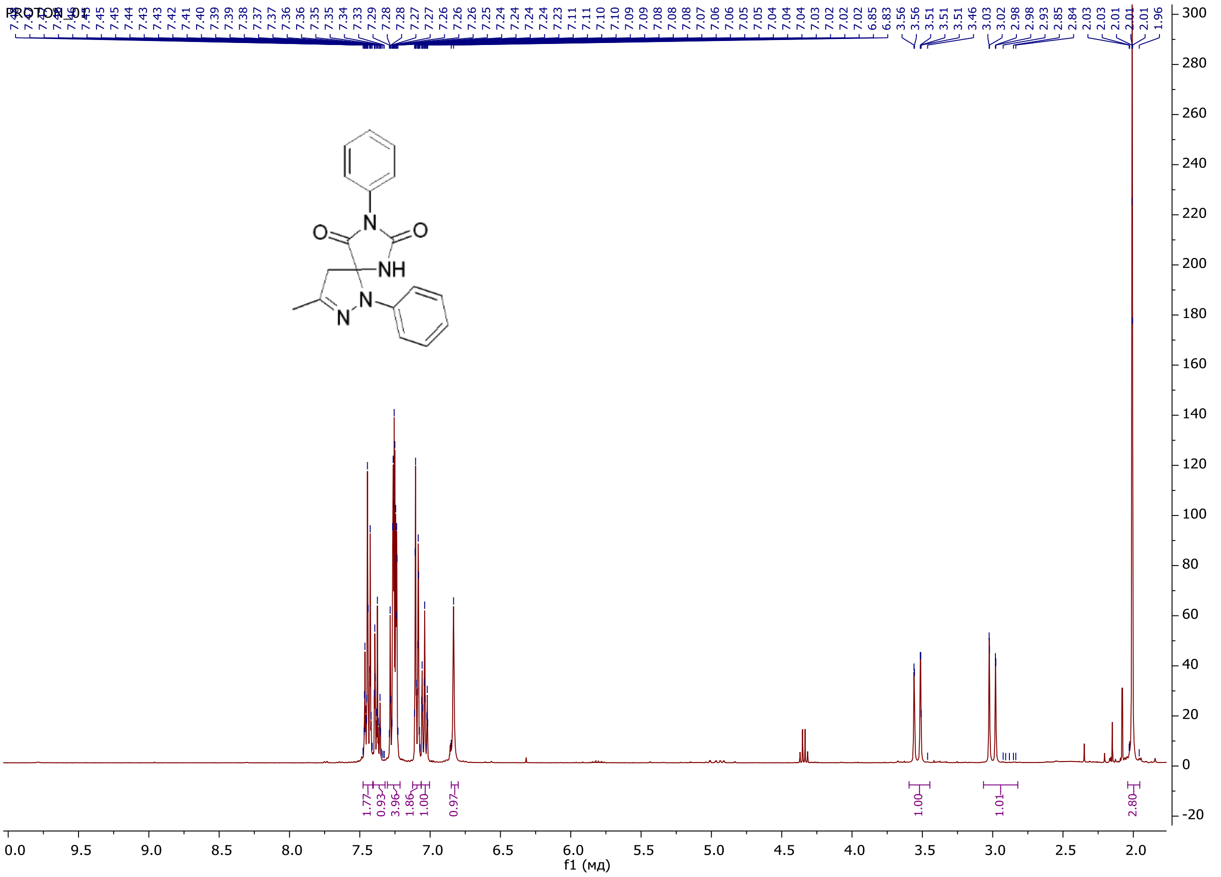

^{13}C NMR spectrum of **7p** (100 MHz, DMSO-d_6)

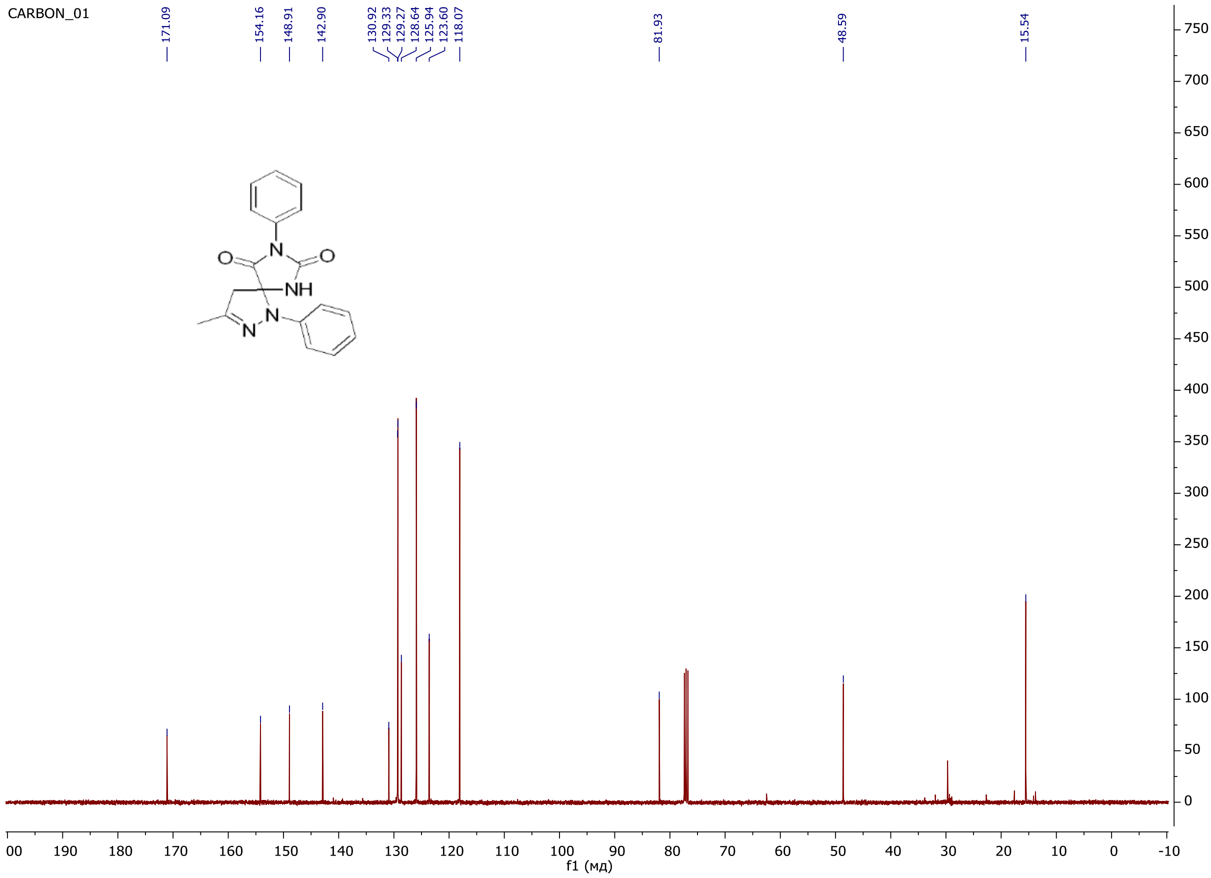

DFT calculations

Table S1: Single-point absolute energies and coordinates of nitrile imines and 5-methylene-3-phenylhydantoin **6** at the PBE0/def-2svpd level of theory in gas phase

Structure	Absolute Energies in Hartree	Cartesian Coordinates			
**6**	-644.704158	C	-2.982190000	1.665456000	0.326629000
		C	-1.762803000	2.433988000	-0.083497000
		N	-1.005857000	1.541457000	-0.823651000
		C	-1.596622000	0.302900000	-0.921389000
		N	-2.810727000	0.390462000	-0.209903000
		O	-3.913140000	2.060232000	0.980571000
		O	-1.162489000	-0.658464000	-1.500612000
		C	-3.725009000	-0.685159000	-0.069956000
		C	-3.251995000	-1.977864000	0.168175000
		C	-4.158791000	-3.025324000	0.301591000
		C	-5.529713000	-2.789561000	0.213585000
		C	-5.993193000	-1.494881000	-0.013161000
		C	-5.097165000	-0.440483000	-0.163546000
		C	-1.522643000	3.707780000	0.237768000
		H	-0.113343000	1.724307000	-1.255139000

		H	-2.180777000	-2.160125000	0.232922000
		H	-3.786692000	-4.035163000	0.482827000
		H	-6.236302000	-3.613901000	0.321664000
		H	-7.064745000	-1.299944000	-0.082307000
		H	-5.456651000	0.571744000	-0.339493000
		H	-2.261326000	4.236010000	0.838788000
		H	-0.619772000	4.229845000	-0.078160000
**5a** (R_1 = Cl)	-1069.010560	N	-5.164766000	0.873418000	-0.208999000
		N	-5.945654000	-0.059093000	0.059874000
		C	-6.569300000	-1.010022000	0.339049000
		C	-7.312140000	-2.185608000	0.591573000
		C	-6.651596000	-3.391445000	0.901648000
		C	-7.378134000	-4.549600000	1.136276000
		C	-8.770758000	-4.519407000	1.068341000
		C	-9.442723000	-3.334039000	0.770890000
		C	-8.720119000	-2.173902000	0.533860000
		C	-5.704427000	2.114394000	-0.537897000
		C	-4.795452000	3.132685000	-0.868627000
		C	-5.255136000	4.398055000	-1.209730000
		C	-6.623862000	4.677340000	-1.227644000
		C	-7.528613000	3.669432000	-0.896416000
		C	-7.080899000	2.396811000	-0.553683000
		Cl	-9.675224000	-5.970311000	1.357247000
		H	-5.562917000	-3.413675000	0.955124000
		H	-6.865262000	-5.481565000	1.373427000
		H	-10.531329000	-3.322180000	0.722537000
		H	-9.244236000	-1.247903000	0.297020000
		H	-3.729331000	2.902766000	-0.852368000
		H	-4.535893000	5.178598000	-1.465656000
		H	-6.980722000	5.672355000	-1.497487000
		H	-8.600966000	3.875237000	-0.904768000
		H	-7.800244000	1.617427000	-0.295876000
**5j** (R_1 = CF_3)	-946.191174	C	-0.742817000	-1.365630000	0.172957000
		C	-0.059338000	-2.516659000	0.563506000
		C	-0.784359000	-3.618295000	1.023718000
		C	-2.170875000	-3.573254000	1.093382000
		C	-2.861686000	-2.415871000	0.699556000
		C	-2.131853000	-1.308147000	0.236993000
		N	-4.251693000	-2.451965000	0.796683000
		N	-4.897410000	-1.443690000	0.460169000
		C	-5.616781000	-0.568088000	0.162098000

		C	-6.495672000	0.500906000	-0.118898000
		C	-7.887606000	0.338942000	0.052221000
		C	-8.746584000	1.392968000	-0.210921000
		C	-8.244636000	2.622955000	-0.649621000
		C	-6.871579000	2.787531000	-0.831789000
		C	-5.999875000	1.739201000	-0.572629000
		C	-9.205147000	3.749556000	-0.910402000
		F	-10.077444000	3.441214000	-1.883526000
		F	-8.581280000	4.873402000	-1.276884000
		F	-9.935930000	4.036605000	0.179219000
		H	-0.187090000	-0.498121000	-0.188602000
		H	1.029500000	-2.555917000	0.509486000
		H	-0.260975000	-4.525172000	1.332897000
		H	-2.747835000	-4.426793000	1.451622000
		H	-2.653942000	-0.401787000	-0.074832000
		H	-8.281797000	-0.617341000	0.396079000
		H	-9.820298000	1.261444000	-0.070666000
		H	-6.481559000	3.744523000	-1.176712000
		H	-4.927677000	1.872798000	-0.715113000
**5k** (R_1 = NO_2)	-813.855626	C	-6.804552000	-0.137556000	0.910427000
		N	-6.363176000	0.948502000	0.884273000
		N	-5.887937000	2.090496000	0.986797000
		C	-7.316102000	-1.448597000	0.945124000
		C	-5.707464000	2.842236000	-0.174364000
		C	-7.664538000	-2.108264000	-0.255362000
		C	-8.164113000	-3.397816000	-0.221315000
		C	-8.317572000	-4.037199000	1.008096000
		C	-7.982257000	-3.408617000	2.206418000
		C	-7.483103000	-2.118080000	2.178318000
		C	-5.154660000	4.122826000	-0.017360000
		C	-4.945570000	4.932908000	-1.126258000
		C	-5.283158000	4.486503000	-2.405835000
		C	-5.832854000	3.214364000	-2.563752000
		C	-6.046314000	2.393027000	-1.461312000
		N	-8.843755000	-5.395968000	1.040166000
		O	-8.966720000	-5.927451000	2.125756000
		O	-9.127474000	-5.914888000	-0.021325000
		H	-7.537994000	-1.595218000	-1.208393000
		H	-8.439894000	-3.924602000	-1.133554000
		H	-8.118559000	-3.944505000	3.144678000
		H	-7.216300000	-1.614020000	3.107030000

		H -4.897334000 4.459440000 0.987638000
		H -4.514855000 5.926816000 -0.991149000
		H -5.119445000 5.126846000 -3.273621000
		H -6.101136000 2.855506000 -3.559290000
		H -6.478853000 1.400385000 -1.597740000
**5l** (R_2 = NO_2)	-813.861235	N -6.337930000 -1.347439000 -1.326064000
		N -6.377904000 -0.100749000 -1.195520000
		C -6.345628000 1.066049000 -1.178335000
		C -6.873674000 -2.118430000 -0.315485000
		C -6.802406000 -3.517613000 -0.477273000
		C -7.311748000 -4.364564000 0.488368000
		C -7.904793000 -3.822142000 1.631594000
		C -7.989041000 -2.442495000 1.814949000
		C -7.475797000 -1.594699000 0.848377000
		C -6.242397000 2.477719000 -1.109459000
		C -6.535587000 3.150251000 0.092637000
		C -6.431123000 4.533910000 0.151925000
		C -6.037382000 5.262421000 -0.971240000
		C -5.748213000 4.598343000 -2.163827000
		C -5.850213000 3.215229000 -2.243139000
		N -8.447150000 -4.707230000 2.644773000
		O -8.973613000 -4.202011000 3.619331000
		O -8.347028000 -5.906701000 2.465243000
		H -6.336349000 -3.912898000 -1.379901000
		H -7.265604000 -5.447028000 0.378978000
		H -8.458386000 -2.056716000 2.718763000
		H -7.538782000 -0.515271000 0.990672000
		H -6.841628000 2.580593000 0.970526000
		H -6.658973000 5.048367000 1.086704000
		H -5.955114000 6.348806000 -0.916792000
		H -5.441565000 5.164294000 -3.044922000
		H -5.627792000 2.695264000 -3.175564000

Table S2: Single-point absolute energies and coordinates of nitrile imines and 5-methylene-3-phenylhydantoin **6** at the PBE0/def-2svpd level of theory using CPCM (CH_2Cl_2)

Structure	Absolute Energies in Hartree	Cartesian Coordinates
**6**	-644.721769	C -2.914802000 1.613061000 0.438815000
		C -1.747254000 2.412757000 -0.043798000
		N -1.092540000 1.589443000 -0.949213000
		C -1.712966000 0.382059000 -1.091867000
		N -2.828523000 0.405066000 -0.232433000

		O	-3.757659000	1.932357000	1.246522000
		O	-1.390811000	-0.536147000	-1.811225000
		C	-3.733419000	-0.678189000	-0.082613000
		C	-3.260667000	-1.924118000	0.332955000
		C	-4.155879000	-2.981462000	0.481280000
		C	-5.515413000	-2.791314000	0.230327000
		C	-5.980373000	-1.540634000	-0.178165000
		C	-5.090570000	-0.481268000	-0.343491000
		C	-1.444146000	3.652534000	0.351264000
		H	-0.259671000	1.829700000	-1.469638000
		H	-2.198124000	-2.057570000	0.538688000
		H	-3.789013000	-3.957609000	0.803909000
		H	-6.214686000	-3.620223000	0.353088000
		H	-7.042527000	-1.388802000	-0.378957000
		H	-5.440077000	0.497050000	-0.675520000
		H	-2.088825000	4.143577000	1.079306000
		H	-0.574001000	4.183044000	-0.035530000
**5a** (R_1 = Cl)	-1069.021941	C	-2.512461000	-3.183742000	-0.226493000
		C	-2.169277000	-2.912547000	1.097661000
		C	-2.879987000	-1.979325000	1.852086000
		C	-3.947138000	-1.305836000	1.274906000
		C	-4.306502000	-1.563748000	-0.062504000
		C	-3.578486000	-2.510711000	-0.808116000
		C	-5.390242000	-0.867287000	-0.649210000
		N	-6.276949000	-0.280405000	-1.129397000
		N	-7.216391000	0.287408000	-1.739027000
		C	-7.909931000	1.279959000	-1.048448000
		C	-8.956512000	1.923187000	-1.733569000
		C	-9.692120000	2.925552000	-1.111272000
		C	-9.405214000	3.310923000	0.201775000
		C	-8.368292000	2.673725000	0.884998000
		C	-7.624205000	1.667254000	0.273792000
		Cl	-0.830670000	-3.753201000	1.821610000
		H	-1.947400000	-3.916615000	-0.802817000
		H	-2.601696000	-1.779657000	2.886887000
		H	-4.508339000	-0.574845000	1.857478000
		H	-3.850366000	-2.715037000	-1.844194000
		H	-9.176898000	1.621535000	-2.759341000
		H	-10.500935000	3.414259000	-1.658853000
		H	-9.985278000	4.098597000	0.685563000
		H	-8.132555000	2.962338000	1.911793000

		H	-6.818734000	1.178216000	0.824700000
**5j** ($R_1 = CF_3$)	-946.202928	C	-0.810716000	-1.318118000	0.143138000
		C	-0.084948000	-2.454304000	0.504254000
		C	-0.769343000	-3.578825000	0.975065000
		C	-2.155536000	-3.570135000	1.083356000
		C	-2.889531000	-2.427543000	0.718503000
		C	-2.199791000	-1.296444000	0.245580000
		N	-4.276059000	-2.500271000	0.852248000
		N	-4.941173000	-1.490004000	0.526020000
		C	-5.659728000	-0.614656000	0.241304000
		C	-6.517960000	0.469200000	-0.057512000
		C	-7.914792000	0.325431000	0.071998000
		C	-8.747022000	1.393755000	-0.222329000
		C	-8.205194000	2.611871000	-0.648192000
		C	-6.823888000	2.758628000	-0.781333000
		C	-5.979273000	1.696172000	-0.490201000
		C	-9.135317000	3.748353000	-0.960470000
		F	-9.961674000	3.450733000	-1.980120000
		F	-8.485632000	4.867435000	-1.297046000
		F	-9.924205000	4.050829000	0.086265000
		H	-0.289586000	-0.432034000	-0.225915000
		H	1.002978000	-2.464635000	0.419218000
		H	-0.215279000	-4.475272000	1.261845000
		H	-2.694620000	-4.445659000	1.450152000
		H	-2.750831000	-0.399464000	-0.043312000
		H	-8.335691000	-0.623979000	0.403546000
		H	-9.826966000	1.277793000	-0.118558000
		H	-6.402295000	3.706860000	-1.113486000
		H	-4.900166000	1.809600000	-0.594030000
**5k** ($R_1 = NO_2$)	-813.870991	C	-6.795822000	-0.122833000	0.964205000
		N	-6.355010000	0.959824000	0.934527000
		N	-5.876656000	2.108861000	1.026431000
		C	-7.311094000	-1.433583000	0.977601000
		C	-5.706960000	2.837063000	-0.152719000
		C	-7.649729000	-2.065435000	-0.239936000
		C	-8.149989000	-3.354650000	-0.232935000
		C	-8.312046000	-4.015947000	0.985929000
		C	-7.986071000	-3.409889000	2.200541000
		C	-7.486494000	-2.119556000	2.199627000
		C	-5.153646000	4.122752000	-0.027537000
		C	-4.954309000	4.911542000	-1.155097000

		C	-5.302192000	4.438495000	-2.423547000
		C	-5.852340000	3.161553000	-2.549629000
		C	-6.056724000	2.361126000	-1.428721000
		N	-8.832744000	-5.369264000	0.988733000
		O	-8.960575000	-5.934668000	2.060129000
		O	-9.117790000	-5.877511000	-0.081203000
		H	-7.514762000	-1.533200000	-1.181367000
		H	-8.415350000	-3.854497000	-1.163022000
		H	-8.125275000	-3.952898000	3.134111000
		H	-7.226531000	-1.631870000	3.139080000
		H	-4.886321000	4.485938000	0.966472000
		H	-4.523347000	5.908602000	-1.042723000
		H	-5.145542000	5.060796000	-3.306265000
		H	-6.128404000	2.780523000	-3.535148000
		H	-6.489964000	1.365799000	-1.543445000
**5l** ($R_2 = NO_2$)	-813.878993	N	-6.484083000	-1.315806000	-1.337824000
		N	-6.459879000	-0.063235000	-1.148979000
		C	-6.390674000	1.097430000	-1.119838000
		C	-6.950825000	-2.103517000	-0.318135000
		C	-6.961886000	-3.500087000	-0.549240000
		C	-7.408722000	-4.371936000	0.420441000
		C	-7.858511000	-3.866049000	1.648756000
		C	-7.859915000	-2.489130000	1.900158000
		C	-7.411786000	-1.615148000	0.928353000
		C	-6.268291000	2.509447000	-1.084382000
		C	-6.739915000	3.231164000	0.028443000
		C	-6.609578000	4.614644000	0.049588000
		C	-6.018315000	5.284838000	-1.023506000
		C	-5.554506000	4.567502000	-2.128238000
		C	-5.674644000	3.183490000	-2.168545000
		N	-8.323158000	-4.770711000	2.659637000
		O	-8.711167000	-4.310839000	3.725323000
		O	-8.313997000	-5.970597000	2.419735000
		H	-6.607649000	-3.873870000	-1.510566000
		H	-7.417849000	-5.446677000	0.245319000
		H	-8.213758000	-2.118826000	2.861378000
		H	-7.413651000	-0.542822000	1.127611000
		H	-7.201599000	2.701877000	0.862738000
		H	-6.974188000	5.174201000	0.912657000
		H	-5.918453000	6.371302000	-0.999011000
		H	-5.094118000	5.090935000	-2.967889000

		H -5.314701000 2.617681000 -3.028666000

Table S3: Minimum-point absolute energies and coordinates of reactants, products and transition states for the reaction of nitrile imine **5a** and 5-methylene-3-phenylhydantoin **6** at the PBE0/def-2svpd level of theory using CPCM (CH_2Cl_2)

Structure	Absolute Energies in Hartree	Cartesian Coordinates
**5a** and **6**	-1713.746625	N -0.512860000 0.561428000 1.448779000
		H -2.655460000 -0.425892000 -2.188061000
		C -0.341640000 0.293308000 -3.661171000
		H 1.723410000 -2.582942000 0.139129000
		C -1.113820000 2.792388000 0.729419000
		C -0.619380000 -0.687632000 -2.797751000
		N -0.378420000 -2.539082000 -1.475061000
		H 0.286050000 -1.755332000 2.260499000
		H 2.646650000 -4.593882000 1.286109000
		H 0.658200000 0.346998000 -4.090411000
		C -2.857560000 4.324878000 -0.057611000
		H -3.587920000 3.515798000 -0.024841000
		Cl -2.802630000 8.214438000 -0.994651000
		C -3.248700000 5.594708000 -0.460371000
		C -0.979510000 6.401848000 -0.135591000
		C -0.584280000 5.133708000 0.265859000
		N -0.773800000 1.735288000 1.087429000
		C 0.130210000 -3.683672000 -0.807211000
		C 0.786370000 0.308218000 1.885719000
		C 1.804850000 1.277868000 1.935239000
		H 3.854610000 1.701088000 2.423159000
		C 3.075950000 0.935708000 2.390709000
		C 3.360870000 -0.367262000 2.801939000
		C 1.077330000 -1.004242000 2.299539000
		C 1.255870000 -3.559642000 0.009079000
		C 0.015690000 -6.040692000 -0.324491000
		H -0.469960000 -7.009332000 -0.456181000
		C -0.491910000 -4.921152000 -0.980561000
		O -2.542890000 -2.539642000 -0.602981000
		H 1.543710000 -6.807252000 0.994819000
		O 1.486950000 -1.908982000 -2.702481000
		C 0.336740000 -1.750792000 -2.360701000
		N -1.801270000 -0.962882000 -2.124041000
		H -1.085210000 1.038418000 -3.944081000
		H -4.283890000 5.784438000 -0.744861000

		C	-2.308680000	6.624218000	-0.494791000
		H	-0.255330000	7.215808000	-0.169911000
		C	-1.520320000	4.081958000	0.310439000
		H	0.452830000	4.950978000	0.548149000
		H	1.601240000	2.301328000	1.615059000
		H	4.358600000	-0.630002000	3.157559000
		H	2.558300000	-2.356722000	3.068729000
		C	2.350700000	-1.332612000	2.750989000
		C	1.765090000	-4.687462000	0.649319000
		C	1.145270000	-5.927152000	0.487039000
		H	-1.366320000	-5.000332000	-1.627391000
		C	-1.693750000	-2.060212000	-1.319511000
**7a**	-1713.842657	N	0.315997000	0.902196000	0.850771000
		H	-2.161660000	-0.404421000	1.465796000
		C	-1.216257000	0.951802000	-0.902073000
		H	2.087995000	-3.336718000	-0.179974000
		C	-0.810207000	2.296847000	-0.370732000
		C	-0.527001000	-0.010967000	0.090638000
		N	-0.350545000	-2.307104000	-0.337742000
		H	1.001326000	-1.585952000	1.686756000
		H	2.798571000	-5.557361000	-1.055142000
		H	-0.838332000	0.810546000	-1.925456000
		C	-2.191882000	3.617179000	-1.955580000
		H	-2.551016000	2.690809000	-2.405395000
		Cl	-2.744667000	7.540398000	-2.535762000
		C	-2.645107000	4.830071000	-2.467146000
		C	-1.289497000	6.006931000	-0.832698000
		C	-0.845625000	4.792387000	-0.330568000
		N	0.050046000	2.201824000	0.579979000
		C	0.047075000	-3.578393000	-0.832514000
		C	1.245908000	0.573628000	1.834685000
		C	1.944705000	1.593405000	2.505659000
		H	3.417250000	2.085519000	3.982843000
		C	2.887840000	1.275742000	3.476621000
		C	3.163768000	-0.052313000	3.806405000
		C	1.519794000	-0.762484000	2.170395000
		C	1.372356000	-3.989387000	-0.680973000
		C	-0.490271000	-5.645519000	-1.941457000
		H	-1.219502000	-6.291529000	-2.433640000
		C	-0.888091000	-4.397453000	-1.466383000
		O	-2.081179000	-3.007730000	1.057476000

		H	1.141568000	-7.039164000	-2.179125000
		O	1.175392000	-0.933357000	-1.407714000
		C	0.247509000	-1.114777000	-0.659694000
		N	-1.429240000	-0.799253000	0.890915000
		H	-2.301488000	0.789456000	-0.915479000
		H	-3.347607000	4.850891000	-3.300402000
		C	-2.187751000	6.015994000	-1.901491000
		H	-0.941886000	6.944915000	-0.399410000
		C	-1.286986000	3.577498000	-0.884031000
		H	-0.144200000	4.778606000	0.503563000
		H	1.741389000	2.632493000	2.255809000
		H	3.906181000	-0.294250000	4.568208000
		H	2.667136000	-2.110484000	3.378807000
		C	2.471805000	-1.062805000	3.142424000
		C	1.762760000	-5.233433000	-1.171447000
		C	0.833459000	-6.063492000	-1.799281000
		H	-1.917707000	-4.058369000	-1.581931000
		C	-1.392128000	-2.122419000	0.605490000
**TS-I**	-1713.731032	N	-0.028946000	0.770056000	1.168230000
		H	-2.775782000	-0.456812000	0.101821000
		C	-1.099291000	0.828773000	-1.788129000
		H	2.087034000	-3.294818000	0.043691000
		C	-0.744249000	2.617605000	-0.141253000
		C	-1.015190000	-0.322096000	-1.096144000
		N	-0.395668000	-2.447597000	-0.560760000
		H	0.722372000	-1.560033000	1.888276000
		H	2.950361000	-5.600608000	-0.345441000
		H	-0.254471000	1.153816000	-2.392558000
		C	-2.410384000	3.940035000	-1.439551000
		H	-2.933162000	3.044217000	-1.771905000
		Cl	-2.801808000	7.878588000	-2.029331000
		C	-2.878920000	5.173562000	-1.874571000
		C	-1.135554000	6.281630000	-0.605041000
		C	-0.668070000	5.050103000	-0.170295000
		N	-0.289714000	1.890475000	0.673802000
		C	0.126088000	-3.755543000	-0.729099000
		C	1.121122000	0.556121000	1.922667000
		C	1.979169000	1.568603000	2.384210000
		H	3.709555000	2.029571000	3.572972000
		C	3.060410000	1.236997000	3.196860000
		C	3.314841000	-0.091797000	3.545682000

		C	1.386435000	-0.781817000	2.263503000
		C	1.442965000	-4.061756000	-0.387469000
		C	-0.228623000	-6.017332000	-1.491237000
		H	-0.885063000	-6.771909000	-1.927808000
		C	-0.707800000	-4.727797000	-1.288694000
		O	-2.261950000	-2.965371000	0.742645000
		H	1.463339000	-7.345676000	-1.312541000
		O	1.152918000	-1.200692000	-1.765128000
		C	0.088516000	-1.306944000	-1.195516000
		N	-2.013695000	-0.943446000	-0.350416000
		H	-2.052920000	1.336386000	-1.887131000
		H	-3.743837000	5.225394000	-2.535763000
		C	-2.234622000	6.337074000	-1.462798000
		H	-0.644966000	7.199315000	-0.277324000
		C	-1.296302000	3.860678000	-0.582306000
		H	0.193389000	4.997931000	0.493498000
		H	1.778801000	2.609605000	2.127930000
		H	4.156477000	-0.341532000	4.196738000
		H	2.664301000	-2.143518000	3.319546000
		C	2.474946000	-1.099764000	3.064276000
		C	1.919801000	-5.354934000	-0.607847000
		C	1.087283000	-6.333985000	-1.150562000
		H	-1.728499000	-4.462589000	-1.569024000
		C	-1.650635000	-2.198169000	0.029293000
**8a**	-1713.665649	C	0.082422000	-1.188053000	0.637153000
		C	0.262992000	0.328341000	0.381151000
		N	1.694359000	0.484679000	0.553718000
		C	2.345567000	-0.662822000	0.906490000
		N	1.341743000	-1.695564000	0.937529000
		C	-0.620666000	1.153690000	1.357544000
		N	-1.248490000	2.144216000	0.476409000
		N	-1.059832000	1.870631000	-0.819830000
		C	-0.263851000	0.839091000	-0.976339000
		O	3.530004000	-0.821598000	1.150517000
		C	1.624568000	-3.060058000	1.241266000
		C	2.725563000	-3.698283000	0.636688000
		C	2.997856000	-5.040832000	0.939580000
		C	2.172058000	-5.752052000	1.826533000
		C	1.071265000	-5.110920000	2.419373000
		C	0.796358000	-3.764302000	2.137017000
		O	-0.975093000	-1.793417000	0.602300000

		C	-2.055620000	3.201838000	0.904922000
		C	0.109998000	0.349926000	-2.303157000
		C	-0.384565000	1.000943000	-3.465377000
		C	-0.051460000	0.556846000	-4.746644000
		C	0.793279000	-0.559605000	-4.894429000
		C	1.298859000	-1.225777000	-3.767579000
		C	0.957526000	-0.770731000	-2.486792000
		C	-2.630630000	4.099064000	-0.029812000
		C	-3.433692000	5.153715000	0.419585000
		C	-3.680722000	5.343080000	1.792273000
		C	-3.109324000	4.453006000	2.716260000
		C	-2.303834000	3.387351000	2.287233000
		Cl	1.214674000	-1.119671000	-6.496907000
		H	2.189342000	1.374343000	0.462658000
		H	-0.015520000	1.628019000	2.154063000
		H	-1.400056000	0.512263000	1.822330000
		H	3.365073000	-3.142012000	-0.062269000
		H	3.860794000	-5.536188000	0.468620000
		H	2.386547000	-6.807021000	2.055916000
		H	0.420253000	-5.660226000	3.116729000
		H	-0.059213000	-3.259987000	2.606613000
		H	-1.044389000	1.872539000	-3.349567000
		H	-0.443142000	1.071593000	-5.635940000
		H	1.956912000	-2.097839000	-3.890237000
		H	1.368862000	-1.315143000	-1.626355000
		H	-2.436562000	3.954712000	-1.100850000
		H	-3.873148000	5.841975000	-0.319485000
		H	-4.312240000	6.175872000	2.136186000
		H	-3.291806000	4.582528000	3.794481000
		H	-1.873016000	2.701840000	3.030435000
**TS-II**	-1713.573942	C	0.290001000	-1.113871000	1.312428000
		C	0.616747000	0.335510000	1.444754000
		N	1.929910000	0.471992000	0.990298000
		C	2.541622000	-0.751242000	0.805908000
		N	1.517762000	-1.738075000	1.013368000
		C	-0.197079000	1.211537000	2.072030000
		N	-2.368802000	1.619660000	0.389037000
		N	-1.614004000	1.312315000	-0.555541000
		C	-0.751568000	0.782527000	-1.211868000
		O	3.710827000	-0.967804000	0.528122000
		C	1.754366000	-3.132443000	1.186021000

		C	2.710604000	-3.819857000	0.414183000
		C	2.955806000	-5.178426000	0.674345000
		C	2.244655000	-5.858255000	1.676241000
		C	1.281383000	-5.169360000	2.431716000
		C	1.041326000	-3.811436000	2.198109000
		O	-0.797062000	-1.658000000	1.459318000
		C	-2.673190000	2.951584000	0.714645000
		C	-0.184832000	0.356312000	-2.446997000
		C	-0.496798000	1.083710000	-3.625264000
		C	0.030660000	0.700960000	-4.858205000
		C	0.879015000	-0.419312000	-4.927259000
		C	1.218304000	-1.145458000	-3.771765000
		C	0.696209000	-0.753950000	-2.534154000
		C	-2.700197000	4.023993000	-0.208278000
		C	-3.048085000	5.311306000	0.227979000
		C	-3.351708000	5.556118000	1.578810000
		C	-3.324182000	4.491470000	2.497857000
		C	-3.005772000	3.198755000	2.070244000
		Cl	1.507732000	-0.918423000	-6.475575000
		H	2.450002000	1.351341000	0.953154000
		H	0.063309000	2.268823000	2.217744000
		H	-1.158234000	0.827483000	2.432700000
		H	3.277385000	-3.288500000	-0.361034000
		H	3.719563000	-5.708648000	0.085786000
		H	2.446499000	-6.920474000	1.878002000
		H	0.719920000	-5.681757000	3.227758000
		H	0.301700000	-3.272044000	2.802376000
		H	-1.164067000	1.953674000	-3.551114000
		H	-0.225860000	1.264258000	-5.765538000
		H	1.890623000	-2.012396000	-3.845885000
		H	0.965566000	-1.324438000	-1.633927000
		H	-2.468502000	3.837523000	-1.267008000
		H	-3.089895000	6.133491000	-0.503278000
		H	-3.613965000	6.569900000	1.914355000
		H	-3.546225000	4.667496000	3.562020000
		H	-2.981469000	2.362432000	2.783382000

References

1. Tietze, L.F.; Eicher, T. *Reaktionen Und Synthesen Im Organisch-Chemischen Praktikum Und Forschungslaboratorium (2. Auflage)*; 117th ed.; Wiley: Thieme Verlag, Stuttgart, Germany; New York, NY, USA, 1991; ISBN 3527308741.

2. Dascalu, A.E.; Rouleau Billamboz, M.; Guinet, A.; Rigo, B.; Lipka Belloli, E.; Hartkoorn, R.C.; Ple, C. Hydrazide Derivatives and Their Specific Use as Antibacterial Agents by Controlling Acinetobacter Baumannii Bacterium 2020.

3. Wang, W.J.; Zhang, T.; Duan, L.J.; Zhang, X.J.; Yan, M. KOt-Bu Promoted Homocoupling and Decomposition of N′-Aryl Acylhydrazines: Synthesis of Unsymmetric N′,N′-Diaryl Acylhydrazines. *Tetrahedron* **2015**, *71*, 9073–9080, doi:10.1016/J.TET.2015.10.023.

4. Morrill, L.C.; Lebl, T.; Slawin, A.M.Z.; Smith, A.D. Catalytic Asymmetric α-Amination of Carboxylic Acids Using Isothioureas. *Chem. Sci.* **2012**, *3*, 2088–2093, doi:10.1039/C2SC20171B.

5. Tanimori, S.; Kobayashi, Y.; Iesaki, Y.; Ozaki, Y.; Kirihata, M. Copper-Catalyzed Synthesis of Substituted Indazoles from 2-Chloroarenes at Low Catalyst-Loading. *Org. Biomol. Chem.* **2012**, *10*, 1381–1387, doi:10.1039/C1OB05875D.

6. Catarzi, D.; Varano, F.; Poli, D.; Squarcialupi, L.; Betti, M.; Trincavelli, L.; Martini, C.; Dal Ben, D.; Thomas, A.; Volpini, R.; et al. 1,2,4-Triazolo[1,5-a]Quinoxaline Derivatives and Their Simplified Analogues as Adenosine A3 Receptor Antagonists. Synthesis, Structure–Affinity Relationships and Molecular Modeling Studies. *Bioorg. Med. Chem.* **2015**, *23*, 9–21, doi:10.1016/J.BMC.2014.11.033.

7. Zhang, J.Q.; Huang, G. Bin; Weng, J.; Lu, G.; Chan, A.S.C. Copper(II)-Catalyzed Coupling Reaction: An Efficient and Regioselective Approach to N′,N′-Diaryl Acylhydrazines. *Org. Biomol. Chem.* **2015**, *13*, 2055–2063, doi:10.1039/C4OB02343A.

8. Zhang, Y.; Tang, Q.; Luo, M. Reduction of Hydrazines to Amines with Aqueous Solution of Titanium(III) Trichloride. *Org. Biomol. Chem.* **2011**, *9*, 4977–4982, doi:10.1039/C1OB05328K.

9. Gao, L.M.; Wang, X.M.; Wei, Q.L.; Su, K.X.; Huang, R.H.; Guo, J.; Zheng, Y.S.; Liu, J.K. [3+3] Cycloadditions of Azomethine Ylides with Nitrile Imines for the Synthesis of 2,3,4,5-Tetrahydro-1,2,4-Triazine-5-Carboxylates. *European J. Org. Chem.* **2022**, *2022*, doi:10.1002/EJOC.202200768.

10. El-Abadelah, M.M.; Hussein, A.Q.; Kamal, M.R.; Al-Adhami, K.H. Heterocycles from Nitrile Imines. Part I. 1,2,3,4-Tetrahydro-1,2,4,5-Tetrazines. *Heterocycles* **1988**, *27*, 917–924, doi:10.3987/COM-87-4434.

11. Dadiboyena, S.; Valente, E.J.; Hamme, A.T. A Novel Synthesis of 1,3,5-Trisubstituted Pyrazoles through a Spiro-Pyrazoline Intermediate via a Tandem 1,3-Dipolar Cycloaddition/Elimination. *Tetrahedron Lett.* **2009**, *50*, 291–294, doi:10.1016/J.TETLET.2008.10.145.

12. Rector, D.L.; Folz, S.D.; Conklin, R.D.; Nowakowski, L.H.; Kaugars, G. Structure-Activity Relationships in a Broad-Spectrum Anthelmintic Series. Acid Chloride

Phenylhydrazones. 1. Aryl Substitutions and Chloride Variations. *J. Med. Chem.* **1981**, *24*, 532–538, doi:10.1021/JM00137A011/SUPPL_FILE/JM00137A011_SI_001.PDF.

13. Bonini, B.F.; Franchini, M.C.; Gentili, D.; Locatelli, E.; Ricci, A. 1,3-Dipolar Cycloaddition of Nitrile Imines with Functionalized Acetylenes: Regiocontrolled Sc(OTf)3-Catalyzed Synthesis of 4- and 5-Substituted Pyrazoles. *Synlett* **2009**, *2009*, 2328–2332, doi:10.1055/S-0029-1217714/ID/9B.

14. Reinov, M. V.; Yurovskaya, M.A.; Davydov, D. V.; Streletskii, A. V. Heterocyclic Derivatives of Fullerene C60. 1. Synthesis of New Fulleropyrazolines by the 1,3-Dipolar Cycloaddition of Nitrile Imines. *Chem. Heterocycl. Compd.* **2004**, *40*, 188–193, doi:10.1023/B:COHC.0000027890.05668.07/METRICS.

15. Tewari, R.S.; Parihar, P. Halogenation of Substituted Hydrarones. A Facile Route for the Synthesis of Some New Hydrazidoyl Halides. *J. Chem. Eng. Data* **1981**, *26*, 418–420.
